# Iron photocatalysis towards site-selective C(sp^3^)–H alkylation of glycines and peptides

**DOI:** 10.1039/d5sc07730c

**Published:** 2025-10-22

**Authors:** Satya Prakash Panda, M. Siva Prasad, Prahallad Meher, Oliver Reiser, Sandip Murarka

**Affiliations:** a Department of Chemistry, Indian Institute of Technology Jodhpur Karwar-342037 Rajasthan India sandipmurarka@iitj.ac.in; b Department of Organic Chemistry, University of Regensburg Universitätsstr 31 93053 Regensburg Germany oliver.reiser@chemie.uni-regensburg.de

## Abstract

Utilizing iron-mediated ligand-to-metal charge transfer (LMCT) photocatalysis provides a sustainable platform for generating carbon-centered radicals. This study presents the use of abundant and inexpensive iron as a photocatalyst to easily activate aliphatic carboxylic acids, producing Csp^3^ radicals for site-selective C(sp^3^)–H alkylation of glycines and peptides. The method exhibits broad applicability, cost-effectiveness, and excellent tolerance to various functional groups. It is a flexible and efficient approach applicable to the synthesis of various unnatural α-amino acids and enables peptide drug bioconjugation. In addition to secondary and tertiary radicals, the iron-LMCT system enables access to primary radicals and the highly unstable methyl radical, derived from acetic acid, facilitating the production of methylated glycine derivatives. Preliminary mechanistic studies suggest a reaction pathway involving an Fe(iii)–Fe(ii)–Fe(iii) redox cycle and radical–radical cross-coupling.

## Abstract 2

Utilizing iron-mediated ligand-to-metal charge transfer (LMCT) photocatalysis provides a sustainable platform for the site-selective alkylation of gylcines and peptides.

## Graphical abstract

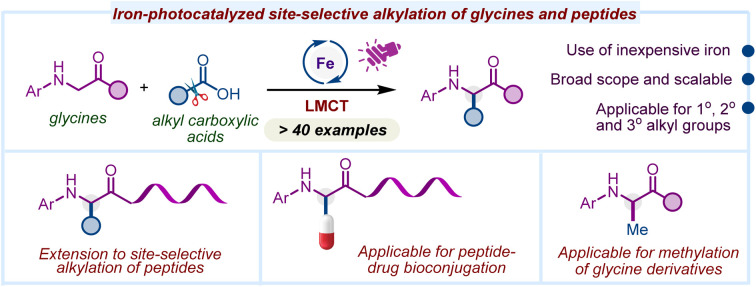

## Introduction

**Scheme 1:**
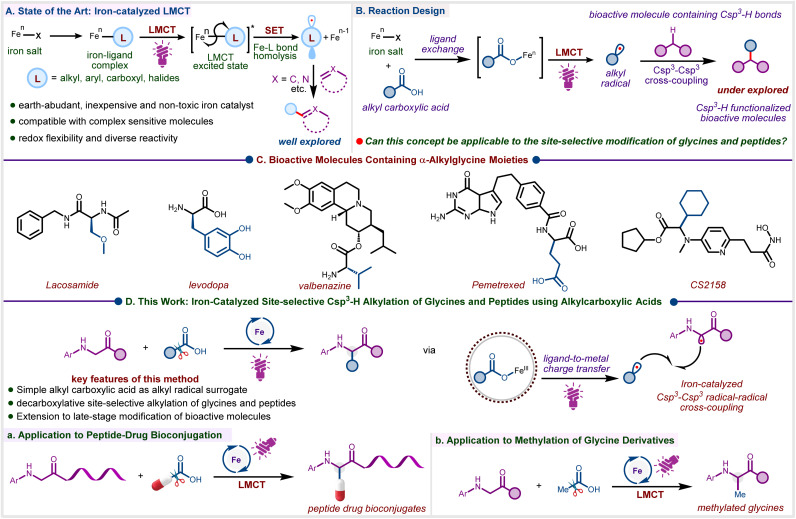
(A) Reaction Design. (B) Bioactive molecules containing α-Alkyglycine Moieties. (C) This work: iron-catalyzed site-selective Csp^3^–H alkylation of glycines and peptides using alkyl carboxylic acids.

Driven by the need for sustainable synthesis, the development of efficient catalytic strategies to generate alkyl radicals from inexpensive and abundant feedstocks, and subsequently enabling versatile C–H functionalization for constructing molecular frameworks, has become a focal point in radical chemistry.^1–3^ Photocatalytic approaches for generating alkyl radicals are primarily based on the excitation of precious metal polypyridyl complexes *via* metal-to-ligand charge transfer (MLCT), which serves as the key step for single-electron transfer.^4–8^ Despite notable advancements, there is a compelling need for the development of new catalytic systems based on earth-abundant metals that enable the mild generation of alkyl radicals and offer precise control over subsequent transformations, while benefiting from low toxicity, cost-effectiveness, and broad natural availability.^9,10^ In this regard, photoinduced iron-catalyzed ligand-to-metal charge transfer (LMCT) is particularly attractive, as it has been proven to be a powerful and greener method for the generation of carbon-centred radicals *via* the activation of C–H and C-heteroatom bonds (Scheme 1A).^11–15^ Carboxylic acids, due to their stability, abundance, and commercial availability, serve as a fundamental feedstock for organic synthesis, providing a versatile platform for constructing complex molecular architectures *via* decarboxylative transformations.^16–18^ While carboxylic acids represent attractive precursors for accessing structurally diverse carbon-centred radicals, their broad utilization remains challenging due to the inherently high oxidation potential of carboxylate anions and the instability of the resulting, especially of primary and methyl, radicals that might obstruct decarboxylation.^19,20^ Consequently, establishing a sustainable and practical strategy to harness alkyl carboxylic acids as radical precursors is of significant interest for enabling the efficient generation of alkyl radicals. Leveraging iron-catalyzed decarboxylation *via* the inner sphere LMCT presents a promising approach to overcome this major obstacle (Scheme 1B).^21–23^

α-Alkylglycines are an important class of nonproteinogenic amino acids that offer remarkable medicinal properties and frequently occur in pharmaceuticals (Scheme 1C).^24–26^ Direct α-C–H functionalization of glycine and glycine-containing peptides represents one of the most efficient and convenient approaches for accessing a diverse array of unnatural amino acid derivatives.^27–29^ Accordingly, there are several reports on the alkylation of glycine derivatives under both thermal^30,31^ and photochemical^28,30,32–35^ conditions. Although notable regarding their scope, elevated temperatures, pre-activated alkyl precursors, or expensive ruthenium and palladium catalysts are generally required. In this context, Correa and co-workers presented a cobalt-catalyzed oxidative Csp^3^–H alkylation of glycines and peptides under thermal conditions, utilizing an aqueous solution of TBHP as the hydrogen atom transfer (HAT) mediator.^36^ However, this method is limited to tetrahydrofuran (THF) and 1,3-dioxolane as coupling partners. Similarly, the Ye group documented a copper-catalyzed cross-dehydrogenative coupling between glycines/peptides and alkanes (20 equiv) using di-*tert*-butyl peroxide (DTBP, 3 equiv) as the HAT mediator.^30^ On the other hand, Shang and co-workers achieved photoinduced alkylation of glycines and peptides using alkyl *N*-(acyloxy)phthalimides (NHPI esters)^37,38^ as alkyl radical precursors under metal-free conditions.^39^

Existing reports on photoinduced iron LMCT catalysis focus on the alkylation of unsaturated substrates such as alkenes and heteroarenes,^40–49^ the trapping of radicals with electrophiles,^50–52^ and the formation of carbon–carbon or carbon-heteroatom bonds *via* dual catalysis (Scheme 1A).^41,53–55^ However, the photoinduced Fe(iii)-catalyzed decarboxylative Csp^3^–Csp^3^ cross-coupling *via* LMCT phenomenon remains largely underexplored (Scheme 1B). Based on our interest in photodecarboxylative C–H alkylation^19,56,57^ and base metal-catalyzed photocatalysis,^9,58,59^ we report here a photoinduced iron-catalyzed LMCT protocol for the site-selective Csp^3^–H alkylation of glycines and peptides *via* radical–radical cross-coupling (Scheme 1D). During the preparation of this manuscript, Huo *et. al*. reported a CeCl_3_-photocatalyzed decarboxylative C(sp^3^)–H alkylation of glycine derivatives using DABCO as a base and DMSO as the terminal oxidant.^60^ Notably, under these conditions, Fe(iii) salts were found ineffective,^60^ in contrast to our findings where Fe(iii)Cl_3_ (10 mol%) serves as a cost-effective^61^ alternative to Ce(iii)Cl_3_ (20 mol%).^58,59^ Furthermore, our protocol not only enables the efficient transfer of secondary or tertiary alkyl but also more challenging primary moieties to glycines and peptides. Beyond glycine derivatives and short-chain peptides, our strategy has been further extended to long-chain peptides and peptide–drug conjugates (Scheme 1D(a)). The magic methyl effect,^62–64^ known for enhancing biological activity and pharmacokinetic properties, makes the selective installation of a methyl group on sp^2^ and sp^3^ carbons a highly desirable transformation in drug development. Gratifyingly, the LMCT approach reported here can be applied to highly reactive methyl radicals for site-selective methylation of glycine derivatives, using inexpensive and readily available acetic acid as the methylating agent (Scheme 1D(b)), which is, to the best of our knowledge, unprecedented.

## Results and discussion

To test the feasibility of the desired C(sp^3^)–C(sp^3^) cross-coupling, we initiated our investigation by reacting model substrates ethyl phenylglycinate 1a (1 equiv.), and cyclohexyl carboxylic acid 2a (2 equiv.) in the presence of FeCl_3_ (10 mol%) as the photocatalyst, and DABCO (2 equiv) as the base in acetonitrile under purple LED irradiation (390 nm). To our delight, the desired coupling product 3 was formed in 38% yield (Table 1, entry 1). Utilization of alternative iron catalysts, such as FeBr_3_ and Fe(NO_3_)_3_, failed to improve the yield (entries 2 and 3). Pleasingly, the yield escalated to 67% upon adding 2 equiv of 70% aqueous solution of *tert*-butyl hydroperoxide (TBHP) as an oxidant (entry 4). The addition of picolinic acid^42^ (30 mol%) as an additive led to a further increase in the yield of 3 (76%, entry 5). Replacing acetonitrile solvent with THF led to a significant decrease in the yield of 3 (entry 6). Notably, a higher loading of FeCl_3_ did not improve the outcome and gave nearly the same yield of 3 (entry 7). Switching from 390 nm purple LEDs to 427 nm blue LEDs led to a lower yield of 3 (entry 8). Control experiments confirmed the indispensable role of FeCl_3_, DABCO, and purple LED irradiation in this reaction (entries 9–11). To demonstrate the synthetic applicability of this method, a gram-scale reaction was conducted using model substrates 1a and 2a under the optimized conditions, yielding the final product 3 in an isolated yield of 63% (entry 12). Hence, after the systematic screening of the reaction parameters (see Table S1 in the SI for details), we found that the reaction was best conducted using FeCl_3_ (10 mol%), DABCO (2 equiv.), TBHP (2 equiv.), and picolinic acid (30 mol%) in acetonitrile under purple LED irradiation (390 nm).

**Table 1:** Optimization of the reaction conditions^a^

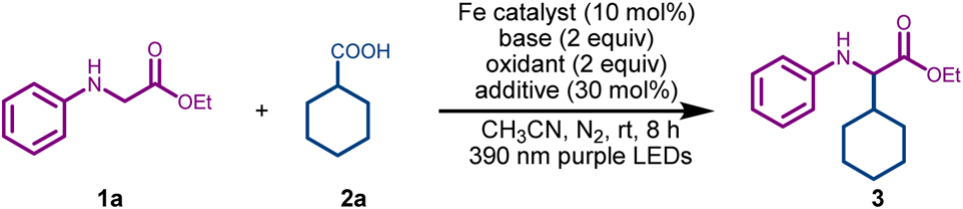					
Entry	Fe catalyst	Base	Oxidant	Additive	Yield (%)^b^
1	FeCl_3_	DABCO	—	—	38
2	FeBr_3_	DABCO	—	—	28
3	Fe(NO_3_)_3_·9H_2_O	DABCO	—	—	36
4	FeCl_3_	DABCO	TBHP	—	67
5	FeCl_3_	DABCO	TBHP	Picolinic acid	76
6^c^	FeCl_3_	DABCO	TBHP	Picolinic acid	32
7^d^	FeCl_3_	DABCO	TBHP	Picolinic acid	75
8^e^	FeCl_3_	DABCO	TBHP	Picolinic acid	42
9	—	DABCO	TBHP	Picolinic acid	ND
10	FeCl_3_	—	TBHP	Picolinic acid	Trace
11^f^	FeCl_3_	DABCO	TBHP	Picolinic acid	ND
12^g^	FeCl_3_	DABCO	TBHP	Picolinic acid	63

a Reaction conditions: 1a (0.15 mmol, 1 equiv.), 2a (2 equiv.), Fe catalyst (10 mol%), base (2 equiv.), oxidant (2 equiv.), additive (30 mol%) and CH_3_CN (1.5 mL) under nitrogen atmosphere using 390 nm purple LEDs for 8 h.

b Isolated yield.

c THF instead of CH_3_CN.

d 20 mol% FeCl_3_ was used.

e 427 nm blue LEDs instead of 390 nm purple LEDs.

f Without irradiation.

g Reaction was performed with 1 g of 1a. ND = not detected.

**Scheme 2:**
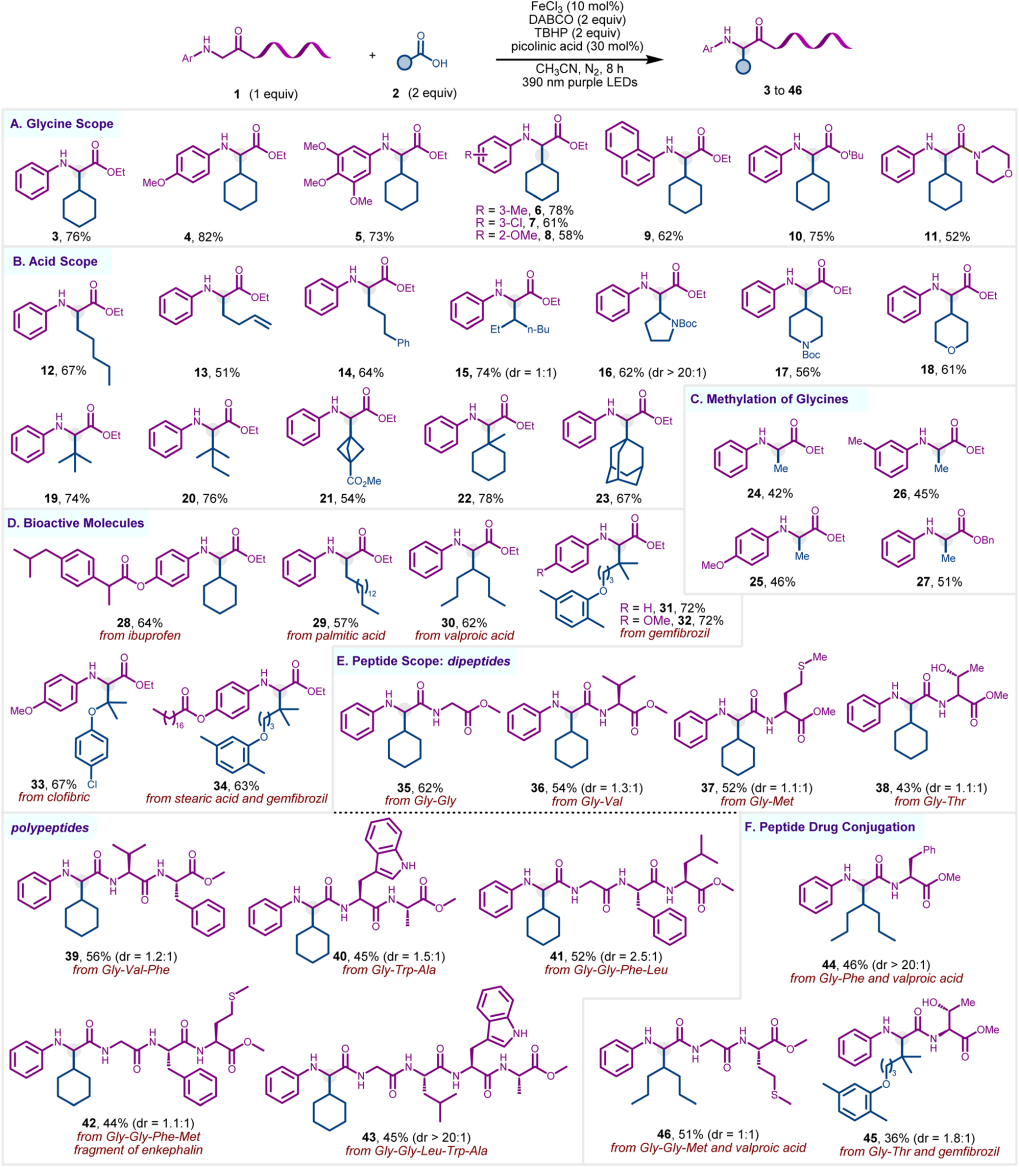
Scope of the α-Csp^3^–H alkylation of glycine and peptides. Reaction conditions: 1 (0.25 mmol, 1 equiv), 2 (2 equiv.), FeCl_3_ (10 mol%), DABCO (2 equiv.), 70% aqueous solution of TBHP (2 equiv.), picolinic acid (30 mol%), and CH_3_CN (2.5 mL) under a nitrogen atmosphere using purple LEDs (390 nm) for 8 h.

Next, we explored the scope of glycine derivatives by reacting them with cyclohexane carboxylic acid 2a under the optimized conditions (Scheme 2A). A variety of *N*-aryl glycine esters with various electron-donating and electron-withdrawing groups at different positions of the aryl ring were well tolerated, affording the corresponding products 4–8 (61–82%). Pleasingly, a fused aromatic system, such as *N*-naphthyl-based glycine derivative, also underwent smooth transformation, affording the final product 9 in 62% yield. The methodology was also applicable to the synthesis of α-alkylated glycines with *tert*-butyl ester and amide moieties 10–11. Subsequently, we explored the scope of alkyl carboxylic acids in their reaction with ethyl phenylglycinate 1a (Scheme 2B). A variety of primary, secondary, and tertiary carboxylic acids, including those with a sterically encumbered adamantane moiety, underwent facile transformation, furnishing 12–23 in moderate to good yields. To our delight, the reaction was found to be well-suited to *N*-Boc-proline as well, yielding 16 in 62% yield. Despite high synthetic relevance, the methylation of glycine derivatives remains scarcely explored, with one existing method utilizing bis-*tert*-butyl peroxide as a methyl radical source at elevated thermal (120 °C) conditions.^65^ We tested our protocol for the synthesis of methylated glycine derivatives using inexpensive and abundant acetic acid as a methylating agent. Pleasingly, a set of electronically and structurally diverse glycine esters was methylated in a site-selective manner to furnish the glycine derivatives 24–27 in acceptable yields (Scheme 2C). Control experiments in the absence of acetic acid that resulted in no product formation confirmed the latter as the methylating agent and especially ruled out THBP as the methyl radical source.^65^

This methodology was also expanded to the modification of various drugs and natural products derived from carboxylic acids, such as ibuprofen, palmitic acid, valproic acid, gemfibrozil, clofibric, and stearic acid, to afford the corresponding products 28–34, showcasing the prowess of the method in late-stage diversification of pharmacophores and bioactive motifs (Scheme 2D). Encouraged by these promising results, we further explored the method's potential for the site-selective alkylation of peptides (Scheme 2E). To our delight, a variety of glycine-derived dipeptides 35–38 and polypeptides 39–43 underwent site-selective alkylation at the α-position of glycine carbonyl to afford the corresponding cross-coupled products (43–62%), while retaining the integrity of other amino acids. Notably, peptides derived from reactive functional groups (–OH, –SCH_3_) containing amino acids, such as methionine and threonine, were well accommodated in the transformation. Recognizing the growing significance of peptide-drug bioconjugation in modern therapeutics, we probed our methodology for synthesizing peptide-drug bioconjugates. Accordingly, various peptides were cross-coupled with acid-based pharmaceuticals, such as gemfibrozil and valproic acid 44–46, thereby providing a novel approach for the discovery of peptide-based drug molecules (Scheme 2F).

We also evaluated the corresponding *N*-cyclohexyl and *N*-benzoyl glycine derivatives. Unfortunately, these substrates were unsuccessful, consistent with earlier reports, which do not include examples of glycine alkylation beyond *N*-aryl derivatives.^30,36^ This is due to the captodative-effect driven stability of the α-amino radicals generated in the case of *N*-aryl glycine derivatives. The selective removal of *N*-aryl substituents in the title compounds is nevertheless feasible, as was already demonstrated in previous reports.^29,30^

**Fig. 1:**
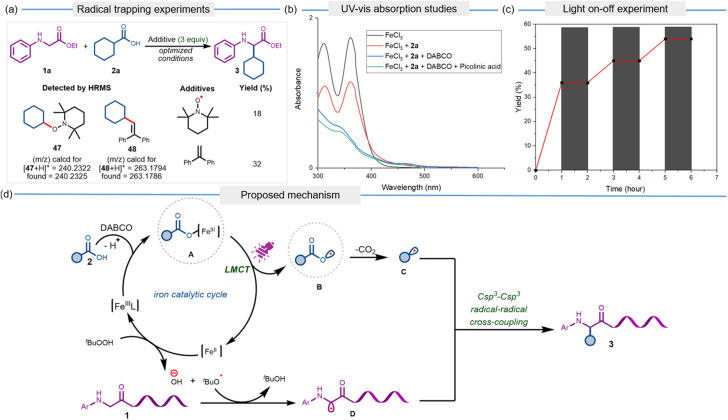
Mechanistic studies. (a) Radical trapping experiments. (b) UV-vis absorption studies. (c) Light on-off experiment. (d) Proposed mechanism.

To gain insight into the reaction mechanism, a series of control experiments was conducted. To probe the involvement of alkyl radicals in the iron-photocatalyzed alkylation protocol, the model reaction between 1a and 2a was carried out under standard conditions in the presence of radical scavengers, TEMPO, and ethene-1,1-diyldibenzene (Fig. 1a). Expectedly, the yield of desired product 3 plummeted under these conditions, and high-resolution mass spectrometry (HRMS) analysis confirmed the presence of radical-trapping adducts 47 and 48, providing strong evidence for the generation of alkyl radical intermediates during the transformation. UV-vis absorption spectra revealed a decrease in absorption between 300 and 400 nm upon addition of 2a, indicating a reduction in the concentration of Fe(iii) complex *via* ligand to metal charge transfer (Fig. 1b). The absorption spectrum was found to be further suppressed with the addition of DABCO and picolinic acid, accounting for the crucial roles of the base and additive in the reaction. To further probe the reaction mechanism, a ‘light on–off’ experiment was conducted by reacting 1a and 2a under the optimized reaction conditions, and it was observed that the formation of product 3 occurred exclusively under continuous light irradiation (Fig. 1c).

Based on our mechanistic studies, we propose that the reaction commenced with the formation of charge transfer complex A between FeCl_3_ and carboxylic acid 2*via* deprotonation in the presence of DABCO. Upon the irradiation of purple LED, an intramolecular ligand to metal charge transfer within complex A leads to the formation of carboxyl radical B and a reduced Fe(ii) species. The catalytic turnover is achieved through the oxidation of the Fe(ii) species by TBHP, leading to the regeneration of FeCl_3_ and *tert*-butoxy radical. While decarboxylation of B results in the generation of alkyl radical C, a hydrogen atom abstraction (HAT) from glycine derivative 1 by *tert*-butoxy radical furnishes persistent α-amino-carbon radical D.^66^ Finally, the persistent radical effect (PRE)-driven Csp^3^–Csp^3^ radical–radical cross-coupling between C and D delivers the desired product 3.

## Conclusion

In summary, we have developed a novel iron-photo-catalyzed LMCT-driven site-selective C(sp^3^)–H alkylation of glycine derivatives using feedstock carboxylic acids as alkylating agents. In general, a palette of alkyl carboxylic acids (primary, secondary, tertiary, α-heteroatom, and sterically encumbered) was cross-coupled with a variety of glycine derivatives to provide corresponding α-alkylated glycines in good yields under mild conditions. Notably, the methodology was further extended to the methylation of glycine derivatives using cost-effective, inexpensive, and readily available acetic acid as a methylating agent. The method was applicable to the site-selective alkylation of short and long-chain peptides. Importantly, the method broadened the scope for synthesizing peptide–drug conjugates by enabling the incorporation of structurally diverse drug molecules into the peptide framework selectively. Overall, the present methodology enables the synthesis of a wide range of alkylated glycine derivatives and peptides, thereby offering new opportunities for exploring the chemical space relevant to peptide-based biomolecules and drug discovery.

## Author contributions

Conceptualization: S. M. Experiments: S. P. P., M. S. P., and P. M. Data analysis: all authors. Writing of the original draft: all authors. Writing, review, and editing: S. M. and O. R. All authors have approved the final version of the manuscript.

## Conflicts of interest

There are no conflicts to declare.

## Supplementary Material

SC-016-D5SC07730C-s001

## Data Availability

The data that support the findings of this study are available in the supplementary material (SI) of this article. Supplementary information: the contains experimental details, additional optimization studies, pictures of the photochemical setup, details of mechanistic studies, and copies of ^1^H and ^13^C NMR spectra of all compounds. See DOI: https://doi.org/10.1039/d5sc07730c.
