# Supplementary material for: Iron photocatalysis towards site-selective C(sp3)–H alkylation of glycines and peptides
Source: Chem Sci. 2025 Oct 22;16(46):22084–90. doi: 10.1039/d5sc07730c (PMC12541469; doi:10.1039/d5sc07730c)

Supplementary Information (SI) for Chemical Science.
This journal is © The Royal Society of Chemistry 2025

Supporting Information

Iron-Photocatalysis Towards Site-Selective C(sp^3)-H Alkylation of Glycines and Peptides

Satya Prakash Panda,[a] M. Siva Prasad,[a] Prahallad Meher,[a] Oliver Reiser,*[b] and Sandip Murarka*[a]

[a]Department of Chemistry, Indian Institute of Technology Jodhpur, Karwar-342037, Rajasthan, India. Email: sandipmurarka@iitj.ac.in

[b]Department of Organic Chemistry, University of Regensburg, Universitätsstr. 31, 93053 Regensburg, Germany. Email: oliver.reiser@chemie.uni-regensburg.de

Table of Contents

1. Detailed Optimizations S2
2. General information S3
3. Preparation of starting materials S4
4. General experimental procedure S5
5. Gram Scale Synthesis S5
6. Image of setup for photoinduced reaction set up S6
7. Radical Trapping S7
8. UV-Visible absorption spectra S7
9. Light on-off experiment S8
10. Determination of Quantum Yield S9
11. Report of NMR spectra S12
12. References S29
13. NMR Spectra of compounds S30

1. Detailed Optimizations-:

Table-S1 Optimization Table for Iron-Photocatalyzed Site-selective Alkylation of Glycines and Peptides

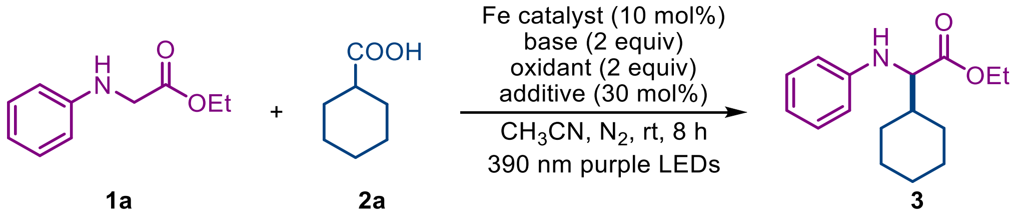

S. No.	Fe Catalyst	Base	oxidant	additive	Yield (%)[b]
1	$FeCl_3$	DABCO	------	------	38
2	$FeBr_3$	DABCO	------	------	28
3	$Fe(NO_3)_3.9H_2O$	DABCO	------	------	36
4	$FeCl_3$	Cs_2CO_3	------	------	26
5	$FeCl_3$	K_3PO_4	------	------	34
6	$FeCl_3$	Et_3N	------	------	Trace
7	$FeCl_3$	DBU	------	------	N.D.
8	$FeCl_3$	DMAP	------	------	23
9	$FeCl_3$	DABCO	$KBrO_3$	------	61
10	$FeCl_3$	DABCO	$NaIO_4$	------	54
11	$FeCl_3$	DABCO	$NaBrO_3$	------	63
12	$FeCl_3$	DABCO	TBHP	------	67
13	$FeCl_3$	DABCO	TBHP	Tetrabutylammonium bromide	38
14	$FeCl_3$	DABCO	TBHP	PPh_3	44
**15**	**$FeCl_3$**	**DABCO**	**TBHP**	**picolinic acid**	**76**
16[c]	$FeCl_3$	DABCO	TBHP	picolinic acid	64
17[d]	$FeCl_3$	DABCO	TBHP	picolinic acid	32
18[e]	$FeCl_3$	DABCO	TBHP	picolinic acid	22
19[f]	$FeCl_3$	DABCO	TBHP	picolinic acid	74
20[g]	$FeCl_3$	DABCO	TBHP	picolinic acid	61
21[h]	$FeCl_3$	DABCO	TBHP	picolinic acid	62
22[i]	$FeCl_3$	DABCO	TBHP	picolinic acid	53
23[j]	$FeCl_3$	DABCO	TBHP	picolinic acid	75
24[k]	$FeCl_3$	DABCO	TBHP	picolinic acid	42
25[l]	$FeCl_3$	DABCO	TBHP	picolinic acid	23
26[m]	$FeCl_3$	DABCO	TBHP	picolinic acid	N.D.
27	$FeCl_3$	-------	TBHP	picolinic acid	Trace
28	------	DABCO	TBHP	picolinic acid	N.D.

Reaction conditions[a]: **1a** (0.15 mmol, 1 equiv), **2a** (0.3 mmol, 2 equiv), Fe catalyst (10 mol%), base (2 equiv), oxidant (2 equiv), additive (30 mol%), CH_3CN (1.5 mL), irradiation by 390 nm purple LEDs at room temperature for 8 h under N_2 atmosphere. [b]isolated yield. [c]DCM instead of CH_3CN. [d]THF instead of CH_3CN. [e]methanol instead of CH_3CN. [f]3 equiv of **2a**. [g]1.5 equiv of **2a**. [h]1.5 equiv of DABCO. [i]1.5 equiv of TBHP. [j]20 mol% of $FeCl_3$. [k]427 nm blue LEDs

instead of 390 nm purple LEDs. [k]23 W CFL light instead of 390 nm purple LEDs. [m]without irradiation. N.D. = not detected.

2. General Information

Photochemical reactions were performed under air atmosphere for all the reaction sets using pre-dried glassware. All the solvents were obtained from Merck (Emparta grade). The following starting materials and the reaction components such as, aniline derivatives, alkyl carboxylic acids, K_2CO_3, TfOH, HATU, HOBT, EDC, DCC, DMAP, Et_3N, 1,1-diphenylethylene, and TEMPO were obtained from commercial sources and used without further purification. Glycine, and peptide derivatives were synthesized by following the synthetic procedures mentioned below. Yields refer to isolated compounds, estimated to be >95% pure as determined by ^1H NMR and ^{13}C NMR. All optimized reactions were conducted under the photo irradiation using 40W Kessil PR160L (Linear Reflector)- 456 nm lamp (Avg. Intensity in 2×4 cm area = 137 mW/cm^2) with 2 cm from the reaction tube made up of borosilicate glass without any filter. Thin layer chromatography (TLC) was performed on Merck pre-coated silica gel 60 F254 aluminum sheets with detection under UV light at 254 nm. Chromatographic separations were carried out on Avra silica gel (100-200 mesh or 230−400 mesh). Nuclear magnetic resonance (NMR) spectroscopy was performed using Bruker 500 MHz spectrometers. Chemical shifts (δ) are provided in ppm if not otherwise specified. HRMS spectra were recorded using Agilent 6500 Q-TOF spectrometer. UV-Vis experiments were performed on LABINDIA ANALYTICAL-2000 U UV/VIS Spectrophotometer instrument.

3. Preparation of starting materials:

3.1. Preparation of Glycine Derivatives and Peptides:

The substrates of various glycine derivatives and peptides (**1a, 1b, 1c, 1d, 1e, 1f, 1g, 1h, 1i, 1j, 1k, 1l, 1m, 1n, 1o, 1p, 1q, 1r, 1s, 1t, 1u),**[1b-d, 2a-c, 3, 4] were prepared following previous literature procedures and obtained characterization data were in alignment with the literature reported data.

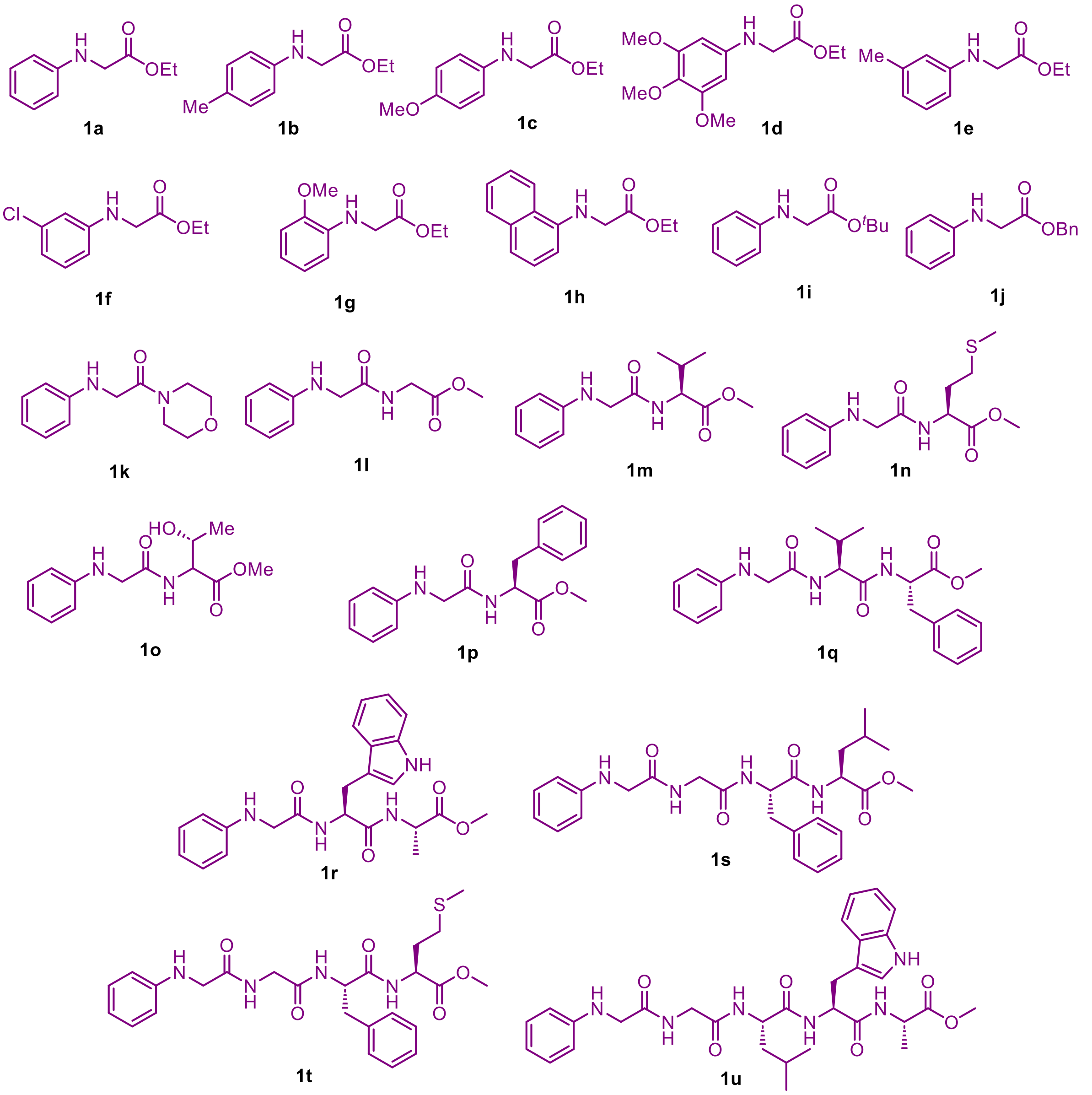

1a 1b 1c 1d 1e 1f 1g 1h 1i 1j 1k 1l 1m 1n 1o 1p 1q 1r 1s 1t 1u

Procedure for the synthesis of glycine derivatives and peptides:

Aniline (1.0 equiv) and NaOAc (1.1 equiv) were suspended in EtOH. Then, ethyl bromoacetate (1.0 eq.) was added and the mixture was stirred at reflux for 12 h under argon atmosphere. EtOH was removed in vacuo and the residue was partitioned between CH_2Cl_2 and NaCl aquous solution. The aqueous layer was extracted with CH_2Cl_2 (3×) and the combined organic layers

were dried with Na_2SO_4, filtered and evaporated. Column chromatography (silica) furnished glycine derivatives (**1a** to **1h**).

Other glycine derivatives, dipeptides, and polypeptides were prepared by following the reported literature procedures.[1-4]

4. General Procedure (GP) for Iron-Photoredox-Catalyzed Site-selective Alkylation of Glycines and Peptides:

Glycine or peptide derivatives **1** (0.25 mmol, 1 equiv), alkyl carboxylic acids **2** (0.5 mmol, 2 equiv), $FeCl_3$ (0.025 mmol, 10 mol%), DABCO (0.5 mmol, 2 equiv) and picolinic acid (0.075 mmol, 30 mol%) were added in a pre-dried 10 ml Schlenk tube under nitrogen atmosphere. The tube was degassed and purged with N_2 three times. Then, acetonitrile (2.5 mL), and TBHP (0.5 mmol, 2 equiv) were added under nitrogen, and the mixture was allowed to stir for 8 h under irradiation of 40 W Kessil purple LED (390 nm). After completion, the reaction mixture was concentrated under vacuum and purified by silica gel column chromatography using 0-40% ethyl acetate in hexane or 0-30% acetone in hexane to afford the corresponding alkylated glycine or peptide derivatives (**3-46**).

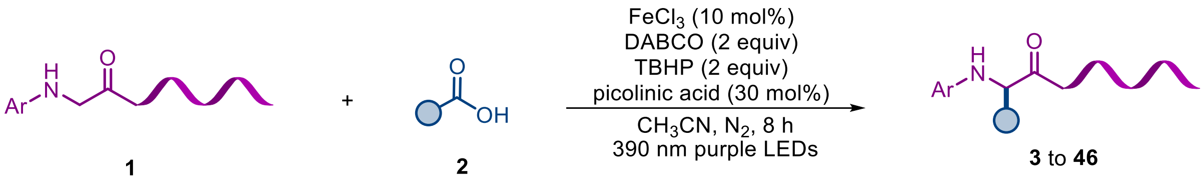

1 **2** **3** to **46**

5. Gram-Scale Synthesis:

Ethyl phenylglycinate **1a** (5.58 mmol, 1 equiv, 1 g), cyclohexanecarboxylic acid **2a** (11.16 mmol, 2 equiv), $FeCl_3$ (0.025 mmol, 10 mol%), DABCO (0.5 mmol, 2 equiv) and picolinic acid (0.075 mmol, 30 mol%) were added in a pre-dried 10 ml Schlenk tube under nitrogen atmosphere. The tube was degassed and purged with N_2 three times. Then, acetonitrile (2.5 mL), and TBHP (0.5 mmol, 2 equiv) were added under nitrogen, and the mixture was allowed to stir for 8 h under irradiation of 40 W Kessil purple LED (390 nm). After completion, the reaction mixture was concentrated under vacuum and purified by silica gel column chromatography using 0-5% ethyl acetate in hexane to afford the final product ethyl 2-cyclohexyl-2-(phenylamino)acetate **3** (0.920 g, 63%).

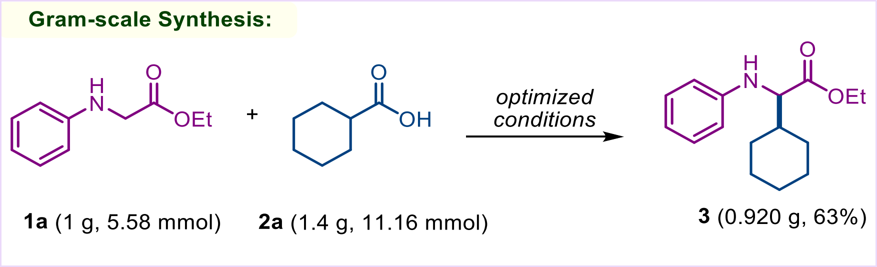

6. Image of the photoinduced reaction set up:

Front view:

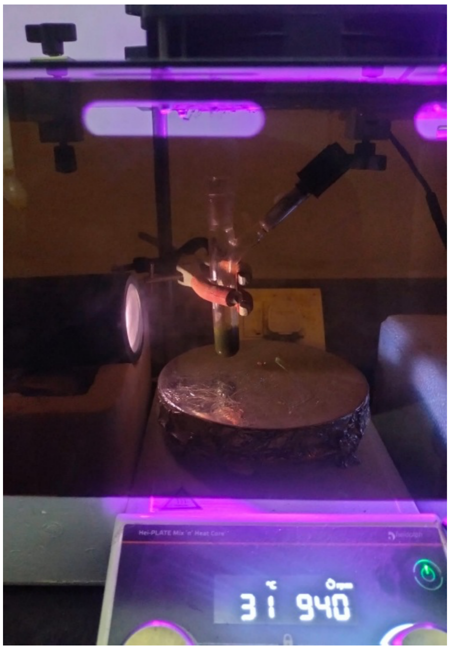

Top view:

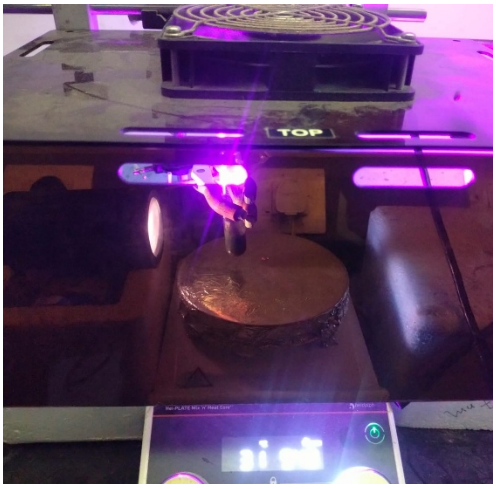

Figure S1: Representative pictures of reaction setup.

7. Procedure for the Radical Trapping Experiments:

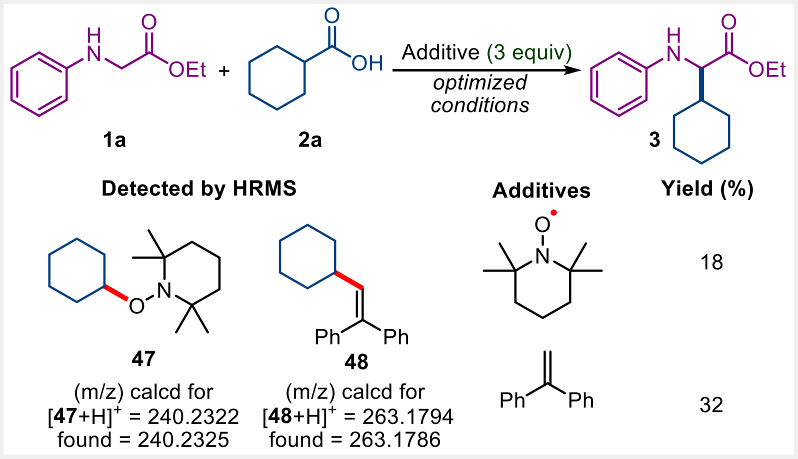

a. Ethyl phenylglycinate **1a** (0.15 mmol, 1 equiv), cyclohexanecarboxylic acid **2a** (0.3 mmol, 2 equiv), $FeCl_3$ (10 mol%), DABCO (0.3 mmol, 2 equiv), picolinic acid (30 mol%) and 2,2,6,6-Tetramethylpiperidine 1-oxyl (TEMPO, 0.45 mmol, 3.0 equiv) were added in a pre-dried 10 ml Schlenk tube under nitrogen atmosphere. The tube was degassed and purged with N_2 three times. Then, acetonitrile (2.5 mL) and TBHP (0.3 mmol, 2 equiv) were added under nitrogen, and the mixture was allowed to stir for 8 h under irradiation of 40 W Kessil purple LED (390 nm). After completion, the reaction mixture was concentrated under vacuum, and formation of adduct **47** was confirmed by HRMS.

b. Ethyl phenylglycinate **1a** (0.15 mmol, 1 equiv), cyclohexanecarboxylic acid **2a** (0.3 mmol, 2 equiv), $FeCl_3$ (10 mol%), DABCO (0.3 mmol, 2 equiv), picolinic acid (30 mol%) and were added in a pre-dried 10 ml Schlenk tube under nitrogen atmosphere. The tube was degassed and purged with N_2 three times. Then, acetonitrile (2.5 mL), TBHP (0.3 mmol, 2 equiv), and 1,1-diphenylethylene (0.45 mmol, 3.0 equiv) were added under nitrogen, and the mixture was allowed to stir for 8 h under irradiation of 40 W Kessil purple LED (390 nm). After completion, the reaction mixture was concentrated under vacuum, and formation of adduct **48** was confirmed by HRMS.

8. UV-Visible Absorption Spectra:

A 10 mL stock solution of each $FeCl_3$ (0.1 mM), cyclohexanecarboxylic acid (0.1 mM), DABCO (0.1 mM) and picolinic acid (0.1 mM) were prepared separately in CH_3CN. At first, the UV absorption of $FeCl_3$ was taken (Figure S2). Then, the mixture of $FeCl_3$ (0.1 mM) with cyclohexanecarboxylic acid (0.1 mM) were taken together in different 3 mL UV cuvette for UV-Vis absorption study under 390 nm purple LED irradiation. Then, $FeCl_3$ (0.1 mM),

cyclohexanecarboxylic acid (0.1 mM) and DABCO (0.1 mM) were taken together for the measurement of UV absorption spectra. At last, $FeCl_3$ (0.1 mM), cyclohexanecarboxylic acid (0.1 mM), DABCO (0.1 mM) and picolinic acid (0.1 mM) were taken for UV-Vis absorption study (Figure S2).

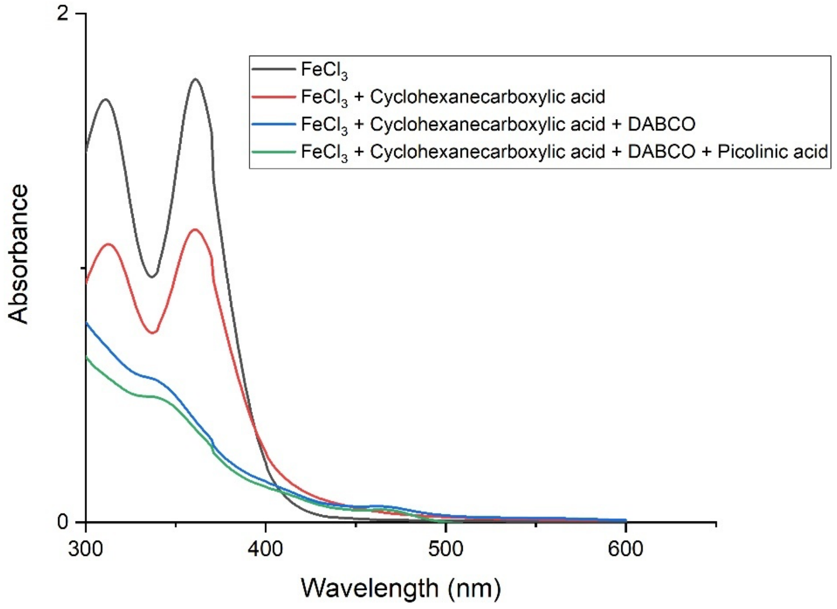

Figure S2: UV-Vis absorption spectra of $FeCl_3$ (0.1 mM), cyclohexanecarboxylic acid (0.1 mM), DABCO (0.1 mM), picolinic acid (0.1 mM) in CH_3CN.

Here, the graph indicates a gradual decrease in the intensity of $FeCl_3$ upon the addition of other components, suggesting the formation of an Fe(III) complex with cyclohexanecarboxylic acid **2a** in the presence of DABCO and picolinic acid. This complex subsequently undergoes a ligand-to-metal charge transfer (LMCT) process, leading to the generation of Fe(II) species and the corresponding alkyl radical.

9. Light On-Off experiment:

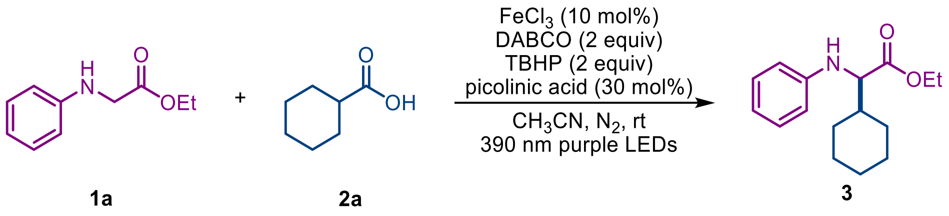

Ethyl phenylglycinate **1a** (0.3 mmol, 1 equiv), cyclohexanecarboxylic acid **2a** (0.6 mmol, 2 equiv), $FeCl_3$ (15 mol%), DABCO (2 equiv), and picolinic acid (30 mol%) were added in a

pre-dried 10 ml reaction tube under nitrogen atmosphere. The tube was degassed and purged with N_2 three times. Then, acetonitrile (2.5 mL) and TBHP (2 equiv) were added under nitrogen atmosphere, and the mixture was placed under irradiation of 40 W Kessil purple LED (390 nm) lamp where the reaction was placed in light and dark in every alternative 1 hour. After every time interval of 1 hour, a 0.5 mL reaction aliquot was taken out by a syringe and quenched with water, organic part was taken in DCM and NMR was carried out. The NMR yield was determined using mesitylene as an internal standard.

Entry	Time (hour)	Light source	Yield (%)
1	1	on	36
2	2	off	36
3	3	on	45
4	4	off	45
5	5	on	54
6	6	off	54

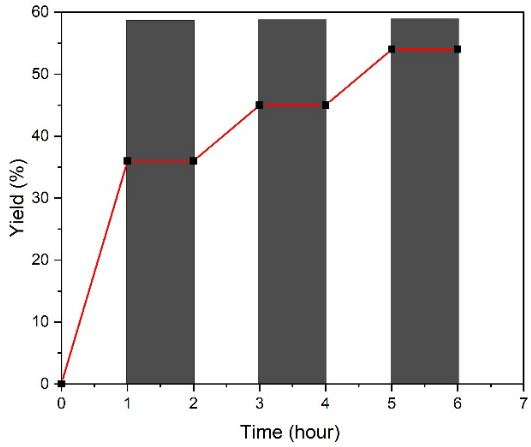

Figure S3: Light On-Off Experiment.

10. Determination of Quantum Yield:

A. Determination of light intensity of the Blue LED:

0.737 g of potassium ferrioxalate trihydrate was dissolved in 10 mL H_2SO_4 (0.05 M) and stored in the dark. Then, a buffer solution was prepared by dissolving 2.5 g of sodium acetate and 0.5 mL of H_2SO_4 (95-98%) in 50 mL of distilled water.

General Protocol to assess the photon flux of the 390 nm blue LEDs: To a 10 mL Schlenk flask containing a stirring bar, 1 mL of the actinometer solution was added. Then, the solution was irradiated for 60 s. Immediately, a 100 μL aliquot was taken and added to a 10 mL volumetric flask containing 15 mg of 1, 10-phenanthroline in 3 mL of the buffer solution. The flask was filled with distilled water. The absorbance of this solution was then measured at 510

nm by UV/Vis spectrophotometry. In a similar manner, this procedure is repeated with the actinometer solution stored in the dark. Using then the Beer's Law, the number of moles of Fe^{2+} produced by light irradiation is obtained by:

$$Fe^{2+} = \frac{v_1 v_3 \Delta A(510\text{ nm})}{10^3 v_2 l}$$

Where:

v_1 = Irradiated volume (1 mL)

v_2 = The aliquot of the irradiated solution taken for the estimation of Fe^{+} ions (0.100 mL)

v_3 = Final volume of the solution after complexation with 1, 10-phenanthroline (10 mL).

ε (510 nm) = Molar extinction coefficient of $[Fe\ (Phen)_3]^{2+}$ complex (11100 L mol^{-1} cm^{-1}).

l = Optical path-length of the cuvette (1 cm)

ΔA (510 nm) = absorbance difference between the irradiated solution and the solution stored in dark.

$$Fe^{2+} = \frac{1\text{ mL} \times 10\text{ mL} \times 0.5309\ (510\text{ nm})}{10^3 \times 0.1\text{ mL} \times 1\text{cm} \times 11100\text{ L mol}^{-1}\text{ cm}^{-1}}$$
$$= 4.78 \times 10^{-9}\text{ mol}$$

The photon flux (F) is obtained by using the following equation:

$$\phi(\lambda) = \frac{mol\ Fe^{2+}}{F(1\text{-}10^{-A(\lambda)})}$$

$$F = 4.12 \times 10^{-9}\text{ einsteins/s}$$

Where: Φ (λ) = The quantum yield for Fe^{2+} formation at 390 nm is 1.16.

A (λ) = ferrioxalate actinometer absorbance at 390 nm, which was measured placing 1 mL of the solution in a cuvette of path length 1 cm by UV/Vis spectrophotometry. We obtained an absorbance value of 3.512862. The photon flux (F) is 4.12 x 10^{-9} einstein/s.

B. Quantum Yield Calculation:

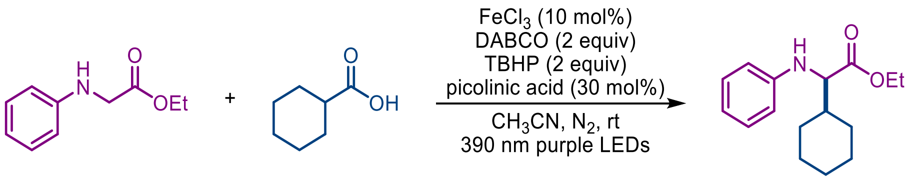

1a **2a** **3**

Ethyl phenylglycinate **1a** (0.15 mmol, 1 equiv), cyclohexanecarboxylic acid **2a** (0.3 mmol, 2 equiv), $FeCl_3$ (15 mol%), DABCO (2 equiv), picolinic acid (30 mol%) were added in a pre-dried 10 ml Schlenk tube under nitrogen atmosphere. The tube was degassed and purged with N_2 three times. Then, acetonitrile (2.5 mL) and TBHP (2 equiv) were added under nitrogen atmosphere. The mixture was irradiated using a 40 W Kessil purple LED (390 nm) in the optimized condition for 3600 s and $1.8x10^{-5}$ moles of product were obtained. The quantum yield was calculated using the following equation.

$$\phi\ (390\ \text{nm}) = \frac{mol\ of\ product}{\text{F}(1\text{-}10^{-A(390\ \text{nm})})t}$$

$$\phi\ (390\ \text{nm}) = \frac{1.8 \times 10^{-5}\ \text{mol}}{4.12 \times 10^{-9} \times 0.9996 \times 3600\ \text{s}}$$
$$= 1.214$$

Where: A (390 nm) = is the absorbance at 390 nm of the photocatalytic reaction which was measured placing 1 mL of the solution in a cuvette of path length 1 cm by UV/Vis spectrophotometry.

t = is the reaction time i.e., 3600s.

The quantum yield (Φ) of the reaction is **1.214**.

11. Report of NMR Spectra:

Ethyl 2-cyclohexyl-2-(phenylamino)acetate (3)[2c]

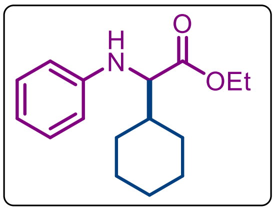

The compound was prepared according to GP using ethyl phenylglycinate (0.045 g, 0.25 mmol), cyclohexanecarboxylic acid (0.064 g, 0.50 mmol, 2 equiv). After 8 h, purification by column chromatography (0-5% ethyl acetate in hexane) gave **3** as a white solid (0.050 g, 76%).

^{1}H NMR (500 MHz, $CDCl_3$) δ 7.16 (t, J = 7.9 Hz, 2H), 6.72 (t, J = 7.3 Hz, 1H), 6.64 (d, J = 7.9 Hz, 2H), 4.17 (q, J = 7.1 Hz, 2H), 3.87 (d, J = 6.1 Hz, 1H), 1.88 – 1.67 (m, 6H), 1.27 – 1.16 (m, 8H). **^{13}C{^{1}H} NMR** (126 MHz, $CDCl_3$) δ 173.7, 147.4, 129.3, 118.2, 113.6, 62.1, 60.8, 41.3, 29.6, 29.2, 26.2, 26.09, 26.05, 14.3.

Ethyl 2-cyclohexyl-2-((4-methoxyphenyl)amino)acetate (4)[2c]

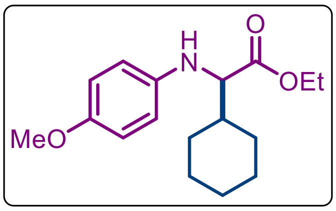

The compound was prepared according to GP using ethyl (4-methoxyphenyl)glycinate (0.052 g, 0.25 mmol), cyclohexanecarboxylic acid (0.064 g, 0.50 mmol, 2 equiv). After 8 h, purification by column chromatography (0-10% ethyl acetate in hexane) gave **4** as a viscous liquid (0.060 g, 82%).

^{1}H NMR (400 MHz, $CDCl_3$) δ 6.75 (d, J = 8.8 Hz, 2H), 6.62 (d, J = 8.8 Hz, 2H), 4.15 (q, J = 7.1 Hz, 2H), 3.76 (d, J = 6.1 Hz, 1H), 3.73 (s, 3H), 1.87 – 1.66 (m, 6H), 1.27 – 1.13 (m, 8H). **^{13}C{^{1}H} NMR** (101 MHz, $CDCl_3$) δ 173.9, 152.8, 141.4, 115.4, 114.9, 63.5, 60.7, 55.7, 41.3, 29.7, 29.3, 26.2, 26.11, 26.08, 14.3.

Ethyl 2-cyclohexyl-2-((3,4,5-trimethoxyphenyl)amino)acetate (5)[4a]

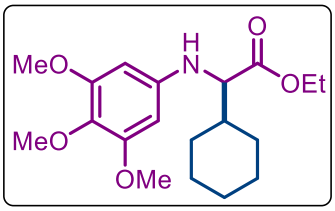

The compound was prepared according to GP using ethyl (3,4,5-trimethoxyphenyl)glycinate (0.069 g, 0.25 mmol), cyclohexanecarboxylic acid (0.064 g, 0.50 mmol, 2 equiv). After 8 h, purification by column chromatography (0-10% ethyl acetate in hexane) gave **5** as a white solid (0.064 g, 73%).

^{1}H NMR (500 MHz, $CDCl_3$) δ 5.88 (s, 2H), 4.18 (q, J = 7.1 Hz, 2H), 3.80 (s, 6H), 3.78 (d, J = 6.4 Hz, 1H), 3.74 (s, 3H), 1.89 – 1.67 (m, 6H), 1.27 – 1.15 (m, 8H). **^{13}C{^{1}H} NMR** (126 MHz, $CDCl_3$) δ 173.8, 153.9, 144.0, 130.7, 91.3, 62.7, 61.1, 60.9, 55.9, 41.3, 29.7, 29.3, 26.2, 26.1, 26.0, 14.4.

Ethyl 2-cyclohexyl-2-(*m*-tolylamino)acetate (6)[2a]

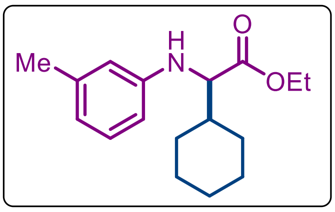

The compound was prepared according to GP using ethyl *m*-tolylglycinate (0.048 g, 0.25 mmol), cyclohexanecarboxylic acid (0.064 g, 0.50 mmol, 2 equiv). After 8 h, purification by column chromatography (0-5% ethyl acetate in hexane) gave **6** as a white solid (0.054 g, 78%).

^{1}H NMR (400 MHz, $CDCl_3$) δ 7.05 (t, *J* = 7.7 Hz, 1H), 6.55 (d, *J* = 7.4 Hz, 1H), 6.49 – 6.41 (m, 2H), 4.18 (q, *J* = 6.9 Hz, 2H), 3.86 (d, *J* = 6.1 Hz, 1H), 2.27 (s, 3H), 1.88 – 1.65 (m, 6H), 1.29 – 1.14 (m, 8H). **^{13}C{^{1}H} NMR** (101 MHz, $CDCl_3$) δ 173.8, 147.5, 139.1, 129.2, 119.1, 114.4, 110.6, 62.1, 60.8, 41.4, 29.6, 29.2, 26.2, 26.12, 26.08, 21.6, 14.3.

Ethyl 2-((3-chlorophenyl)amino)-2-cyclohexylacetate (7)[2a]

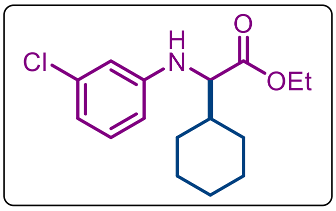

The compound was prepared according to GP using ethyl (3-chlorophenyl)glycinate (0.053 g, 0.25 mmol), cyclohexanecarboxylic acid (0.064 g, 0.50 mmol, 2 equiv). After 8 h, purification by column chromatography (0-5% ethyl acetate in hexane) gave **7** as a white solid (0.045 g, 61%).

^{1}H NMR (400 MHz, $CDCl_3$) δ 7.05 (t, *J* = 8.0 Hz, 1H), 6.67 (d, *J* = 7.8 Hz, 1H), 6.60 – 6.59 (m, 1H), 6.49 (dd, *J* = 8.2, 1.8 Hz, 1H), 4.18 (q, *J* = 6.8 Hz, 2H), 3.82 (d, *J* = 6.0 Hz, 1H), 1.83 – 1.66 (m, 6H), 1.29 – 1.13 (m, 8H). **^{13}C{^{1}H} NMR** (101 MHz, $CDCl_3$) δ 173.2, 148.6, 135.0, 130.2, 118.0, 113.2, 111.8, 61.8, 61.0, 41.3, 29.6, 29.1, 26.14, 26.05, 26.0, 14.3.

Ethyl 2-cyclohexyl-2-((2-methoxyphenyl)amino)acetate (8)

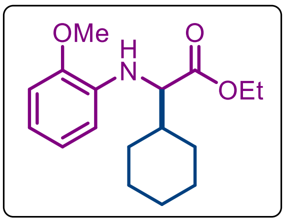

The compound was prepared according to GP using ethyl (2-methoxyphenyl)glycinate (0.052 g, 0.25 mmol), cyclohexanecarboxylic acid (0.064 g, 0.50 mmol, 2 equiv). After 8 h, purification by column chromatography (0-10% ethyl acetate in hexane) gave **8** as a white solid (0.042 g, 58%).

^{1}H NMR (500 MHz, $CDCl_3$) δ 6.83 – 6.80 (m, 1H), 6.78 – 6.75 (m, 1H), 6.68 – 6.65 (m, 1H), 6.56 – 6.54 (m, 1H), 4.16 (q, *J* = 7.1 Hz, 2H), 3.87 – 3.84 (m, 4H), 1.91 – 1.66 (m, 6H), 1.31 – 1.15 (m, 8H). **^{13}C{^{1}H} NMR** (126 MHz, $CDCl_3$) δ 173.7, 147.1, 137.3, 121.1, 117.1, 110.2, 109.8, 61.8, 60.7, 55.5, 41.2, 29.6, 29.3, 26.2, 26.10, 26.07, 14.3. **HRMS-ESI** (m/z): calcd for $C_{17}H_{26}NO_3$ $[M+H]^+$ 292.1907; found 292.1909.

Ethyl 2-cyclohexyl-2-(naphthalen-1-ylamino)acetate (9)[1d]

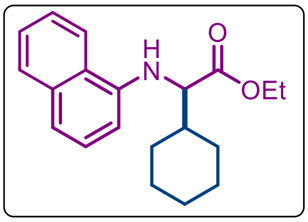

The compound was prepared according to GP using ethyl naphthalen-1-ylglycinate (0.057 g, 0.25 mmol), cyclohexanecarboxylic acid (0.064 g, 0.50 mmol, 2 equiv). After 8 h, purification by column chromatography (0-10% ethyl acetate in hexane) gave **9** as a white solid (0.048 g, 62%).

^{1}H NMR (500 MHz, $CDCl_3$) δ 7.97 – 7.92 (m, 1H), 7.83 – 7.78 (m, 1H), 7.51 – 7.46 (m, 2H), 7.33 (t, J = 7.8 Hz, 1H), 7.27 (d, J = 8.3 Hz, 1H), 6.60 (d, J = 7.3 Hz, 1H), 4.25 – 4.19 (m, 2H), 4.08 (d, J = 6.1 Hz, 1H), 2.01 – 1.73 (m, 6H), 1.34 – 1.25 (m, 8H). **^{13}C{^{1}H} NMR** (126 MHz, $CDCl_3$) δ 173.8, 142.6, 134.4, 128.6, 126.4, 125.8, 124.8, 123.8, 120.1, 118.1, 105.2, 62.0, 60.9, 41.4, 29.7, 29.5, 26.23, 26.17, 26.1, 14.3.

***Tert*-butyl 2-cyclohexyl-2-(phenylamino)acetate (10)**[2c]

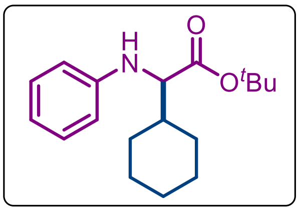

The compound was prepared according to GP using *tert*-butyl phenylglycinate (0.052 g, 0.25 mmol), cyclohexanecarboxylic acid (0.064 g, 0.50 mmol, 2 equiv). After 8 h, purification by column chromatography (0-5% ethyl acetate in hexane) gave **10** as a white solid (0.054 g, 75%).

^{1}H NMR (500 MHz, $CDCl_3$) δ 7.16 (t, J = 7.5 Hz, 2H), 6.71 (t, J = 7.2 Hz, 1H), 6.63 (d, J = 7.7 Hz, 2H), 3.76 (d, J = 5.4 Hz, 1H), 1.85 – 1.67 (m, 6H), 1.43 (s, 9H), 1.26 – 1.16 (m, 5H). **^{13}C{^{1}H} NMR** (126 MHz, $CDCl_3$) δ 172.9, 147.7, 129.2, 117.9, 113.6, 81.4, 62.5, 41.3, 29.6, 29.2, 28.1, 26.3, 26.2, 26.1.

2-Cyclohexyl-1-morpholino-2-(phenylamino)ethan-1-one (11)[4c]

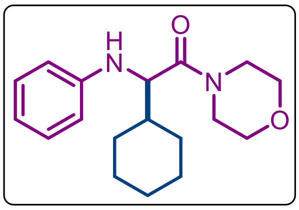

The compound was prepared according to GP using 1-morpholino-2-(phenylamino)ethan-1-one (0.055 g, 0.25 mmol), cyclohexanecarboxylic acid (0.064 g, 0.50 mmol, 2 equiv). After 8 h, purification by column chromatography (0-10% ethyl acetate in hexane) gave **11** as a white solid (0.039 g, 52%).

^{1}H NMR (500 MHz, $CDCl_3$) δ 7.16 (t, J = 7.8 Hz, 2H), 6.74 (t, J = 7.3 Hz, 1H), 6.66 (d, J = 8.0 Hz, 2H), 4.11 (d, J = 5.9 Hz, 1H), 3.69 – 3.55 (m, 8H), 1.89 – 1.68 (m, 6H), 1.26 – 1.13 (m, 5H). **^{13}C{^{1}H} NMR** (126 MHz, $CDCl_3$) δ 171.6, 147.6, 129.3, 118.6, 114.4, 67.0, 66.6, 58.7, 46.3, 42.5, 41.9, 30.2, 28.8, 26.2, 26.1.

Ethyl 2-(phenylamino)heptanoate (12)[4b]

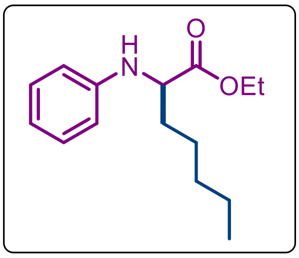

The compound was prepared according to GP using ethyl phenylglycinate (0.045 g, 0.25 mmol), hexanoic acid (0.058 g, 0.50 mmol, 2 equiv). After 8 h, purification by column chromatography (0-5% ethyl acetate in hexane) gave **12** as a white solid (0.042 g, 67%).

^{1}H NMR (500 MHz, $CDCl_3$) δ 7.18 – 7.15 (m, 2H), 6.73 (t, *J* = 7.3 Hz, 1H), 6.63 (d, *J* = 7.9 Hz, 2H), 4.18 (q, *J* = 7.1 Hz, 2H), 4.04 (t, *J* = 6.4 Hz, 1H), 1.86 – 1.72 (m, 2H), 1.47 – 1.38 (m, 2H), 1.33 – 1.28 (m, 4H), 1.24 (t, *J* = 7.1 Hz, 3H), 0.89 (t, *J* = 6.9 Hz, 3H). **^{13}C{^{1}H} NMR** (126 MHz, $CDCl_3$) δ 174.3, 146.9, 129.3, 118.3, 113.5, 61.0, 56.7, 33.0, 31.5, 25.2, 22.4, 14.2, 14.0.

Ethyl 2-(phenylamino)hex-5-enoate (13)[4a]

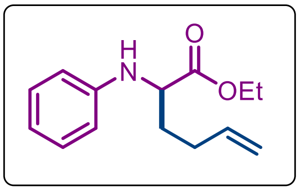

The compound was prepared according to GP using ethyl phenylglycinate (0.045 g, 0.25 mmol), pent-4-enoic acid (0.050 g, 0.50 mmol, 2 equiv). After 8 h, purification by column chromatography (0-5% ethyl acetate in hexane) gave **13** as a white solid (0.030 g, 51%).

^{1}H NMR (400 MHz, $CDCl_3$) δ 7.10 (t, *J* = 7.8 Hz, 2H), 6.67 (t, *J* = 7.3 Hz, 1H), 6.56 (d, *J* = 8.1 Hz, 2H), 5.80 – 5.69 (m, 1H), 5.01 – 4.93 (m, 2H), 4.13 – 4.08 (m, 2H), 4.00 (t, *J* = 6.4 Hz, 1H), 2.16 – 2.11 (m, 2H), 1.91 – 1.74 (m, 2H), 1.17 (t, *J* = 7.1 Hz, 3H). **^{13}C{^{1}H} NMR** (101 MHz, $CDCl_3$) δ 174.0, 146.8, 137.2, 129.3, 118.5, 115.7, 113.7, 61.1, 56.2, 32.2, 29.7, 14.2.

Ethyl 5-phenyl-2-(phenylamino)pentanoate (14)

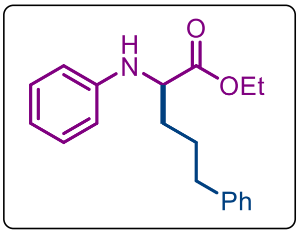

The compound was prepared according to GP using ethyl phenylglycinate (0.045 g, 0.25 mmol), 4-phenylbutanoic acid (0.082 g, 0.50 mmol, 2 equiv). After 8 h, purification by column chromatography (0-5% ethyl acetate in hexane) gave **14** as a white solid (0.048 g, 64%).

^{1}H NMR (500 MHz, $CDCl_3$) δ 7.32 – 7.28 (m, 2H), 7.24 – 7.17 (m, 5H), 6.77 (t, *J* = 7.3 Hz, 1H), 6.65 (d, *J* = 8.0 Hz, 2H), 4.20 (q, *J* = 7.1 Hz, 2H), 4.10 (t, *J* = 5.7 Hz, 1H), 2.73 – 2.64 (m, 2H), 1.98 – 1.88 (m, 1H), 1.86 – 1.77 (m, 3H), 1.26 (t, *J* = 7.1 Hz, 3H). **^{13}C{^{1}H} NMR** (126 MHz, $CDCl_3$) δ 174.1, 146.8, 141.7, 129.3, 128.38, 128.37, 125.9, 118.3, 113.5, 61.1, 56.6,

35.5, 32.5, 27.3, 14.2. **HRMS-ESI** (m/z): calcd for $C_{19}H_{24}NO_2$ $[M+H]^+$ 298.1802; found 298.1811.

Ethyl 3-ethyl-2-(phenylamino)heptanoate (15)[2c]

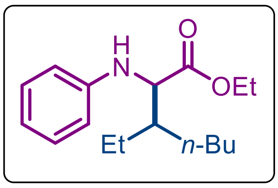

The compound was prepared according to GP using ethyl phenylglycinate (0.045 g, 0.25 mmol), 2-ethylhexanoic acid (0.072 g, 0.50 mmol, 2 equiv). After 8 h, purification by column chromatography (0-5% ethyl acetate in hexane) gave **15** as a white solid (0.051 g, 74%, dr = 1:1).

^{1}H NMR (500 MHz, $CDCl_3$) δ 7.17 (t, J = 7.8 Hz, 2H), 6.73 (t, J = 7.2 Hz, 1H), 6.65 (d, J = 7.2 Hz, 2H), 4.20 – 4.13 (m, 2H), 4.09 – 4.06 (m, 1H), 1.79 – 1.76 (m, 1H), 1.53 – 1.43 (m, 2H), 1.38 – 1.28 (m, 6H), 1.24 (t, J = 7.1 Hz, 3H), 0.97 – 0.93 (m, 3H), 0.92 – 0.88 (m, 3H). **^{13}C{^{1}H} NMR** (126 MHz, $CDCl_3$) δ 173.9, 147.2, 129.3, 118.4, 118.3, 113.8, 113.7, 60.9, 60.8, 59.0, 42.7, 29.5, 29.4, 29.3, 29.2, 23.1, 22.93, 22.90, 22.6, 14.3, 14.1, 14.0, 11.6, 11.5.

***Tert*-butyl 2-(2-ethoxy-2-oxo-1-(phenylamino)ethyl)pyrrolidine-1-carboxylate (16)**[4]

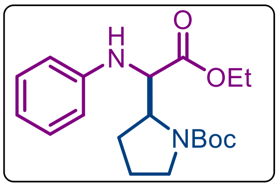

The compound was prepared according to GP using ethyl phenylglycinate (0.045 g, 0.25 mmol), (*tert*-butoxycarbonyl)proline (0.108 g, 0.50 mmol, 2 equiv). After 8 h, purification by column chromatography (10-20% ethyl acetate in hexane) gave **16** as a white solid (0.054 g, 62%, dr > 20:1).

^{1}H NMR (500 MHz, $CDCl_3$) δ 7.23 – 7.12 (m, 2H), 6.80 – 6.61 (m, 3H), 4.72 – 4.37 (m, 1H), 4.23 – 4.12 (m, 3H), 3.57 – 3.11 (m, 2H), 2.03 – 1.71 (m, 4H), 1.62 – 1.43 (m, 9H), 1.29 – 1.21 (m, 3H). **^{13}C{^{1}H} NMR** (126 MHz, $CDCl_3$) δ 171.0 (X 2C), 154.2, 153.9, 147.9, 147.12, 147.09, 129.3, 129.2, 118.3, 117.6, 117.5, 113.18, 113.16, 112.9, 112.5, 80.0, 79.9, 61.5, 61.1, 60.4, 59.6, 58.1, 57.7, 53.5, 47.3, 47.1, 28.7, 28.5, 27.3, 27.0, 24.3, 23.7, 14.2, 14.1.

***Tert*-butyl 4-(2-ethoxy-2-oxo-1-(phenylamino)ethyl)piperidine-1-carboxylate (17)**[2c]

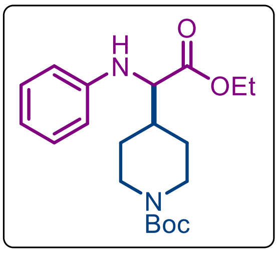

The compound was prepared according to GP using ethyl phenylglycinate (0.045 g, 0.25 mmol), 1-(*tert*-butoxycarbonyl)piperidine-4-carboxylic acid (0.115 g, 0.50 mmol, 2 equiv). After 8 h, purification by column chromatography (0-15% ethyl acetate in hexane) gave **17** as a white solid (0.051 g, 56%).

^{1}H NMR (500 MHz, $CDCl_3$) δ 7.16 (t, J = 7.9 Hz, 2H), 6.74 (t, J = 7.3 Hz, 1H), 6.63 (d, J = 8.3 Hz, 2H), 4.19 – 4.09 (m, 4H), 3.90 (d, J = 6.3 Hz, 1H), 2.74 – 2.60 (m, 2H), 1.94 – 1.78 (m, 2H), 1.63 (m, 1H), 1.44 (s, 9H), 1.40 – 1.32 (m, 2H), 1.24 (t, J = 6.8 Hz, 3H). **^{13}C{^{1}H} NMR** (126 MHz, $CDCl_3$) δ 173.1, 154.7, 147.0, 129.3, 118.6, 113.7, 79.5, 61.3, 61.1, 43.7, 39.7, 28.6, 28.4, 14.3.

Ethyl 2-(phenylamino)-2-(tetrahydro-2*H*-pyran-4-yl)acetate (18)[1b]

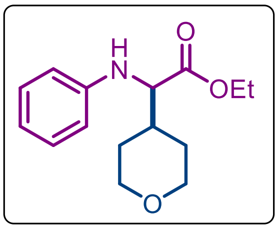

The compound was prepared according to GP using ethyl phenylglycinate (0.045 g, 0.25 mmol), tetrahydro-2*H*-pyran-4-carboxylic acid (0.065 g, 0.50 mmol, 2 equiv). After 8 h, purification by column chromatography (0-10% ethyl acetate in hexane) gave **18** as a white solid (0.040 g, 61%).

^{1}H NMR (500 MHz, $CDCl_3$) δ 7.17 (t, J = 7.9 Hz, 2H), 6.75 (t, J = 7.3 Hz, 1H), 6.64 (d, J = 7.9 Hz, 2H), 4.18 (q, J = 7.1 Hz, 2H), 4.05 – 3.98 (m, 2H), 3.91 (d, J = 6.6 Hz, 1H), 3.43 – 3.35 (m, 2H), 2.05 – 1.95 (m, 1H), 1.78 – 1.75 (m, 1H), 1.60 – 1.52 (m, 3H), 1.25 (t, J = 7.1 Hz, 3H). **^{13}C{^{1}H} NMR** (126 MHz, $CDCl_3$) δ 173.1, 147.1, 129.4, 118.5, 113.7, 67.8, 67.6, 61.5, 61.1, 38.7, 29.38, 29.35, 14.3.

Ethyl 3,3-dimethyl-2-(phenylamino)butanoate (19)[2c]

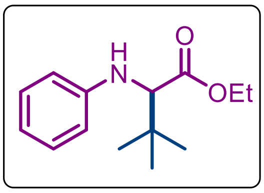

The compound was prepared according to GP using ethyl phenylglycinate (0.045 g, 0.25 mmol), pivalic acid (0.051 g, 0.50 mmol, 2 equiv). After 8 h, purification by column chromatography (0-5% ethyl acetate in hexane) gave **19** as a white solid (0.044 g, 74%).

^{1}H NMR (400 MHz, $CDCl_3$) δ 7.17 (t, J = 7.8 Hz, 2H), 6.73 (t, J = 7.3 Hz, 1H), 6.67 (d, J = 8.1 Hz, 2H), 4.17 – 4.12 (m, 2H), 3.79 (s, 1H), 1.24 (t, J = 7.3 Hz, 3H), 1.07 (s, 9H). **^{13}C{^{1}H} NMR** (101 MHz, $CDCl_3$) δ 173.4, 147.7, 129.3, 118.3, 113.9, 65.5, 60.6, 34.5, 26.8, 14.3.

Ethyl 3,3-dimethyl-2-(phenylamino)pentanoate (20)

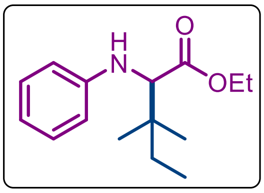

The compound was prepared according to GP using ethyl phenylglycinate (0.045 g, 0.25 mmol), 2,2-dimethylbutanoic acid (0.058 g, 0.50 mmol, 2 equiv). After 8 h, purification by column chromatography (0-5% ethyl acetate in hexane) gave **20** as a white solid (0.047 g, 76%).

^{1}H NMR (500 MHz, $CDCl_3$) δ 7.16 (t, *J* = 7.9 Hz, 2H), 6.73 (t, *J* = 7.3 Hz, 1H), 6.66 (d, *J* = 8.0 Hz, 2H), 4.17 – 4.11 (m, 2H), 3.87 (s, 1H), 1.49 – 1.40 (m, 2H), 1.23 (t, *J* = 7.1 Hz, 3H), 1.02 (s, 3H), 1.00 (s, 3H), 0.91 (t, *J* = 7.5 Hz, 3H). **^{13}C{^{1}H} NMR** (126 MHz, $CDCl_3$) δ 173.5, 147.7, 129.3, 118.3, 113.8, 63.8, 60.6, 37.1, 32.1, 23.4, 23.1, 14.3, 8.2. **HRMS-ESI** (m/z): calcd for $C_{15}H_{24}NO_2$ $[M+H]^+$ 250.1802; found 250.1808.

Methyl 3-(2-ethoxy-2-oxo-1-(phenylamino)ethyl)bicyclo[1.1.1]pentane-1-carboxylate (21)

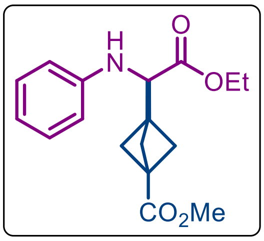

The compound was prepared according to GP using ethyl phenylglycinate (0.045 g, 0.25 mmol), 3-(methoxycarbonyl)-bicyclo[1.1.1]pentane-1-carboxylic acid (0.085 g, 0.50 mmol, 2 equiv). After 8 h, purification by column chromatography (0-5% ethyl acetate in hexane) gave **21** as a white solid (0.041 g, 54%).

^{1}H NMR (400 MHz, $CDCl_3$) δ 7.16 (t, *J* = 7.8 Hz, 2H), 6.74 (t, *J* = 7.3 Hz, 1H), 6.61 (d, *J* = 8.1 Hz, 2H), 4.25 – 4.16 (m, 3H), 3.67 (s, 3H), 2.06 (s, 6H), 1.27 (t, *J* = 7.1 Hz, 3H). **^{13}C{^{1}H} NMR** (101 MHz, $CDCl_3$) δ 171.4, 169.9, 146.6, 129.3, 118.6, 113.5, 61.3, 57.0, 52.4, 51.8, 50.8, 40.0, 37.2, 14.4. **HRMS-ESI** (m/z): calcd for $C_{17}H_{22}BrNO_4$ $[M+H]^+$ 304.1543; found 304.1549.

Ethyl 2-(1-methylcyclohexyl)-2-(phenylamino)acetate (22)[2c]

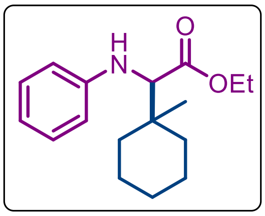

The compound was prepared according to GP using ethyl phenylglycinate (0.045 g, 0.25 mmol), 1-methylcyclohexane-1-carboxylic acid (0.071 g, 0.50 mmol, 2 equiv). After 8 h, purification by column chromatography (0-5% ethyl acetate in hexane) gave **22** as a white solid (0.054 g, 78%).

^{1}H NMR (500 MHz, $CDCl_3$) δ 7.16 (t, *J* = 7.9 Hz, 2H), 6.72 (t, *J* = 7.3 Hz, 1H), 6.67 (d, *J* = 7.9 Hz, 2H), 4.17 – 4.12 (m, 3H), 3.94 (s, 1H), 1.65 – 1.46 (m, 8H), 1.37 – 1.29 (m, 2H), 1.23 (t, J = 7.1 Hz, 3H), 1.05 (s, 3H). **^{13}C{^{1}H} NMR** (126 MHz, $CDCl_3$) δ 173.3, 147.8, 129.3, 118.2, 113.9, 64.5, 60.5, 37.2, 34.94, 34.91, 26.1, 21.8, 21.7, 20.4, 14.3.

Ethyl 2-((3r,5r,7r)-adamantan-1-yl)-2-(phenylamino)acetate (23)[2c]

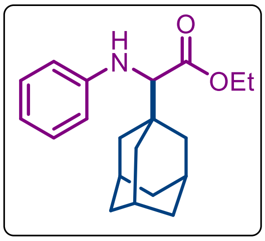

The compound was prepared according to GP using ethyl phenylglycinate (0.045 g, 0.25 mmol), (3r,5r,7r)-adamantane-1-carboxylic acid (0.090 g, 0.50 mmol, 2 equiv). After 8 h, purification by column chromatography (0-5% ethyl acetate in hexane) gave **23** as a white solid (0.053 g, 67%).

^{1}H NMR (500 MHz, $CDCl_3$) δ 7.16 (t, J = 7.9 Hz, 2H), 6.72 (t, J = 7.3 Hz, 1H), 6.66 (d, J = 8.0 Hz, 2H), 4.15 (q, J = 7.1 Hz, 2H), 3.66 (s, 1H), 2.03 (s, 3H), 1.82 – 1.58 (m, 12H), 1.23 (d, J = 7.1 Hz, 3H). **^{13}C{^{1}H} NMR** (126 MHz, $CDCl_3$) δ 172.8, 147.8, 129.3, 118.2, 113.8, 66.5, 60.5, 39.0, 36.9, 36.4, 28.4, 14.4.

In the similar manner we have carried out following quick optimization for the methylation process.

Table S2: Optimization table for methylation of glycine derivatives

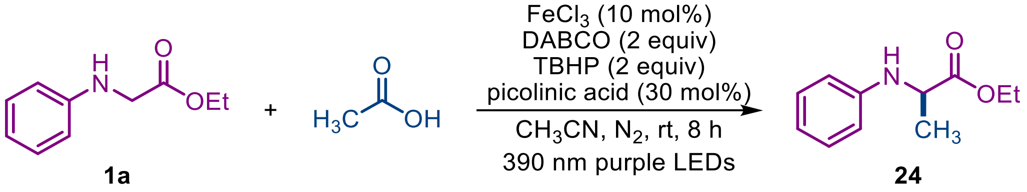

Sl No.	Catalyst	Base	Oxidant	Additive	Yield (%)[b]
1	$FeCl_3$	DABCO	TBHP	picolinic acid	42
2[c]	$FeCl_3$	DABCO	TBHP	picolinic acid	Trace
3[d]	$FeCl_3$	DABCO	TBHP	picolinic acid	22

Reaction conditions[a]: **1a** (0.15 mmol, 1 equiv), acetic acid (0.9 mmol, 6 equiv), $FeCl_3$ (10 mol%), DABCO (2 equiv), TBHP (2 equiv), picolinic acid (30 mol%), CH_3CN (1.5 mL), irradiation by 390 nm purple LEDs at room temperature for 8 h under N_2 atmosphere. [b]isolated yield, [c]in the absence of acetic acid, [d]2 equiv of acetic acid was used.

Ethyl phenylalaninate (24)[3]

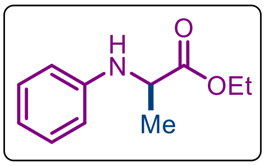

The compound was prepared according to GP using ethyl phenylglycinate (0.045 g, 0.25 mmol), acetic acid (0.090 g, 1.50 mmol, 6 equiv). After 8 h, purification by column chromatography (0-2% ethyl acetate in hexane) gave **24** as a viscous liquid (0.020 g, 42%).

^{1}H NMR (500 MHz, $CDCl_3$) δ 7.20 (t, *J* = 7.9 Hz, 2H), 6.81 (t, *J* = 7.3 Hz, 1H), 6.73 (d, *J* = 7.9 Hz, 2H), 4.20 – 4.12 (m, 3H), 1.50 (d, *J* = 7.0 Hz, 3H), 1.25 (t, *J* = 7.1 Hz, 3H). **^{13}C{^{1}H} NMR** (126 MHz, $CDCl_3$) δ 173.9, 145.2, 129.4, 119.5, 114.8, 61.4, 53.0, 18.6, 14.2.

Ethyl (4-methoxyphenyl)alaninate (25)[3]

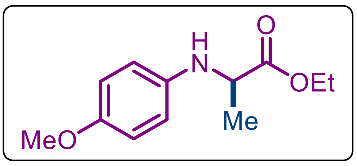

The compound was prepared according to GP using ethyl (4-methoxyphenyl)glycinate (0.052 g, 0.25 mmol), acetic acid (0.090 g, 1.50 mmol, 6 equiv). After 8 h, purification by column chromatography (0-2% ethyl acetate in hexane) gave **25** as a viscous liquid (0.026 g, 46%).

^{1}H NMR (500 MHz, $CDCl_3$) δ 6.74 (d, *J* = 8.9 Hz, 2H), 6.57 (d, *J* = 8.8 Hz, 2H), 4.14 (q, *J* = 7.1 Hz, 2H), 4.03 (q, *J* = 6.9 Hz, 1H), 3.87 (br s, 1H), 3.69 (s, 3H), 1.41 (d, *J* = 7.0 Hz, 3H), 1.20 (t, *J* = 7.1 Hz, 3H). **^{13}C{^{1}H} NMR** (126 MHz, $CDCl_3$) δ 174.9, 152.7, 140.9, 115.0, 114.8, 60.9, 55.5, 53.1, 18.9, 14.2.

Ethyl *m*-tolylalaninate (26)[3]

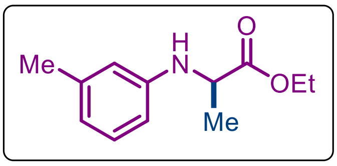

The compound was prepared according to GP using ethyl (4-methoxyphenyl)glycinate (0.052 g, 0.25 mmol), acetic acid (0.090 g, 1.50 mmol, 6 equiv). After 8 h, purification by column chromatography (0-2% ethyl acetate in hexane) gave **26** as a viscous liquid (0.023 g, 45%).

^{1}H NMR (500 MHz, $CDCl_3$) δ 7.11 (t, *J* = 7.7 Hz, 1H), 6.62 (d, *J* = 7.5 Hz, 1H), 6.52 – 6.46 (m, 2H), 4.28 – 4.21 (m, 2H), 4.18 (q, *J* = 6.9 Hz, 1H), 2.32 (s, 3H), 1.51 (d, *J* = 7.0 Hz, 3H), 1.31 (t, *J* = 7.1 Hz, 3H). **^{13}C{^{1}H} NMR** (126 MHz, $CDCl_3$) δ 174.7, 146.7, 139.0, 129.2, 119.2, 114.3, 110.5, 61.1, 52.1, 21.6, 19.0, 14.2.

Benzyl phenylalaninate (27)

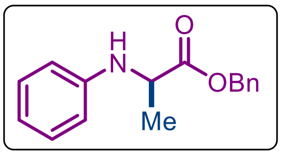

The compound was prepared according to GP using benzyl phenylglycinate (0.060 g, 0.25 mmol), acetic acid (0.090 g, 1.50 mmol, 6 equiv). After 8 h, purification by column chromatography (5-10% ethyl acetate in hexane) gave **27** as a white solid (0.033 g, 51%).

^{1}H NMR (500 MHz, $CDCl_3$) δ 7.63 (d, *J* = 7.8 Hz, 2H), 7.40 – 7.33 (m, 7H), 7.18 (t, *J* = 7.4 Hz, 1H), 5.22 (s, 2H), 4.68 – 4.62 (m, 1H), 1.52 (d, *J* = 7.2 Hz, 3H). **^{13}C{^{1}H} NMR** (126 MHz,

CDCl3) δ 171.4, 136.3, 135.1, 129.2, 128.7, 128.6, 128.2, 125.4, 119.8, 67.5, 48.8, 18.0. **HRMS-ESI** (m/z): calcd for $C_{16}H_{18}NO_2$ $[M+H]^+$ 256.1132; found 256.1135.

4-((1-Cyclohexyl-2-ethoxy-2-oxoethyl)amino)phenyl 2-(4-isobutylphenyl)propanoate (28)[2a]

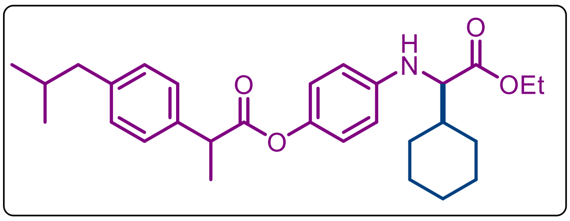

The compound was prepared according to GP using 4-((2-ethoxy-2-oxoethyl)amino)phenyl 2-(4-isobutylphenyl)propanoate (0.096 g, 0.25 mmol), cyclohexanecarboxylic acid (0.064 g, 0.50 mmol, 2 equiv). After 8 h, purification by column chromatography (0-5% ethyl acetate in hexane) gave **28** as a white solid (0.075 g, 64%).

^{1}H NMR (500 MHz, CDCl3) δ 7.28 (d, J = 8.0 Hz, 2H), 7.13 (d, J = 8.0 Hz, 2H), 6.81 – 6.77 (m, 2H), 6.58 – 6.53 (m, 2H), 4.18 – 4.13 (m, 2H), 3.89 (q, J = 7.1 Hz, 1H), 3.78 (d, J = 6.1 Hz, 1H), 2.47 (d, J = 7.2 Hz, 2H), 1.89 – 1.66 (m, 8H), 1.58 (d, J = 7.2 Hz, 3H), 1.25 – 1.13 (m, 7H), 0.91 (d, J = 6.6 Hz, 6H). **^{13}C{^{1}H} NMR** (126 MHz, CDCl3) δ 173.8, 173.6, 145.3, 142.8, 140.7, 137.5, 129.5, 127.3, 122.0, 114.0, 62.5, 60.9, 45.3, 45.1, 41.3, 30.2, 29.6, 29.2, 26.2, 26.12, 26.08, 22.5, 18.6, 14.3.

Ethyl 2-(phenylamino)heptadecanoate (29)[2c]

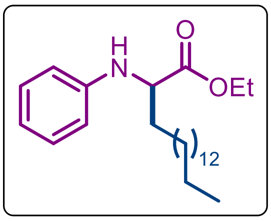

The compound was prepared according to GP using ethyl phenylglycinate (0.045 g, 0.25 mmol), palmitic acid (0.128 g, 0.50 mmol, 2 equiv). After 8 h, purification by column chromatography (0-5% ethyl acetate in hexane) gave **29** as a white solid (0.056 g, 57%).

^{1}H NMR (500 MHz, CDCl3) δ 7.18 (t, J = 7.9 Hz, 2H), 6.78 (t, J = 7.3 Hz, 1H), 6.70 (d, J = 7.9 Hz, 2H), 4.17 (q, J = 7.1 Hz, 2H), 4.04 (t, J = 6.5 Hz, 1H), 1.86 – 1.77 (m, 2H), 1.40 – 1.25 (m, 29H), 0.89 (d, J = 6.7 Hz, 3H). **^{13}C{^{1}H} NMR** (126 MHz, CDCl3) δ 173.8, 146.0, 129.3, 119.0, 114.3, 61.1, 57.4, 32.8, 31.9, 29.68, 29.67, 29.66, 29.64 (X 2C), 29.59, 29.5, 29.4, 29.34, 29.29, 25.5, 22.7, 14.2, 14.1. **HRMS-ESI** (m/z): calcd for $C_{16}H_{28}NO_2$ $[M+H]^+$ 278.2115; found 278.2155.

Ethyl 2-(phenylamino)-3-propylhexanoate (30)

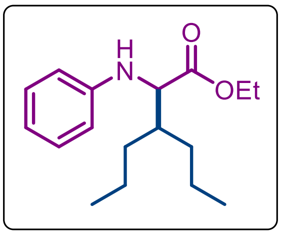

The compound was prepared according to GP using ethyl phenylglycinate (0.045 g, 0.25 mmol), 2-propylpentanoic acid (0.072 g, 0.50 mmol, 2 equiv). After 8 h, purification by column chromatography (0-5% ethyl acetate in hexane) gave **30** as a white solid (0.043 g, 62%).

^{1}H NMR (500 MHz, $CDCl_3$) δ 7.20 (t, *J* = 7.9 Hz, 2H), 6.76 (t, *J* = 7.3 Hz, 1H), 6.67 (d, *J* = 8.0 Hz, 2H), 4.25 – 4.17 (m, 2H), 4.11 (d, *J* = 4.9 Hz, 1H), 1.93 – 1.90 (m, 1H), 1.48 – 1.33 (m, 8H), 1.28 (t, *J* = 7.1 Hz, 3H), 0.97 – 0.93 (m, 6H). **^{13}C{^{1}H} NMR** (126 MHz, $CDCl_3$) δ 173.9, 147.4, 129.3, 118.2, 113.6, 60.8, 59.0, 40.7, 32.7, 32.2, 20.33, 20.29, 14.32, 14.27, 14.26. **HRMS-ESI** (m/z): calcd for $C_{16}H_{28}NO_2$ $[M+H]^+$ 278.2115; found 278.2155.

Ethyl 6-(2,5-dimethylphenoxy)-3,3-dimethyl-2-(phenylamino)hexanoate (31)

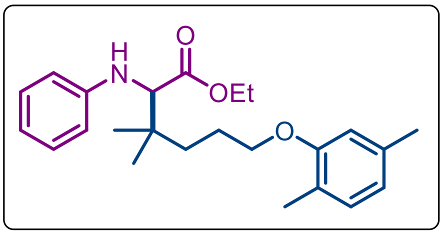

The compound was prepared according to GP using ethyl phenylglycinate (0.045 g, 0.25 mmol), 5-(2,5-dimethylphenoxy)-2,2-dimethylpentanoic acid (0.125 g, 0.50 mmol, 2 equiv). After 8 h, purification by column chromatography (0-10% ethyl acetate in hexane) gave **31** as a white solid (0.069 g, 72%).

^{1}H NMR (500 MHz, $CDCl_3$) δ 7.18 (t, *J* = 7.9 Hz, 2H), 7.01 (d, *J* = 7.4 Hz, 1H), 6.75 (t, *J* = 7.3 Hz, 1H), 6.68 (t, *J* = 8.4 Hz, 3H), 6.61 (s, 1H), 4.16 (q, *J* = 7.1 Hz, 2H), 3.94 – 3.91 (m, 3H), 2.31 (s, 3H), 2.17 (s, 3H), 1.94 – 1.79 (m, 2H), 1.65 – 1.55 (m, 2H), 1.23 (t, *J* = 7.1 Hz, 3H), 1.10 (s, 3H), 1.08 (s, 3H). **^{13}C{^{1}H} NMR** (126 MHz, $CDCl_3$) δ 173.3, 157.0, 147.6, 136.5, 130.3, 129.3, 123.6, 120.7, 118.5, 114.0, 112.0, 68.3, 64.2, 60.7, 36.8, 36.1, 24.1, 24.0, 23.6, 21.4, 15.8, 14.3. **HRMS-ESI** (m/z): calcd for $C_{24}H_{34}NO_3$ $[M+H]^+$ 384.2533; found 384.2545.

Ethyl 6-(2,5-dimethylphenoxy)-2-((4-methoxyphenyl)amino)-3,3-dimethylhexanoate (32)[4d]

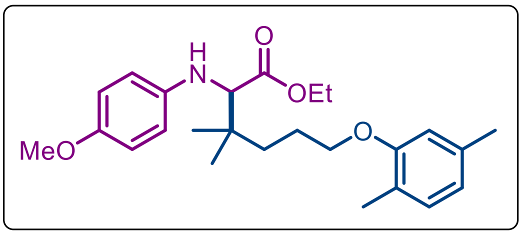

The compound was prepared according to GP using ethyl (4-methoxyphenyl)glycinate (0.052 g, 0.25 mmol), 5-(2,5-dimethylphenoxy)-2,2-dimethylpentanoic acid (0.125 g, 0.50 mmol, 2 equiv). After 8 h, purification by column chromatography (0-15% ethyl acetate in hexane) gave **32** as a white solid (0.074 g, 72%).

^{1}H NMR (500 MHz, $CDCl_3$) δ 7.00 (d, J = 7.4 Hz, 1H), 6.76 (d, J = 8.8 Hz, 2H), 6.68 – 6.63 (m, 3H), 6.61 (s, 1H), 4.17 – 4.10 (m, 2H), 3.93 (t, J = 6.4 Hz, 2H), 3.78 (s, 1H), 3.74 (s, 3H), 2.30 (s, 3H), 2.17 (s, 3H), 1.92 – 1.78 (m, 2H), 1.65 – 1.54 (m, 2H), 1.22 (t, J = 7.1 Hz, 3H), 1.09 (s, 3H), 1.07 (s, 3H). **^{13}C{^{1}H} NMR** (126 MHz, $CDCl_3$) δ 173.6, 157.0, 152.9, 141.7, 136.5, 130.3, 123.6, 120.7, 115.7, 114.8, 112.0, 68.3, 65.7, 60.6, 55.7, 36.7, 36.1, 24.1, 24.0, 23.5, 21.4, 15.8, 14.3.

Ethyl 3-(4-chlorophenoxy)-2-((4-methoxyphenyl)amino)-3-methylbutanoate (33)

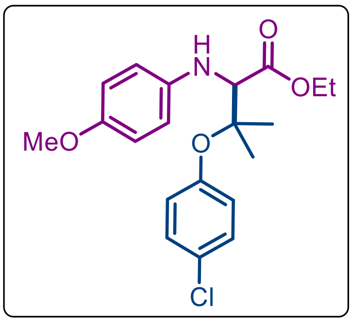

The compound was prepared according to GP using ethyl (4-methoxyphenyl)glycinate (0.052 g, 0.25 mmol), 2-(4-chlorophenoxy)-2-methylpropanoic acid (0.107 g, 0.50 mmol, 2 equiv). After 8 h, purification by column chromatography (0-15% ethyl acetate in hexane) gave **33** as a white solid (0.063 g, 67%).

^{1}H NMR (500 MHz, $CDCl_3$) 7.23 (d, J = 8.7 Hz, 2H), 6.93 (d, J = 8.7 Hz, 2H), 6.78 (d, J = 8.9 Hz, 2H), 6.67 (d, J = 8.9 Hz, 2H), 4.25 – 4.15 (m, 2H), 4.03 (s, 1H), 3.75 (s, 3H), 1.41 (s, 6H), 1.23 (t, J = 7.1 Hz, 3H). **^{13}C{^{1}H} NMR** (126 MHz, $CDCl_3$) δ 172.4, 153.1, 152.9, 141.1, 129.5, 129.2, 125.7, 115.7, 115.0, 81.6, 66.5, 61.1, 55.8, 24.5, 24.3, 14.4. **HRMS-ESI** (m/z): calcd for $C_{20}H_{25}ClNO_4$ $[M+H]^+$ 378.1467; found 378.1476.

4-((6-(2,5-Dimethylphenoxy)-1-ethoxy-3,3-dimethyl-1-oxohexan-2-yl)amino)phenyl stearate (34)

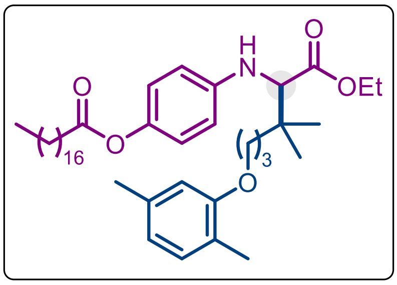

The compound was prepared according to GP using 4-((2-ethoxy-2-oxoethyl)amino)phenyl stearate (0.115 g, 0.25 mmol), 5-(2,5-dimethylphenoxy)-2,2-dimethylpentanoic acid (0.125 g, 0.50 mmol, 2 equiv). After 8 h, purification by column chromatography (0-15% ethyl acetate in hexane) gave **34** as a white solid (0.105 g, 63%).

^{1}H NMR (500 MHz, $CDCl_3$) 7.00 (d, J = 7.5 Hz, 1H), 6.88 (d, J = 8.8 Hz, 2H), 6.65 (dd, J = 7.4, 4.9 Hz, 3H), 6.61 (s, 1H), 4.15 (q, J = 7.1 Hz, 2H), 3.92 (t, J = 6.4 Hz, 2H), 3.84 (s, 1H), 2.50 (t, J = 7.5 Hz, 2H), 2.30 (s, 3H), 2.17 (s, 3H), 1.91 – 1.78 (m, 2H), 1.76 – 1.69 (m, 2H), 1.63 – 1.55 (m, 2H), 1.42 – 1.36 (m, 2H), 1.33 – 1.25 (m, 26H), 1.23 (t, J = 7.1 Hz, 3H), 1.08 (s, 3H), 1.07 (s, 3H), 0.89 (t, J = 6.9 Hz, 3H). **^{13}C{^{1}H} NMR** (126 MHz, $CDCl_3$) δ 173.2, 173.0, 157.1, 145.3, 143.1, 136.6, 130.4, 123.7, 122.3, 120.8, 114.8, 112.1, 68.4, 65.0, 62.5, 60.9, 37.0, 36.2, 34.5, 32.1, 29.8 (X 3C), 29.82, 29.79, 29.7, 29.6, 29.5, 29.4, 29.3, 25.2, 24.2, 24.1,

23.7, 22.8, 21.5, 15.9, 15.2, 14.4, 14.3. **HRMS-ESI** (m/z): calcd for $C_{42}H_{67}NO_5$ $[M]^+$ 665.5019; found 665.5033.

Methyl (2-cyclohexyl-2-(phenylamino)acetyl)glycinate (35)[4a]

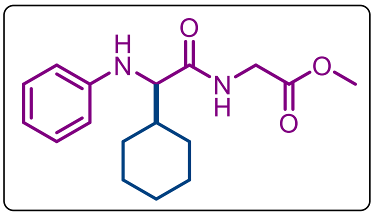

The compound was prepared according to GP using methyl phenylglycylglycinate (0.056 g, 0.25 mmol), cyclohexanecarboxylic acid (0.064 g, 0.50 mmol, 2 equiv). After 8 h, purification by column chromatography (10-25% ethyl acetate in hexane) gave **35** as a white solid (0.047 g, 62%).

^{1}H NMR (500 MHz, $CDCl_3$) 7.25 – 7.16 (m, 3H), 6.80 (t, *J* = 7.3 Hz, 1H), 6.64 (d, *J* = 8.1 Hz, 2H), 4.16 (dd, *J* = 18.2, 6.4 Hz, 1H), 3.90 (dd, *J* = 18.2, 5.0 Hz, 1H), 3.70 (s, 3H), 3.62 (d, *J* = 4.3 Hz, 1H), 2.06 – 2.00 (m, 1H), 1.82 – 1.67 (m, 5H), 1.34 – 1.15 (m, 5H). **^{13}C{^{1}H} NMR** (126 MHz, $CDCl_3$) δ 173.2, 170.1, 147.1, 129.4, 119.2, 113.9, 64.8, 52.2, 41.1, 40.9, 30.2, 28.1, 26.23, 26.20, 26.1.

Methyl (2-cyclohexyl-2-(phenylamino)acetyl)-*L*-valinate (36)[1c]

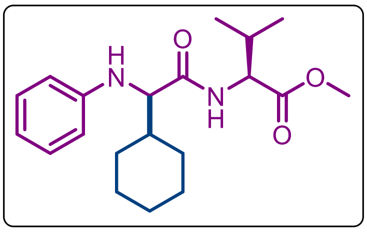

The compound was prepared according to GP using phenylglycyl-*L*-valinate (0.064 g, 0.25 mmol), cyclohexanecarboxylic acid (0.064 g, 0.50 mmol, 2 equiv). After 8 h, purification by column chromatography (10-30% ethyl acetate in hexane) gave **36** as a white solid (0.045 g, 54%, dr = 1.3:1).

^{1}H NMR (500 MHz, $CDCl_3$) δ 7.20 – 7.13 (m, 3H), 6.81 – 6.77 (m, 1H), 6.67 – 6.63 (m, 2H), 4.56 – 4.49 (m, 1H), 3.72 – 3.58 (m, 4H), 2.17 – 2.00 (m, 2H), 1.81 – 1.67 (m, 6H), 1.34 – 1.14 (m, 5H), 0.91 – 0.85 (m, 3H), 0.81 – 0.67 (m, 3H). **^{13}C{^{1}H} NMR** (126 MHz, $CDCl_3$) δ 172.8, 172.6, 172.3, 171.8, 147.0, 146.9, 129.32, 129.25, 119.5, 119.1, 114.4, 113.8, 65.5, 64.7, 57.2, 56.7, 52.1, 51.9, 41.1, 41.0, 31.0, 30.9, 30.3, 30.2, 29.7, 29.6, 28.4, 27.9, 26.3, 26.23, 26.18, 26.1, 19.1, 19.0, 17.9, 17.4.

Methyl (2-cyclohexyl-2-(phenylamino)acetyl)-*L*-methioninate (37)[1c]

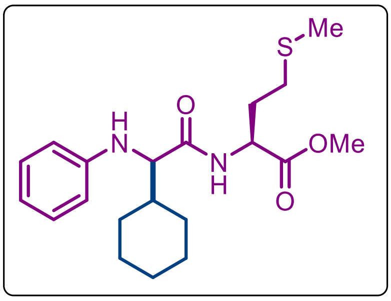

The compound was prepared according to GP using methyl phenylglycyl-*L*-methioninate (0.074 g, 0.25 mmol), cyclohexanecarboxylic acid (0.064 g, 0.50 mmol, 2 equiv). After 8 h, purification by column chromatography (10-30% ethyl acetate in hexane) gave **37** as a white solid (0.049 g, 52%, dr = 1.1:1).

^{1}H NMR (500 MHz, $CDCl_3$) δ 7.37 – 7.29 (m, 1H), 7.23 – 7.20 (m, 2H), 6.85 – 6.80 (m, 1H), 6.70 (d, J = 7.9 Hz, 1H), 6.64 (d, J = 8.0 Hz, 1H), 4.79 – 4.69 (m, 1H), 3.75 – 3.65 (m, 3H), 3.66 – 3.63 (m, 1H), 2.47 (t, J = 7.4 Hz, 1H), 2.32 – 2.25 (m, 1H), 2.22 – 2.10 (m, 1H), 2.07 – 1.87 (m, 5H), 1.84 – 1.69 (m, 5H), 1.31 – 1.20 (m, 5H). **^{13}C{^{1}H} NMR** (126 MHz, $CDCl_3$) δ 172.7, 172.6, 172.2, 171.8, 146.80, 146.76, 129.4, 129.3, 119.6, 119.2, 114.4, 113.7, 65.3, 64.6, 52.44, 52.35, 51.4, 50.9, 41.1, 41.0, 31.4, 31.3, 30.2, 30.10, 30.05, 29.73, 29.68, 28.4, 28.1, 26.3, 26.21, 26.15, 26.08, 26.07, 15.4, 15.2.

Methyl (3*R*)-2-(2-cyclohexyl-2-(phenylamino)acetamido)-3-hydroxybutanoate (38)[1c]

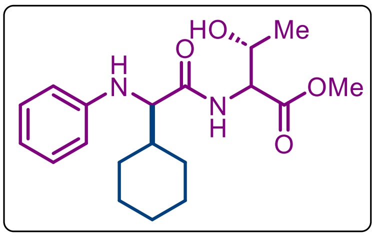

The compound was prepared according to GP using methyl phenylglycyl-*L*-threoninate (0.067 g, 0.25 mmol), cyclohexanecarboxylic acid (0.064 g, 0.50 mmol, 2 equiv). After 8 h, purification by column chromatography (10-25% acetone in hexane) gave **38** as a white solid (0.037 g, 43%, dr = 1.1:1).

^{1}H NMR (500 MHz, $CDCl_3$) δ 7.38 (dd, J = 14.0, 9.4 Hz, 1H), 7.18 (t, J = 7.8 Hz, 2H), 6.81 – 6.76 (m, 1H), 6.68 – 6.63 (m, 2H), 4.58 – 4.54 (m, 1H), 4.38 – 4.28 (m, 1H), 3.73 – 3.62 (m, 4H), 2.06 – 1.98 (m, 1H), 1.84 – 1.67 (m, 5H), 1.33 – 1.18 (m, 5H), 1.17 – 1.01 (m, 3H). **^{13}C{^{1}H} NMR** (126 MHz, $CDCl_3$) δ 173.6, 173.5, 171.3, 171.1, 147.02, 146.96, 129.4, 129.3, 119.4, 119.1, 114.4, 113.8, 67.9, 67.6, 65.3, 64.8, 57.3, 56.9, 52.5, 52.4, 41.2, 41.0, 30.23, 30.17, 28.5, 28.2, 26.29, 26.25, 26.2 (X 2C), 26.14, 26.12, 19.9, 19.8.

Methyl (2-cyclohexyl-2-(phenylamino)acetyl)-*L*-valyl-*L*-phenylalaninate (39)[1c]

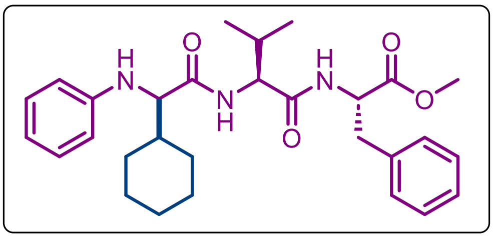

The compound was prepared according to GP using methyl phenylglycyl-*L*-valyl-*L*-phenylalaninate (0.103 g, 0.25 mmol), cyclohexanecarboxylic acid (0.064 g, 0.50 mmol, 2 equiv). After 8 h, purification by column chromatography (10-30% acetone in hexane) gave **39** as a white solid (0.069 g, 56%, dr = 1.2:1).

^{1}H NMR (500 MHz, $CDCl_3$) δ 7.21 – 7.17 (m, 1H), 7.13 – 7.05 (m, 5H), 6.98 (d, *J* = 6.8 Hz, 1H), 6.86 (d, *J* = 6.9 Hz, 1H), 6.68 – 6.64 (m, 1H), 6.52 (t, *J* = 8.6 Hz, 2H), 6.47 – 6.35 (m, 1H), 4.73 – 4.59 (m, 1H), 4.19 – 4.11 (m, 1H), 3.94 (br s, 1H), 3.72 – 3.66 (m, 1H), 3.59 – 3.52 (m, 3H), 3.00 – 2.62 (m, 2H), 2.03 – 1.86 (m, 2H), 1.66 (dd, *J* = 10.4, 7.8 Hz, 2H), 1.61 – 1.56 (m, 2H), 1.20 – 1.04 (m, 5H), 0.75 – 0.70 (m, 3H), 0.58 – 0.55 (m, 3H), 0.43 – 0.42 (m, 1H). **^{13}C{^{1}H} NMR** (126 MHz, $CDCl_3$) δ 173.22, 173.15, 171.7, 171.6, 170.9, 170.6, 147.5, 147.2, 136.0, 135.7, 129.7, 129.4, 129.2, 129.1, 128.7, 128.6, 127.3, 127.1, 120.0, 119.2, 118.9, 113.69, 113.67, 64.9, 64.4, 62.5, 58.4, 58.3, 53.31, 53.27, 52.4, 41.3, 41.1, 37.9, 37.8, 34.1, 30.8, 30.33, 30.25, 30.0, 28.7, 28.3, 26.3, 26.24, 26.19, 19.3, 19.2, 18.7, 17.9, 17.7, 15.5.

Methyl (2-cyclohexyl-2-(phenylamino)acetyl)-*L*-tryptophyl-*L*-alaninate (40)[1c]

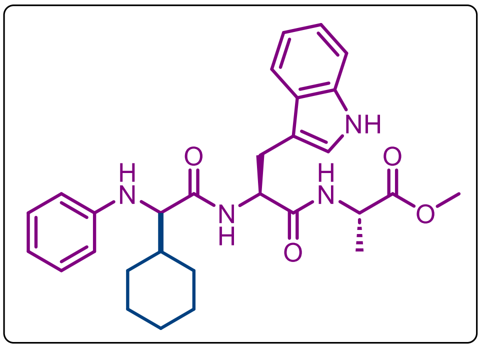

The compound was prepared according to GP using methyl phenylglycyl-*L*-tryptophyl-*L*-alaninate (0.106 g, 0.25 mmol), cyclohexanecarboxylic acid (0.064 g, 0.50 mmol, 2 equiv). After 8 h, purification by column chromatography (10-25% acetone in hexane) gave **40** as a white solid (0.057 g, 45%, dr = 1.5:1).

^{1}H NMR (500 MHz, $CDCl_3$) δ 8.26 – 7.90 (m, 1H), 7.64 – 7.57 (m, 1H), 7.37 – 7.26 (m, 2H), 7.21 – 7.11 (m, 4H), 7.06 – 7.01 (m, 1H), 6.80 – 6.72 (m, 1H), 6.60 – 6.53 (m, 1H), 6.49 (d, *J* = 8.0 Hz, 2H), 4.76 – 4.70 (m, 1H), 4.48 – 4.40 (m, 1H), 3.70 – 3.67 (m, 3H), 3.57 – 3.54 (m, 1H), 3.28 – 3.01 (m, 2H), 1.86 – 1.55 (m, 6H), 1.47 – 1.37 (m, 1H), 1.25 – 1.17 (m, 5H), 1.16 – 1.06 (m, 2H). **^{13}C{^{1}H} NMR** (126 MHz, $CDCl_3$) δ 173.3 (X 2C), 172.9, 172.8, 170.8, 170.6, 147.2, 147.0, 136.24, 136.18, 129.5, 129.4, 127.7, 127.1, 123.5, 123.1, 122.2, 122.1, 119.6 (X 2C), 118.94, 118.85, 118.8, 118.6, 113.5, 113.3, 111.3, 111.1, 110.6, 109.9, 64.6, 64.2, 53.8, 52.9, 52.4 (X 2C), 48.2, 47.9, 41.2, 40.8, 30.1, 29.9, 29.7, 29.6, 28.4, 27.8, 27.5, 27.0, 26.13, 26.12, 26.03, 25.95, 18.14, 18.08.

Methyl (2-cyclohexyl-2-(phenylamino)acetyl)glycyl-*L*-phenylalanyl-*L*-leucinate (41)[1c]

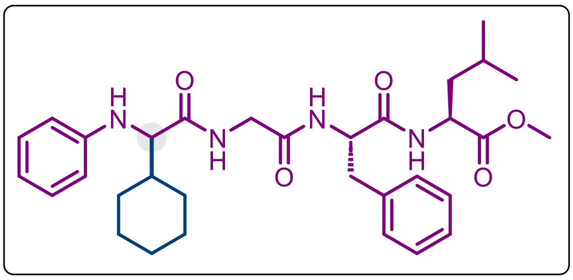

The compound was prepared according to GP using methyl methyl phenylglycylglycyl-*L*-phenylalanyl-*L*-leucinate (0.120 g, 0.25 mmol), cyclohexanecarboxylic acid (0.064 g, 0.50 mmol, 2 equiv). After 8 h, purification by column chromatography (10-25% acetone in hexane) gave **41** as a white solid (0.073 g, 52%, dr = 2.5:1).

^{1}H NMR (500 MHz, $CDCl_3$) δ 7.47 – 7.39 (m, 1H), 7.27 – 7.15 (m, 6H), 7.09 (d, *J* = 7.0 Hz, 1H), 6.95 – 6.89 (m, 1H), 6.78 (dd, *J* = 16.7, 7.6 Hz, 1H), 6.69 – 6.56 (m, 3H), 4.70 (q, *J* = 7.1 Hz, 1H), 4.55 – 4.49 (m, 1H), 4.02 – 3.81 (m, 2H), 3.70 – 3.68 (m, 4H), 3.08 – 2.87 (m, 2H), 2.00 – 1.86 (m, 1H), 1.79 – 1.68 (m, 5H), 1.62 – 1.49 (m, 3H), 1.31 – 1.16 (m, 5H), 0.91 – 0.86 (m, 6H). **^{13}C{^{1}H} NMR** (126 MHz, $CDCl_3$) δ 175.9, 175.8, 174.81, 174.77, 172.4, 172.3, 170.7, 170.6, 149.4 (X 2C), 138.4, 138.3, 131.6, 131.5, 131.4, 131.3, 130.6, 130.5, 129.0, 128.9, 120.9, 120.8, 115.5, 115.4, 66.33, 66.27, 56.42, 56.37, 54.3, 52.98, 52.96, 44.91, 44.85, 43.24, 43.19, 43.1, 40.3, 40.2, 35.4, 32.9, 32.18, 32.16, 30.5, 30.4, 28.4, 28.2, 28.1, 26.8, 26.7, 24.7, 24.6, 23.9.

Methyl (2-cyclohexyl-2-(phenylamino)acetyl)glycyl-*L*-phenylalanyl-*L*-methioninate (42)[1c]

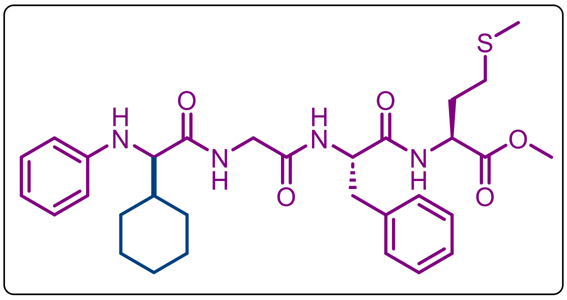

The compound was prepared according to GP using methyl phenylglycylglycyl-*L*-phenylalanyl-*L*-methioninate (0.125 g, 0.25 mmol), cyclohexanecarboxylic acid (0.064 g, 0.50 mmol, 2 equiv). After 8 h, purification by column chromatography (10-30% acetone in hexane) gave **42** as a white solid (0.064 g, 44%, dr = 1.1:1).

^{1}H NMR (500 MHz, $CDCl_3$) δ 7.51 (dd, *J* = 7.7, 1.1 Hz, 1H), 7.39 (d, *J* = 1.4 Hz, 1H), 7.20 – 7.08 (m, 8H), 6.72 (dd, *J* = 7.0, 5.1 Hz, 1H), 6.60 (dd, *J* = 9.0, 6.2 Hz, 2H), 4.73 (s, 1H), 4.62 – 4.55 (m, 1H), 4.30 (br s, 1H), 4.02 – 3.77 (m, 2H), 3.69 – 3.67 (m, 4H), 3.07 – 2.83 (m, 2H), 2.43 – 2.41 (m, 2H), 2.08 – 1.90 (m, 6H), 1.76 – 1.74 (m, 3H), 1.67 – 1.65 (m, 1H), 1.29 – 1.11 (m, 6H). **^{13}C{^{1}H} NMR** (126 MHz, $CDCl_3$) δ 174.0, 173.9, 171.9, 171.8, 170.7, 170.6, 168.9, 168.8, 147.5, 147.4, 136.4, 136.3, 129.5, 129.4, 129.34, 129.27, 128.52, 128.49, 126.93,

126.88, 118.7, 118.6, 113.5, 113.4, 64.13, 64.05, 54.5, 52.4 (X 2C), 51.6 (X 2C), 42.87, 42.85, 41.18, 41.15, 38.2, 38.1, 31.21, 31.20, 30.13, 30.09, 30.0, 29.9, 29.70, 29.68, 29.64, 28.60, 28.5, 28.3, 26.2 (X 2C), 26.1, 15.4 (X 2C).

Methyl (2-cyclohexyl-2-(phenylamino)acetyl)glycyl-*L*-leucyl-*L*-tryptophyl-*L*-alaninate (43)[1c]

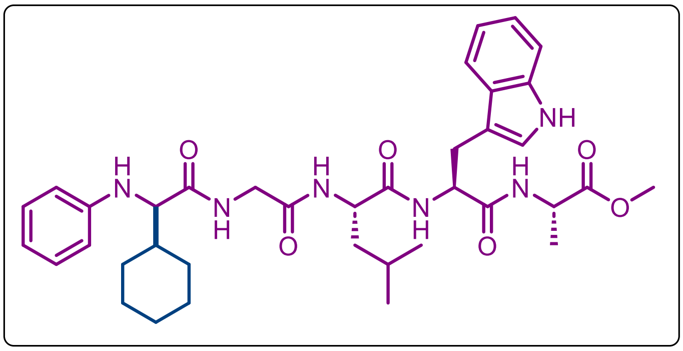

The compound was prepared according to GP using methyl phenylglycyl-glycyl-*L*-leucyl-*L*-tryptophyl-*L*-alaninate (0.148 g, 0.25 mmol), cyclohexanecarboxylic acid (0.064 g, 0.50 mmol, 2 equiv). After 8 h, purification by column chromatography (10-30% acetone in hexane) gave **43** as a white solid (0.076 g, 45%, dr > 20:1).

^{1}H NMR (500 MHz, DMSO) δ 10.79 (s, 1H), 8.31 – 8.24 (m, 1H), 8.18 – 8.10 (m, 1H), 8.04 – 7.98 (m, 1H), 7.85 – 7.71 (m, 1H), 7.62 – 7.56 (m, 1H), 7.36 – 7.29 (m, 1H), 7.14 – 7.10 (m, 1H), 7.07 – 7.02 (m, 3H), 7.01 – 6.95 (m, 1H), 6.64 – 6.59 (m, 2H), 6.57 – 6.50 (m, 1H), 5.61 – 5.56 (m, 1H), 4.56 – 4.53 (m, 1H), 4.31 – 4.26 (m, 2H), 3.72 – 3.68 (m, 2H), 3.61 – 3.59 (m, 4H), 3.18 – 3.12 (m, 1H), 2.99 – 2.92 (m, 1H), 1.85 – 1.62 (m, 6H), 1.54 – 1.26 (m, 9H), 1.10 – 0.99 (m, 2H), 0.91 – 0.71 (m, 6H). **^{13}C{^{1}H} NMR** (126 MHz, DMSO) δ 173.44, 173.42, 173.33, 173.32, 172.02, 172.01, 171.68, 171.67, 168.94 (X 2C), 148.92, 148.90, 136.4, 129.23, 129.20, 129.16, 127.78, 124.0, 121.2, 118.9, 118.6, 116.5, 113.0, 113.0, 111.70, 111.65, 110.4, 110.3, 69.0 (X 2C), 62.70, 62.68, 56.3, 53.40, 53.38, 52.3, 51.34, 51.28, 48.1, 42.4, 42.3, 41.5, 41.4, 41.0 (X 2C), 32.6, 30.04 (X 2C), 29.98, 29.7, 29.6, 29.3, 29.2, 27.9, 26.33, 26.25, 24.44, 24.41, 23.46, 23.45, 22.11, 22.06, 17.30 (X 2C).

Methyl (2-(phenylamino)-3-propylhexanoyl)-*L*-phenylalaninate (44)

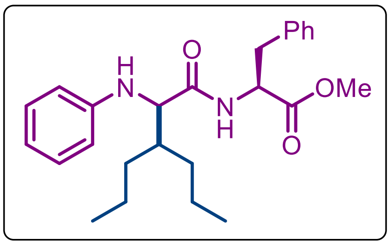

The compound was prepared according to GP using methyl phenylglycyl-*L*-phenylalaninate (0.078 g, 0.25 mmol), 2-propylpentanoic acid (0.072 g, 0.50 mmol, 2 equiv). After 8 h, purification by column chromatography (10-20% acetone in hexane) gave **44** as a white solid (0.047 g, 46%, dr > 20:1).

^{1}H NMR (500 MHz, $CDCl_3$) δ 7.25 – 7.17 (m, 3H), 7.13 (t, J = 7.4 Hz, 1H), 7.05 (t, J = 7.5 Hz, 2H), 6.86 – 6.78 (m, 3H), 6.55 (d, J = 8.1 Hz, 2H), 5.00 – 4.96 (m, 1H), 3.76 (d, J = 3.4

Hz, 1H), 3.69 (s, 3H), 3.07 – 2.95 (m, 2H), 1.42 – 1.35 (m, 3H), 1.29 – 1.23 (m, 5H), 1.18 – 1.13 (m, 1H), 0.88 – 0.82 (m, 6H). **^{13}C{^{1}H} NMR** (126 MHz, $CDCl_3$) δ 172.8, 171.7, 146.9, 135.4, 129.4, 129.2, 128.5, 126.9, 119.1, 113.6, 61.4, 52.3, 52.2, 40.4, 38.0, 33.2, 32.0, 20.8, 20.4, 14.2, 14.1. **HRMS-ESI** (m/z): calcd for $C_{25}H_{35}N_2O_3$ $[M+H]^+$ 411.2642; found 411.2660.

Methyl (6-(2,5-dimethylphenoxy)-3,3-dimethyl-2-(phenylamino)hexanoyl)-*L*-threoninate (45)

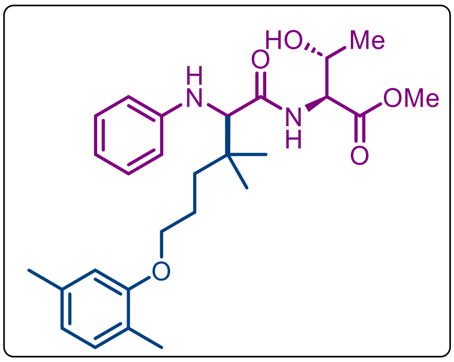

The compound was prepared according to GP using methyl phenylglycyl-*L*-threoninate (0.067 g, 0.25 mmol), 5-(2,5-dimethylphenoxy)-2,2-dimethylpentanoic acid (0.125 g, 0.50 mmol, 2 equiv). After 8 h, purification by column chromatography (10-25% acetone in hexane) gave **45** as a white solid (0.042 g, 36%, dr = 1.8:1).

^{1}H NMR (500 MHz, $CDCl_3$) δ 7.28 (d, J = 9.1 Hz, 1H), 7.21 – 7.16 (m, 2H), 6.99 (d, J = 7.5 Hz, 1H), 6.80 – 6.77 (m, 1H), 6.66 (dd, J = 7.8, 5.2 Hz, 3H), 6.58 (s, 1H), 4.57 – 4.54 (m, 1H), 4.36 – 4.27 (m, 1H), 3.94 – 3.86 (m, 2H), 3.74 – 3.58 (m, 4H), 2.28 (s, 3H), 2.16 (s, 3H), 1.94 – 1.88 (m, 1H), 1.76 – 1.64 (m, 4H), 1.18 – 1.14 (m, 6H), 1.13 – 0.94 (m, 3H). **^{13}C{^{1}H} NMR** (126 MHz, $CDCl_3$) δ 173.0, 172.5, 171.3, 171.1, 156.9 (X 2C), 147.1, 146.8, 136.5 (X 2C), 130.3 (X 2C), 129.4, 129.2, 123.5 (X 2C), 120.70, 120.68, 119.2, 119.1, 114.3, 113.7, 112.0 (X 2C), 68.1, 67.9, 67.7, 67.0, 66.3, 57.3, 56.8, 52.5, 52.3, 36.9, 36.7, 36.6, 36.5, 29.7, 29.6, 24.7, 24.6, 24.31, 24.30, 24.1, 24.0, 21.4, 19.9, 19.7, 15.8 (X 2C). **HRMS-ESI** (m/z): calcd for $C_{27}H_{39}N_2O_5$ $[M+H]^+$ 471.2853; found 471.2857.

Methyl (2-(phenylamino)-3-propylhexanoyl)glycyl-*L*-methioninate (46)

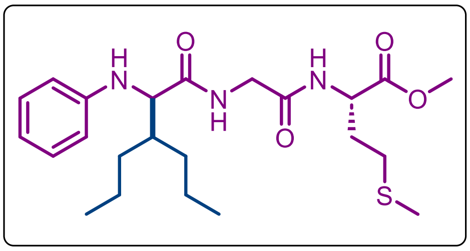

The compound was prepared according to GP using methyl phenylglycylglycyl-*L*-methioninate (0.088 g, 0.25 mmol), 2-propylpentanoic acid (0.072 g, 0.50 mmol, 2 equiv). After 8 h, purification by column chromatography (10-25% acetone in hexane) gave **46** as a white solid (0.058 g, 51%, dr = 1:1).

^{1}H NMR (500 MHz, $CDCl_3$) δ 7.55 – 7.35 (m, 1H), 7.21 – 7.17 (m, 2H), 6.89 (t, J = 8.2 Hz, 1H), 6.83 – 6.76 (m, 1H), 6.64 (d, J = 6.9 Hz, 2H), 4.67 – 4.63 (m, 1H), 4.02 – 3.89 (m, 2H), 3.85 (s, 1H), 3.73 – 3.71 (m, 3H), 2.50 – 2.41 (m, 2H), 2.15 – 2.03 (m, 5H), 1.96 – 1.87 (m, 1H), 1.32 – 1.26 (m, 5H), 0.91 – 0.83 (m, 9H). **^{13}C{^{1}H} NMR** (126 MHz, $CDCl_3$) δ 174.3,

174.2, 172.0 (X 2C), 168.79, 168.76, 146.9, 139.5, 129.6, 129.5, 127.6, 125.0, 119.3, 113.7, 61.54, 61.48, 53.4, 52.5, 51.5, 51.4, 43.2, 43.1, 40.3, 36.6, 33.0, 33.0, 32.2, 31.5, 30.0, 29.9, 29.7, 29.2, 28.4, 24.7, 23.9, 20.6, 20.4, 15.43, 15.40, 14.3, 14.21, 14.15. **HRMS-ESI** (m/z): calcd for $C_{23}H_{38}N_3O_4S$ $[M+H]^+$ 452.2578; found 452.2570.

12. References:

1. (b) C. Wang, R. Qi, H. Xue, Y. Shen, M. Chang, Y. Chen, R. Wang and Z. Xu, *Angew. Chem., Int. Ed.*, 2020, **59**, 7461-7466; (c) P. Meher, M. S. Prasad, K. R. Thombare and S. Murarka, *ACS Catal.*, 2024, **14**, 18896-18906; (d) S. Yang, H. Hu, J. H. Li and M. Chen, *ACS Catal.*, 2023, **13**, 15652-15662.
2. (a) J. Wang, Y. Su, Z. Quan, J. Li, J. Yang, Y. Yuan and C. Huo, *Chem. Commun.*, 2021, **57**, 1959-1962; (c) C. Wang, M. Guo, R. Qi, Q. Shang, Q. Liu, S. Wang, L. Zhao, R. Wang and Z. Xu, *Angew. Chem., Int. Ed.*, 2018, **57**, 15841-15846.
3. S. Song, X. Cheng, S. Cheng, Y.-M. Lin and L. Gong, *Chem. Eur. J.*, 2023, **29**, e202203404.
4. (a) G.-Z. Wang, M.-C. Fu, B. Zhao and R. Shang, *Sci. China Chem.*, 2021, **64**, 439-444; (b) H. Tian, W. Xu, Y. Liu and Q. Wang, *Org. Lett.*, 2020, **22**, 5005-5008; (c) L. Cardinale, M.-O. W. S. Schmotz, M. O. Konev and A. Jacobi von Wangelin, *Org. Lett.*, 2022, **24**, 506-510; (d) X. Sui, H. T. Dang, A. Porey, R. Trevino, A. Das, S. O. Fremin, W. B. Hughes, W. T. Thompson, S. K. Dhakal, H. D. Arman and O. V. Larionov, *Chem. Sci.*, 2024, **15**, 9582-9590.

13. NMR Spectra of Compounds: ^{1}H NMR spectrum of 3 ($CDCl_3$, 500 MHz)

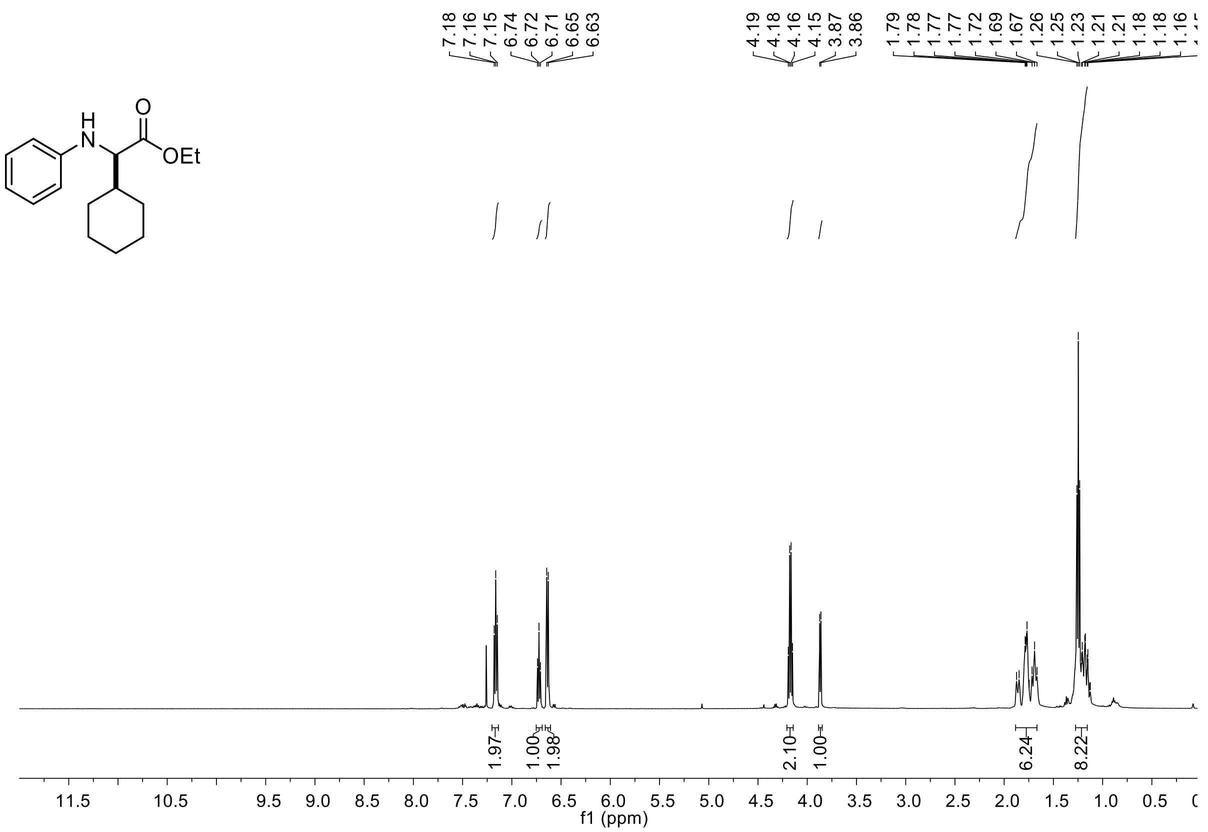

$^{13}C\{^{1}H\}$ NMR spectrum of 3 ($CDCl_3$, 126 MHz)

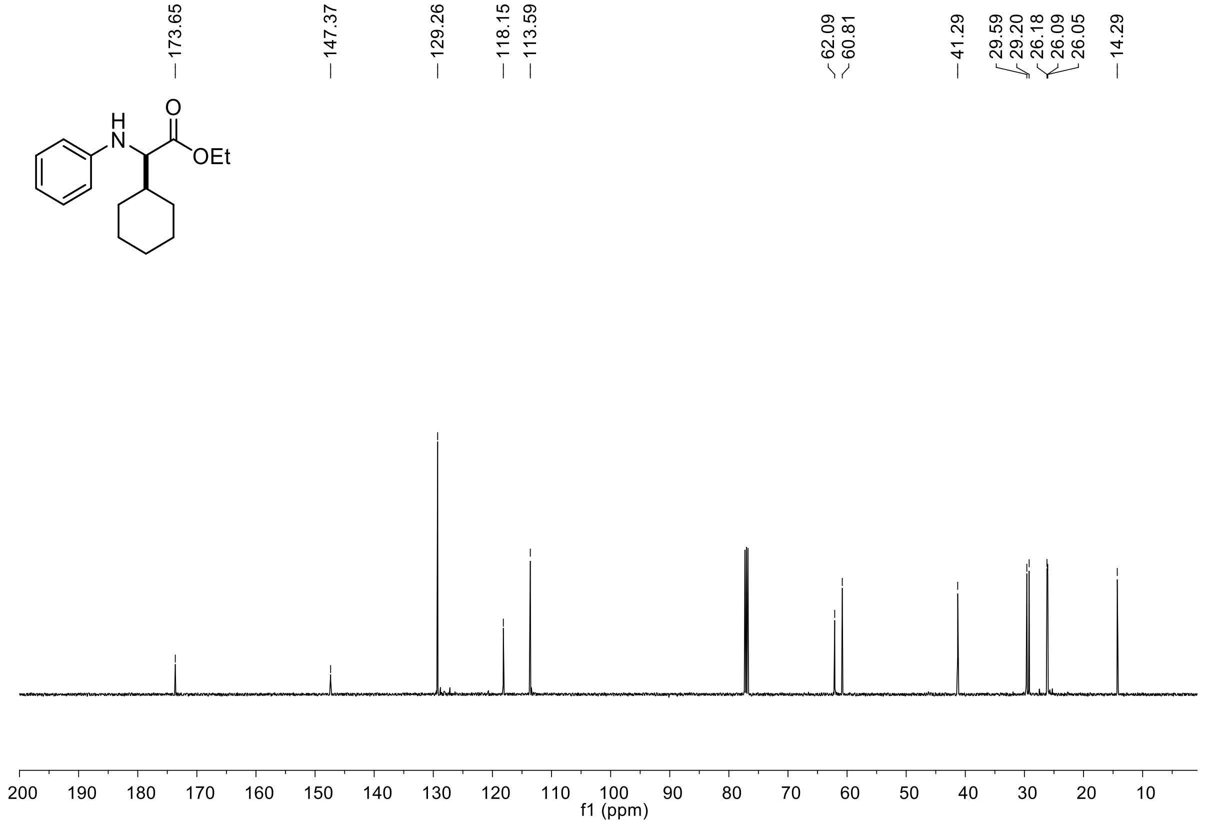

^{1}H NMR spectrum of 4 ($CDCl_3$, 400 MHz)

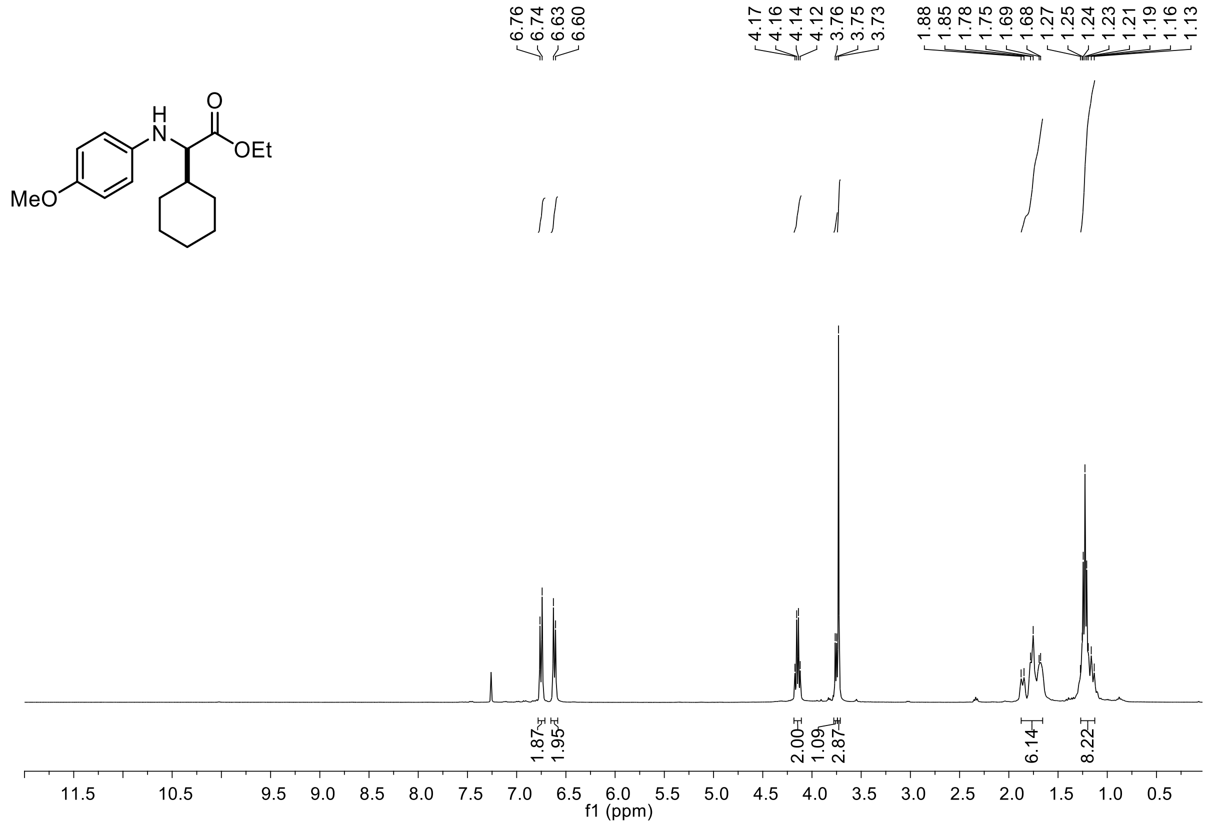

^{13}C{^{1}H} NMR spectrum of 4 ($CDCl_3$, 101 MHz)

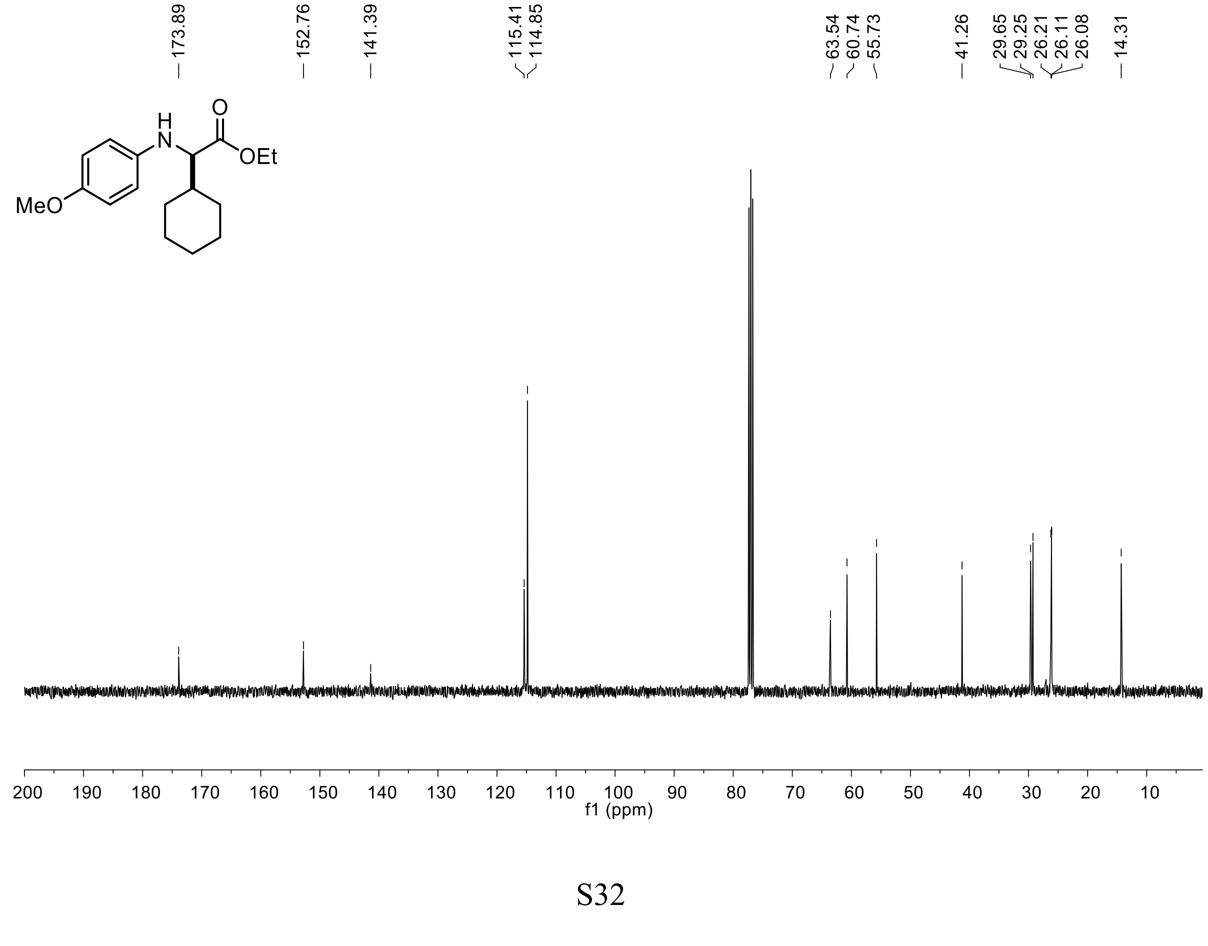

^{1}H NMR spectrum of 5 ($CDCl_3$, 500 MHz)

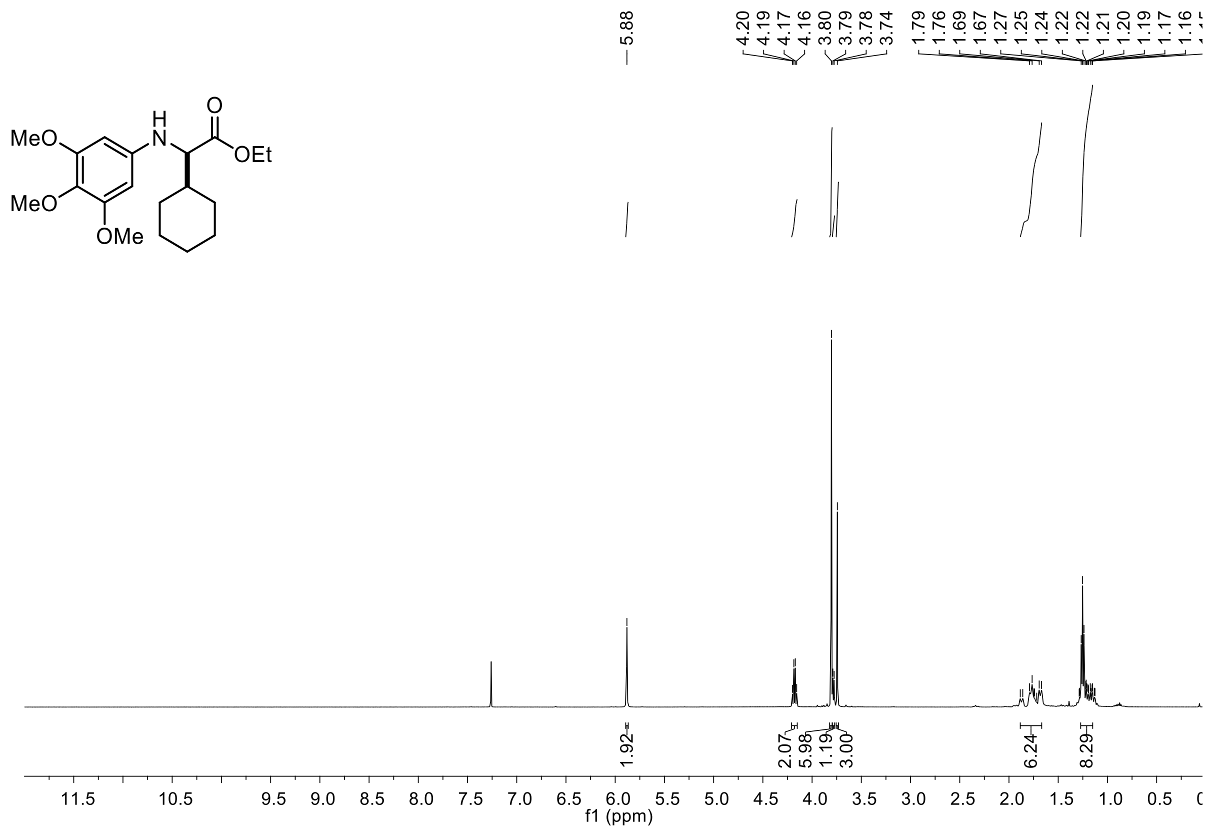

$^{13}C\{^{1}H\}$ NMR spectrum of 5 ($CDCl_3$, 126 MHz)

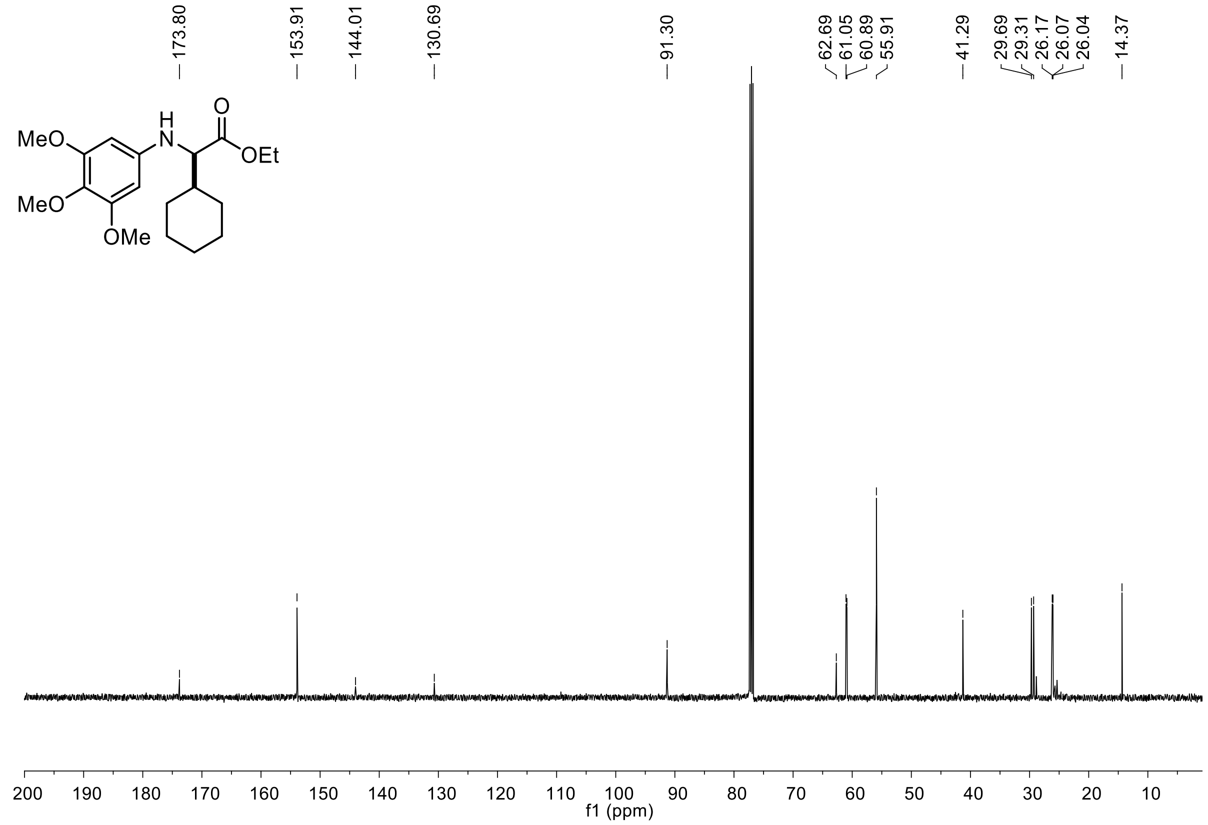

^{1}H NMR spectrum of 6 (CDCl$_3$, 400 MHz)

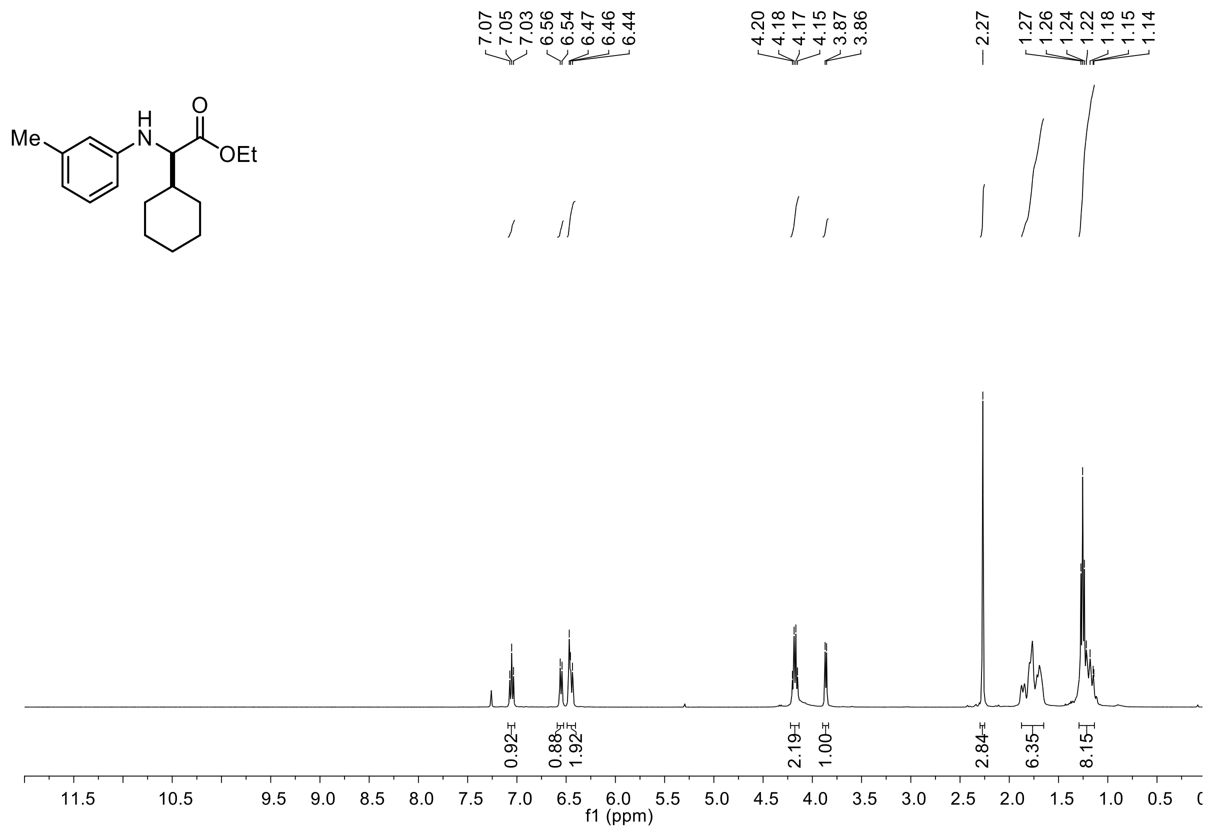

^{13}C{^{1}H} NMR spectrum of 6 (CDCl$_3$, 101 MHz)

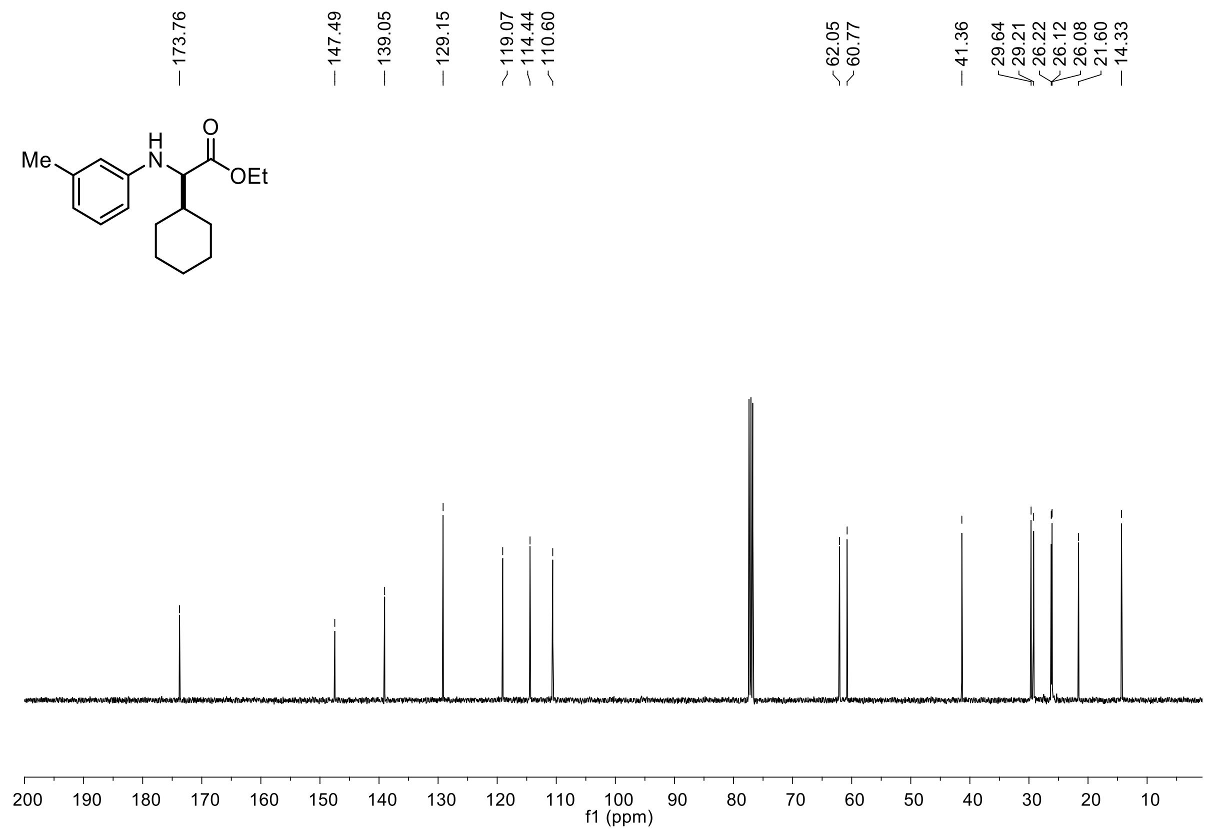

¹H NMR spectrum of 7 ($CDCl_3$, 400 MHz)

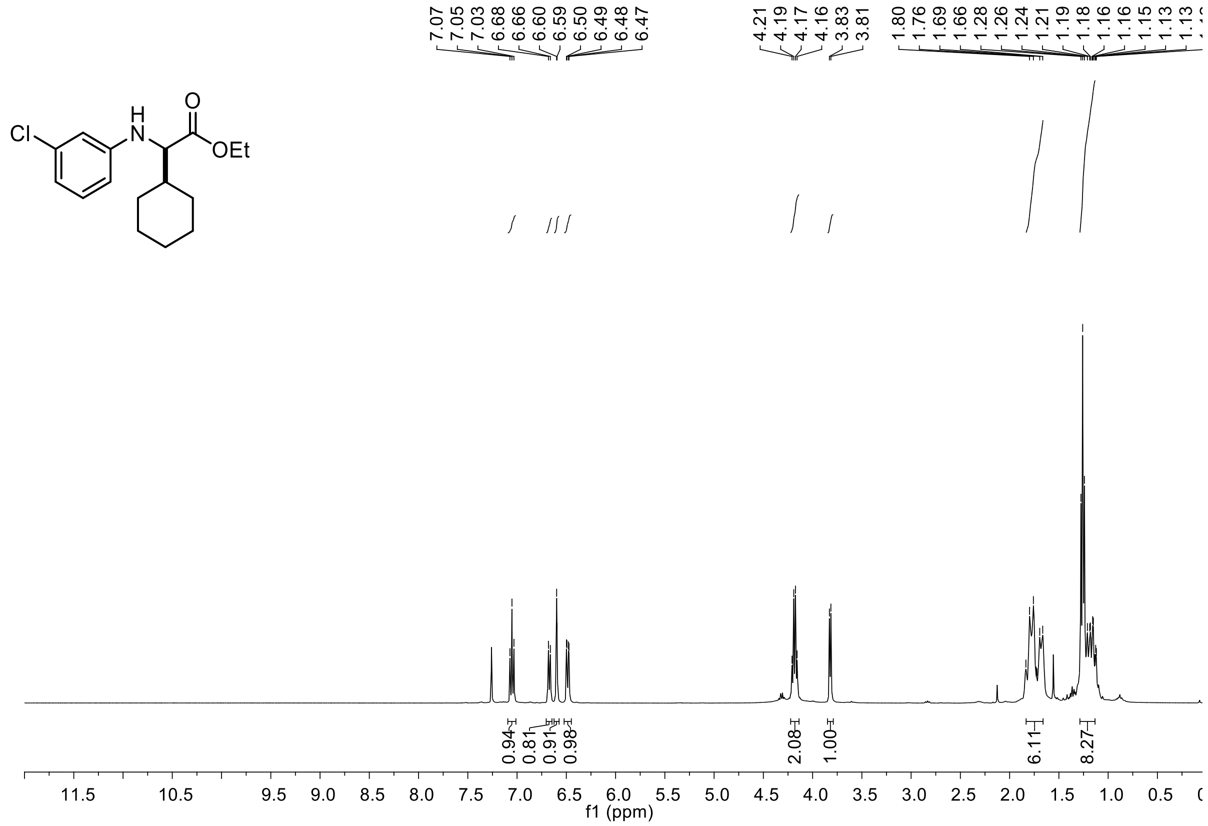

¹³C{¹H} NMR spectrum of 7 ($CDCl_3$, 101 MHz)

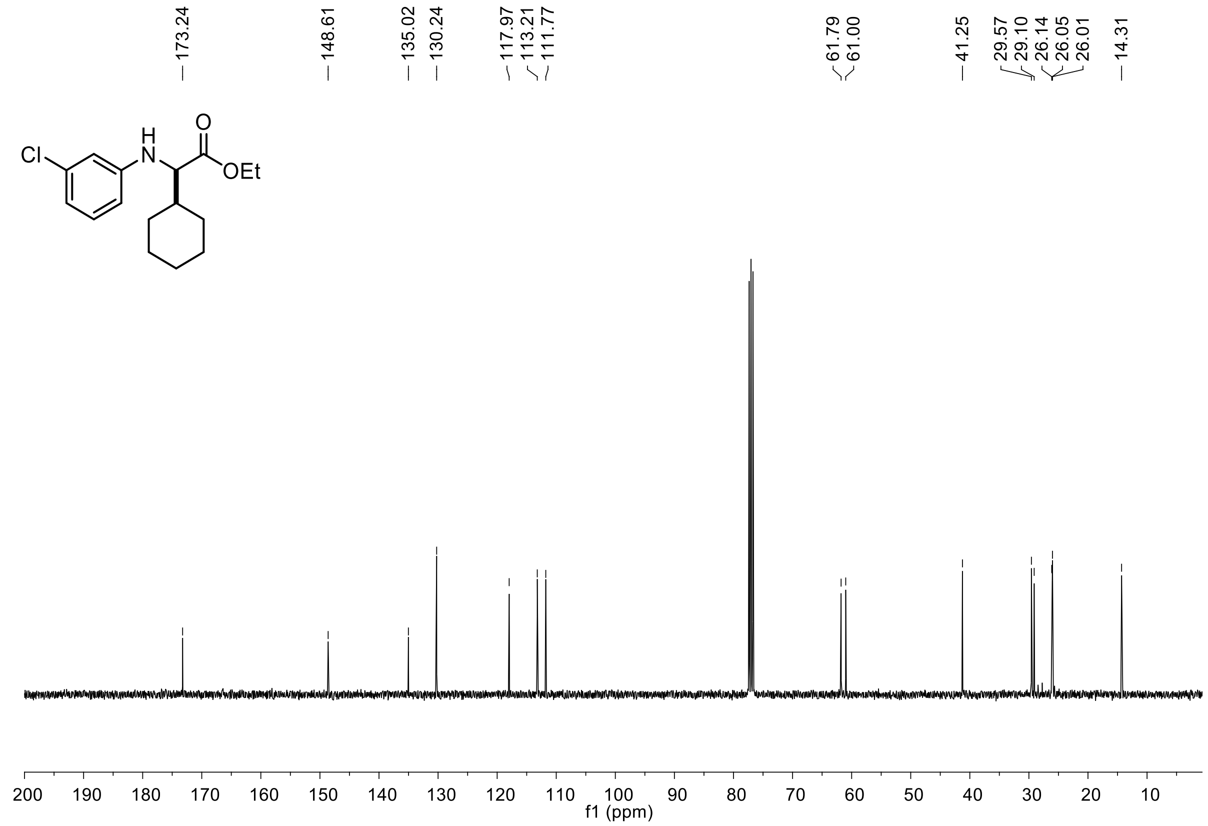

^{1}H NMR spectrum of 8 ($CDCl_3$, 400 MHz)

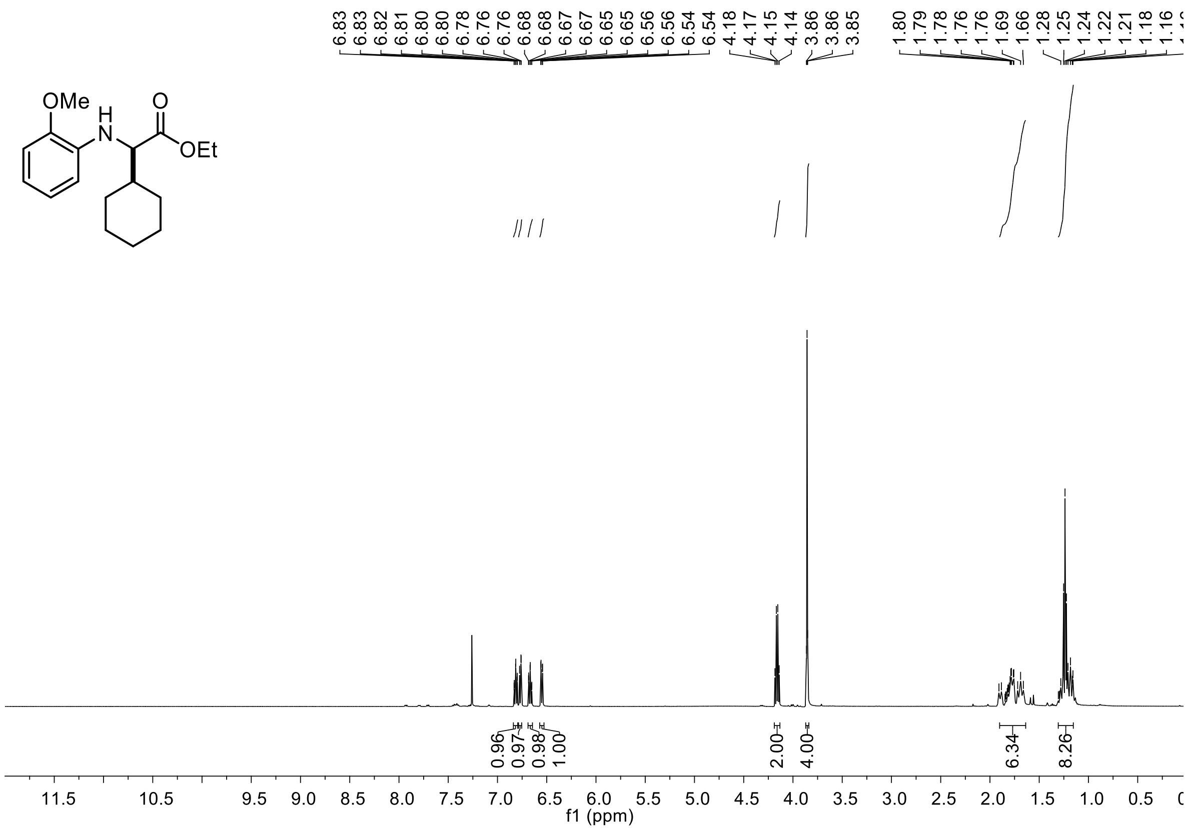

$^{13}C\{^1H\}$ NMR spectrum of 8 ($CDCl_3$, 101 MHz)

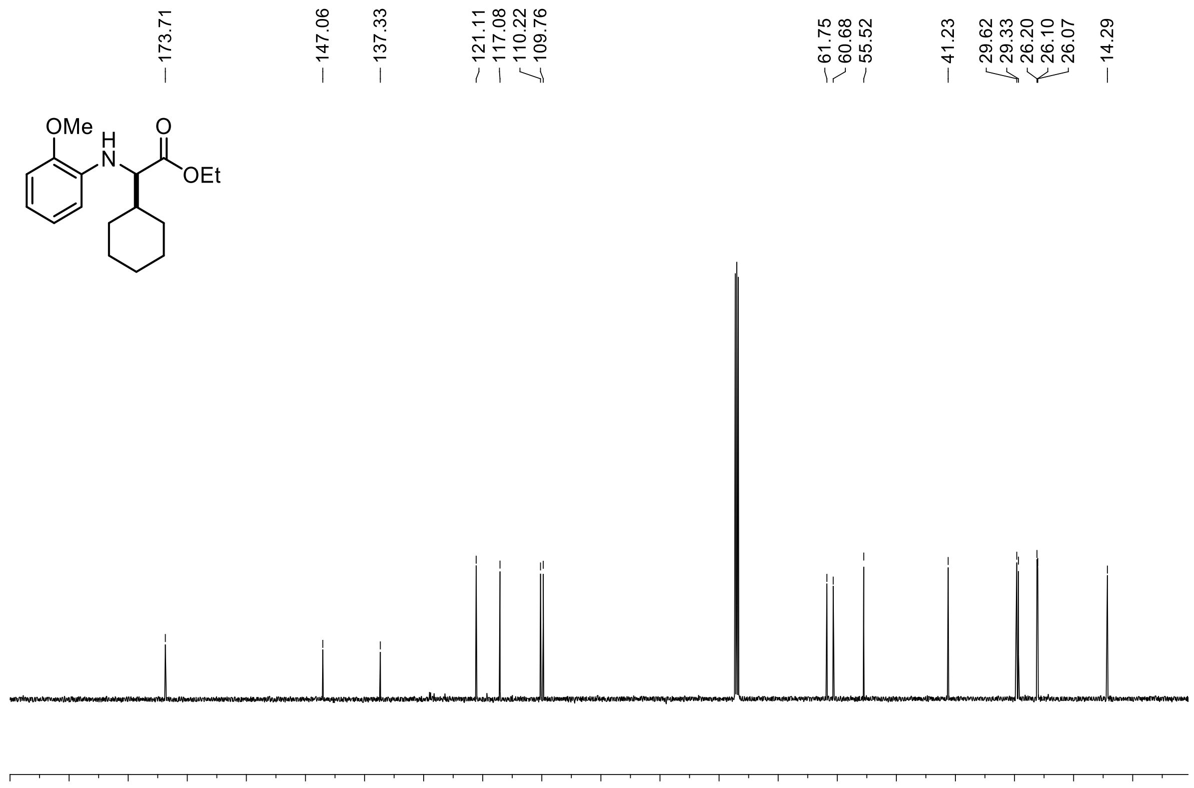

^{1}H NMR spectrum of 9 ($CDCl_3$, 400 MHz)

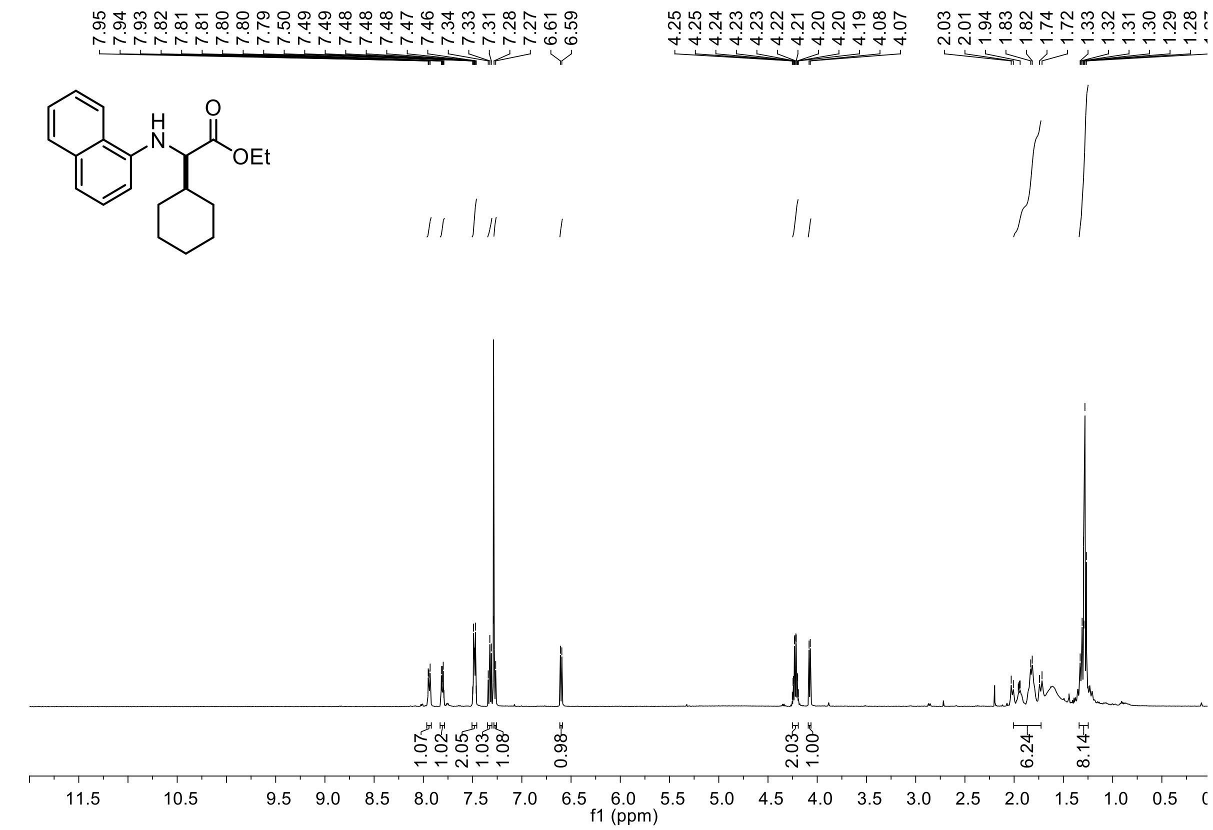

^{13}C{^{1}H} NMR spectrum of 9 ($CDCl_3$, 101 MHz)

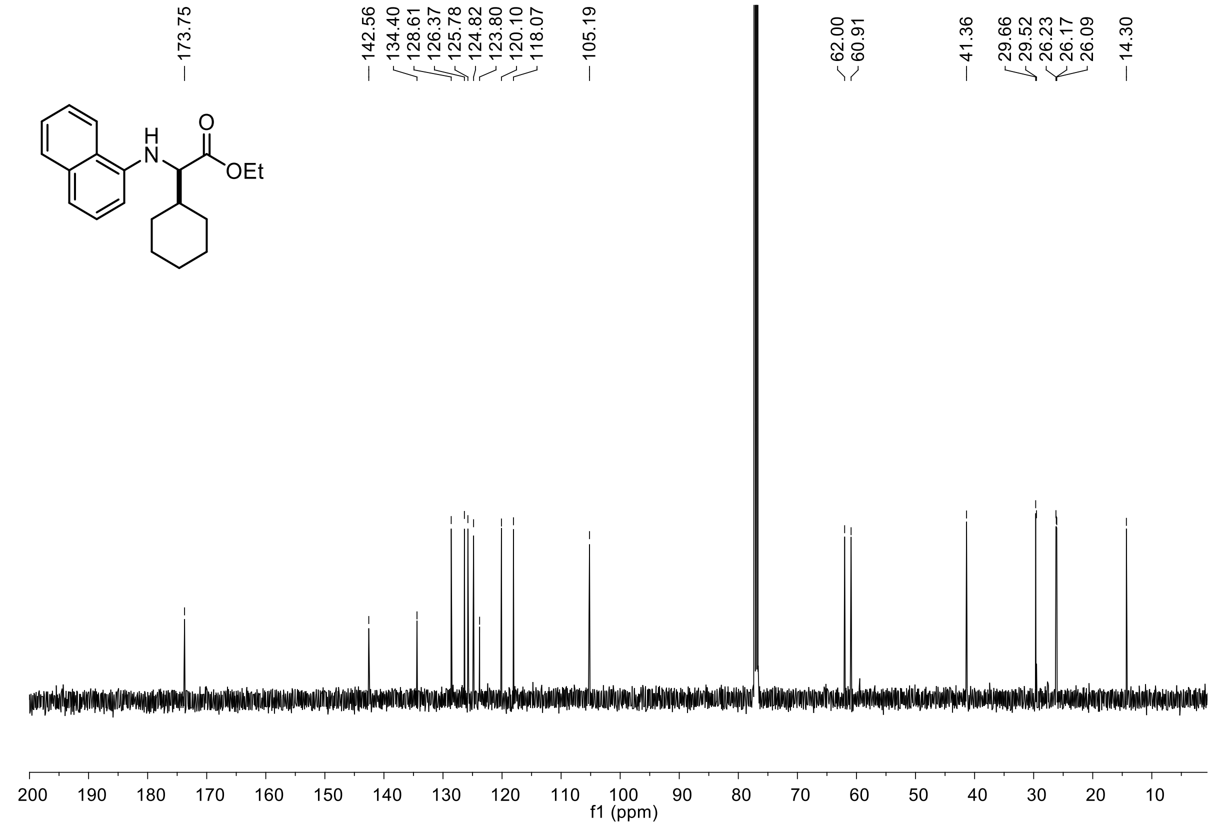

^{1}H NMR spectrum of 10 (CDCl$_3$, 500 MHz)

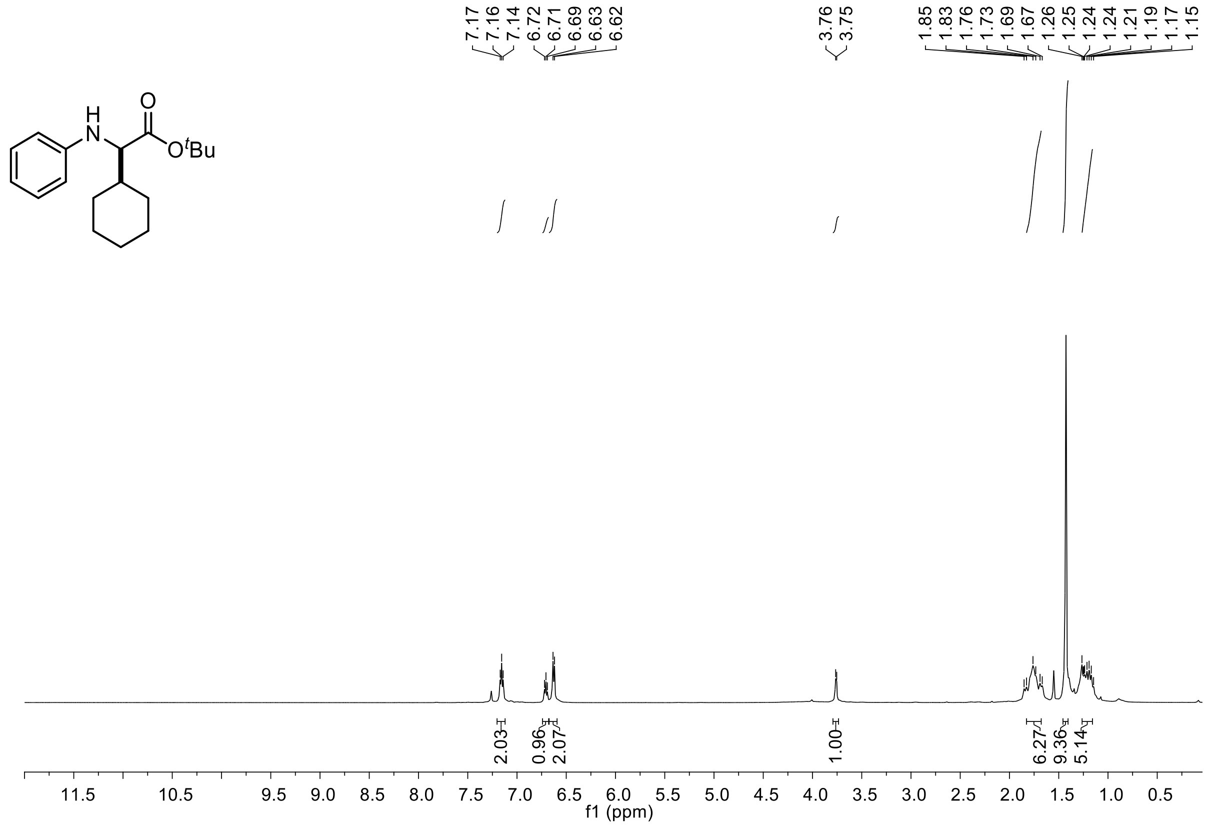

^{13}C{^{1}H} NMR spectrum of 10 (CDCl$_3$, 126 MHz)

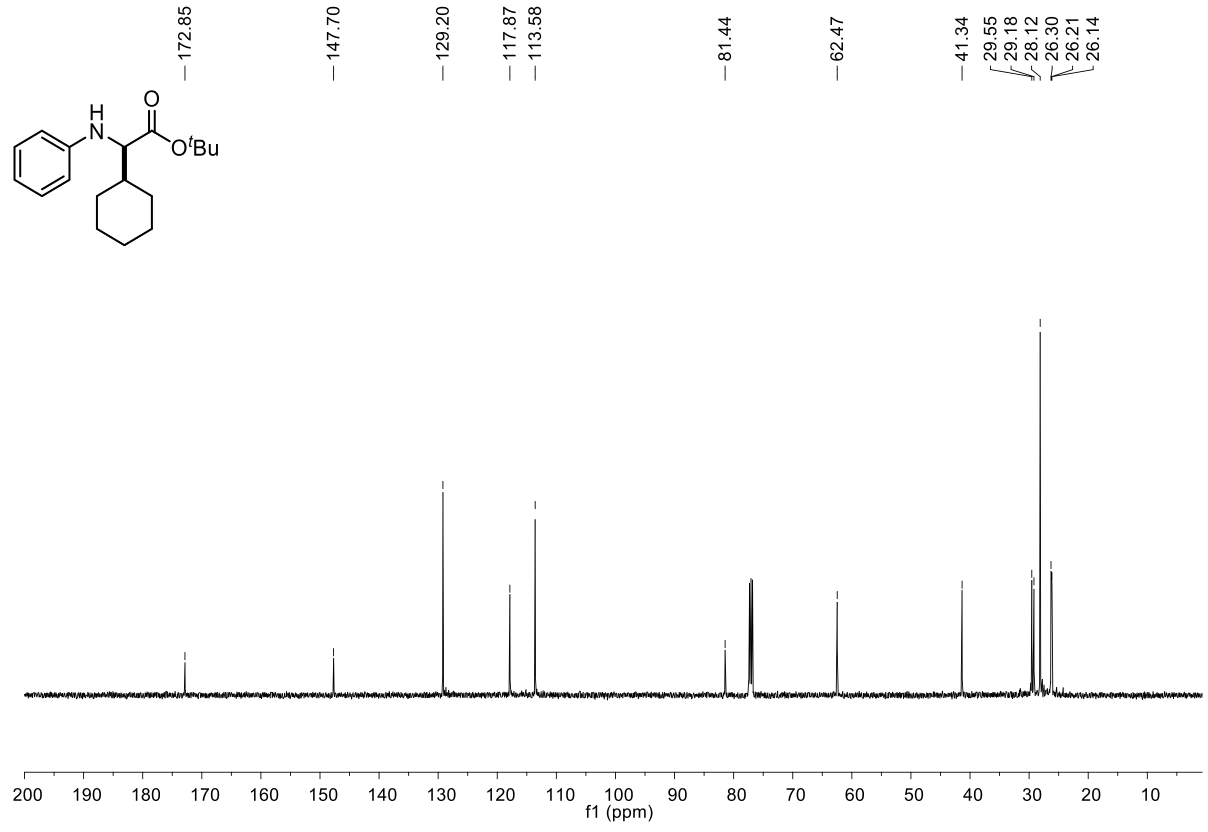

^{1}H NMR spectrum of 11 ($CDCl_3$, 500 MHz)

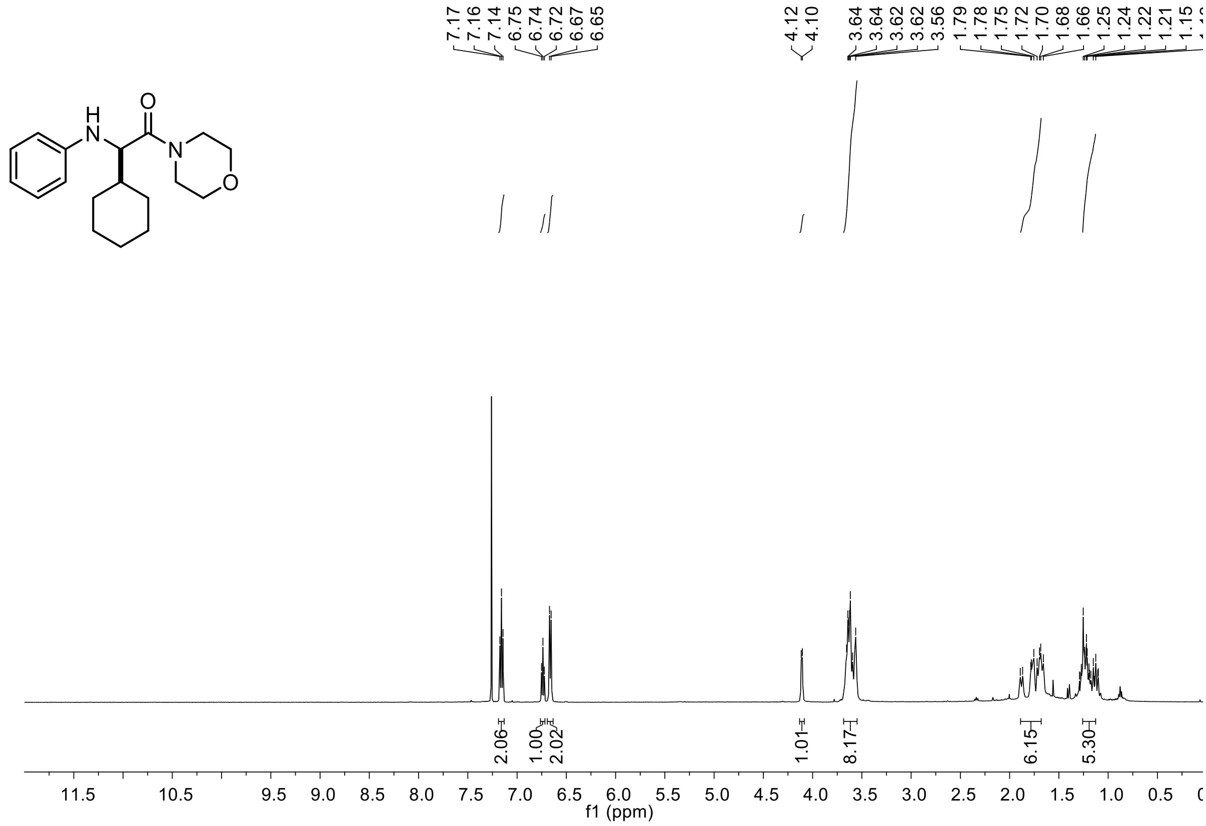

$^{13}C\{^{1}H\}$ NMR spectrum of 11 ($CDCl_3$, 126 MHz)

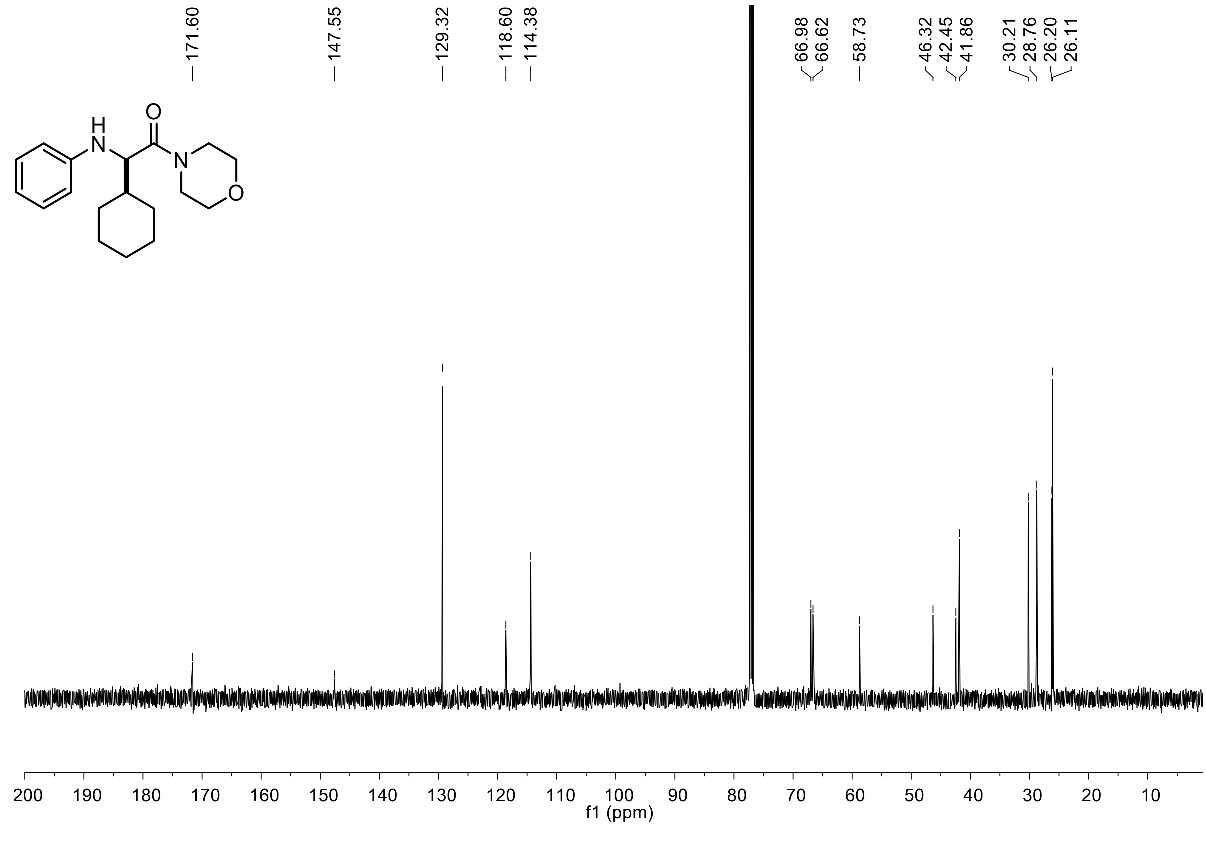

^{1}H NMR spectrum of 12 ($CDCl_3$, 500 MHz)

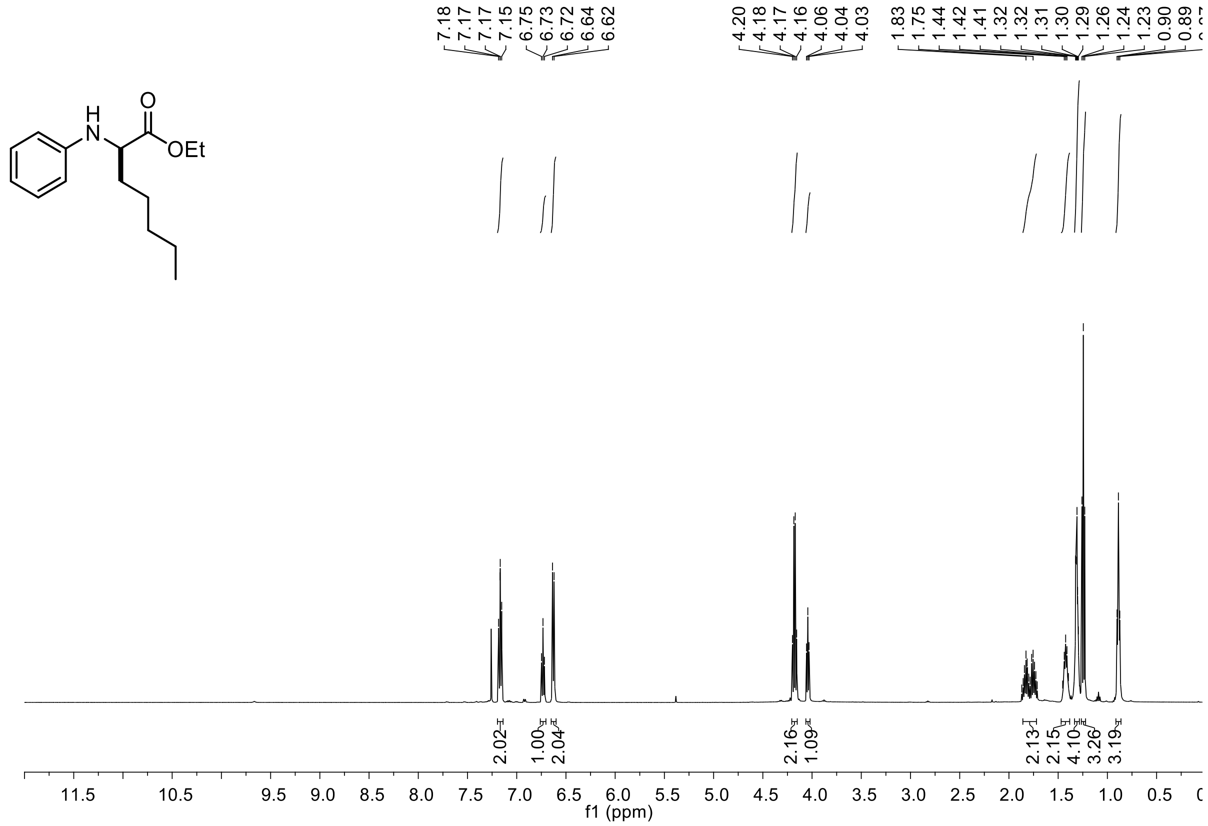

^{13}C{^{1}H} NMR spectrum of 12 ($CDCl_3$, 126 MHz)

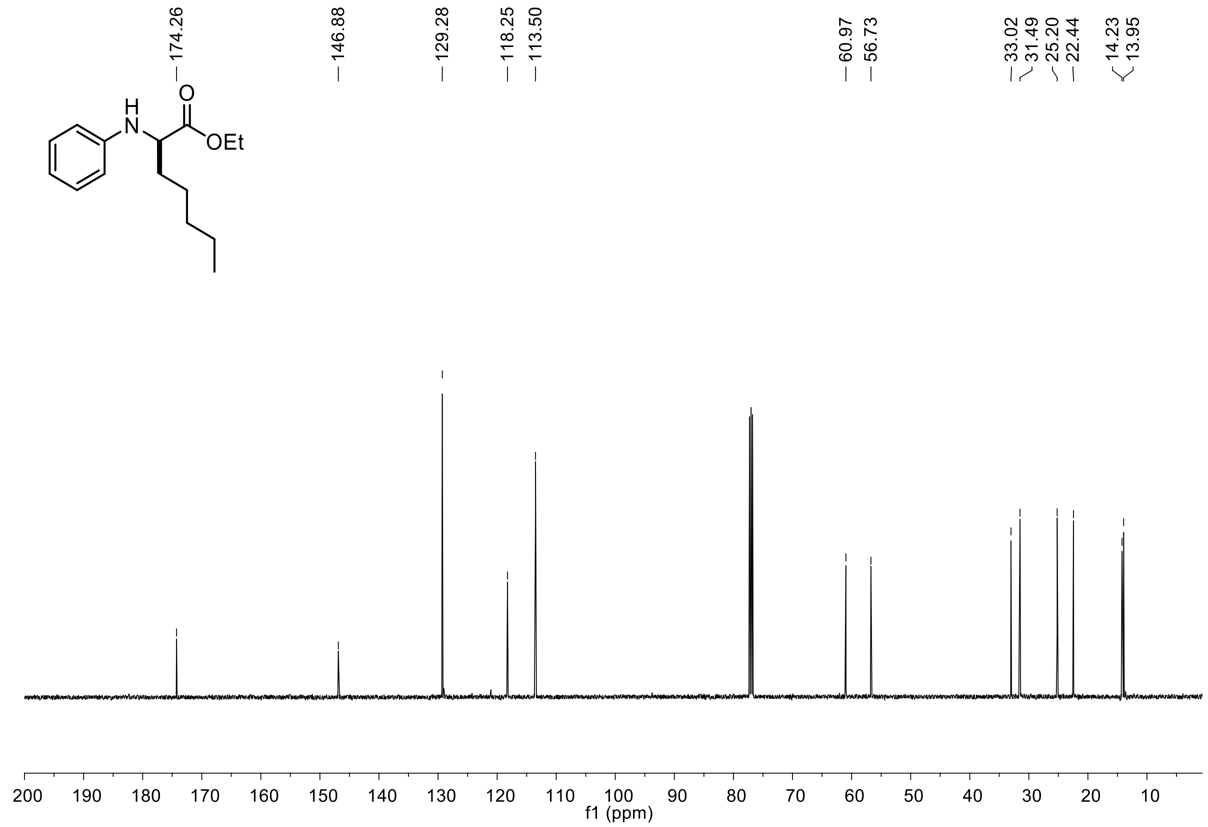

^{1}H NMR spectrum of 13 ($CDCl_3$, 400 MHz)

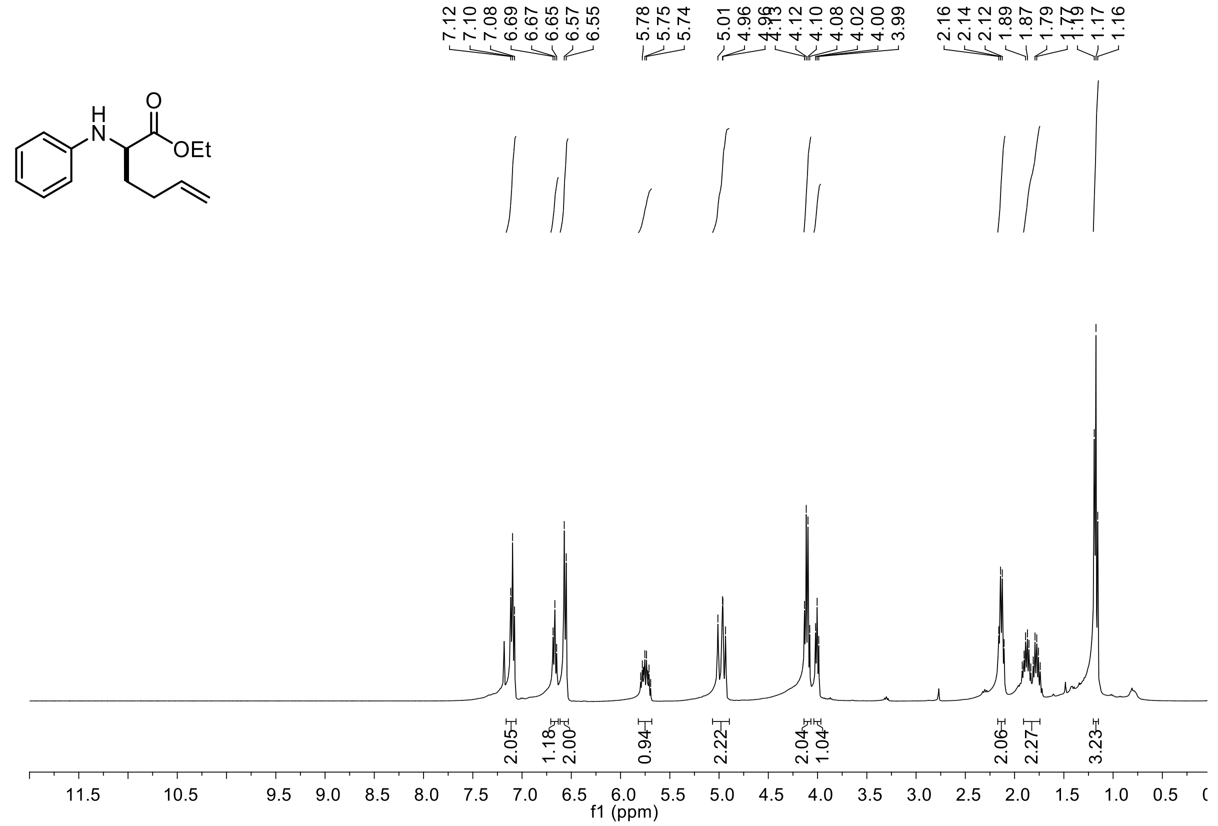

^{13}C{^{1}H} NMR spectrum of 13 ($CDCl_3$, 101 MHz)

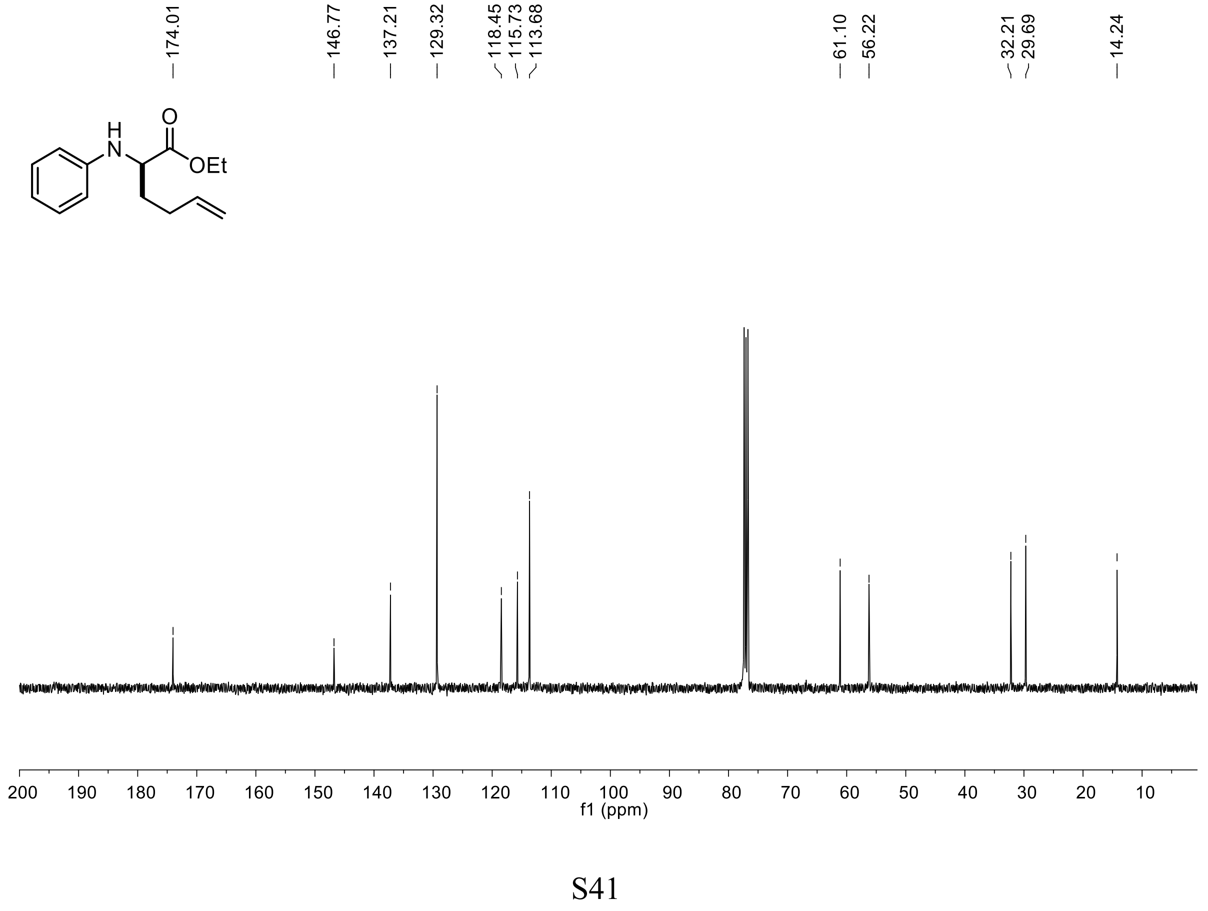

^{1}H NMR spectrum of 14 (CDCl$_3$, 500 MHz)

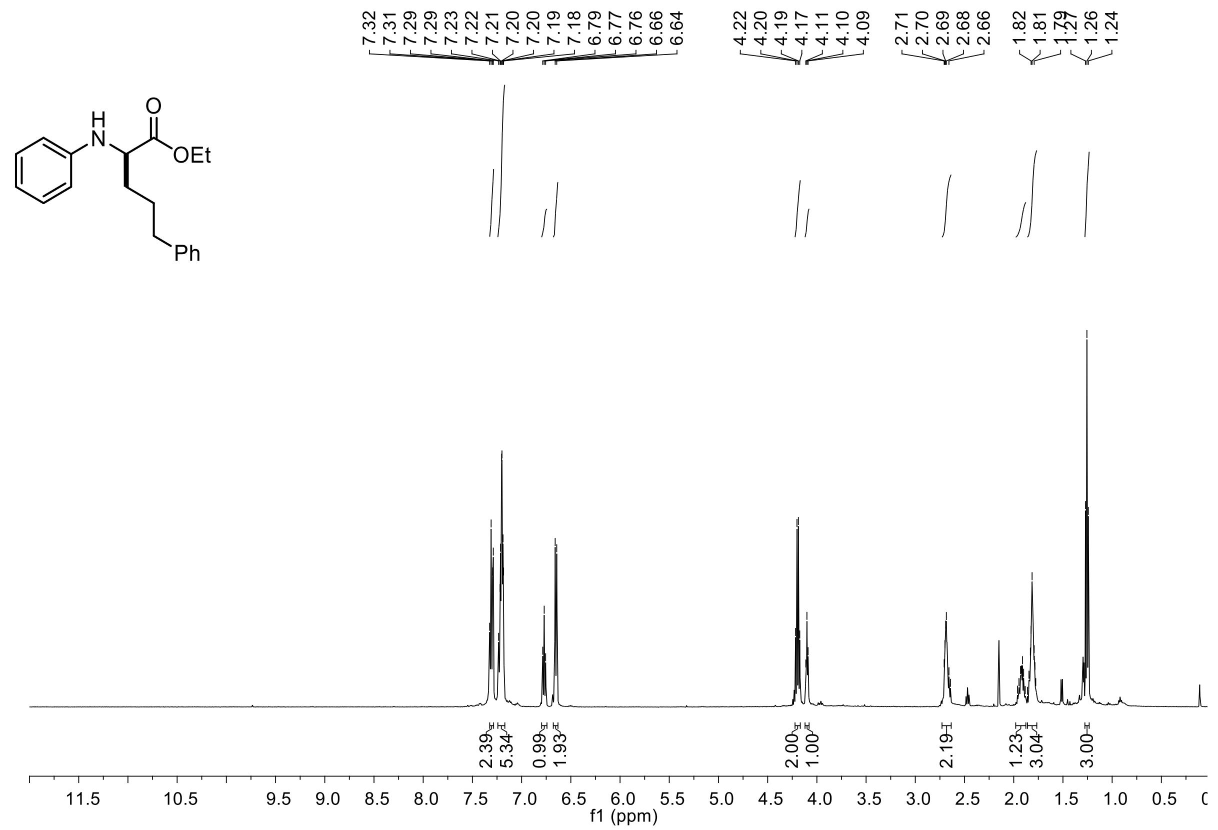

^{13}C{^{1}H} NMR spectrum of 14 (CDCl$_3$, 126 MHz)

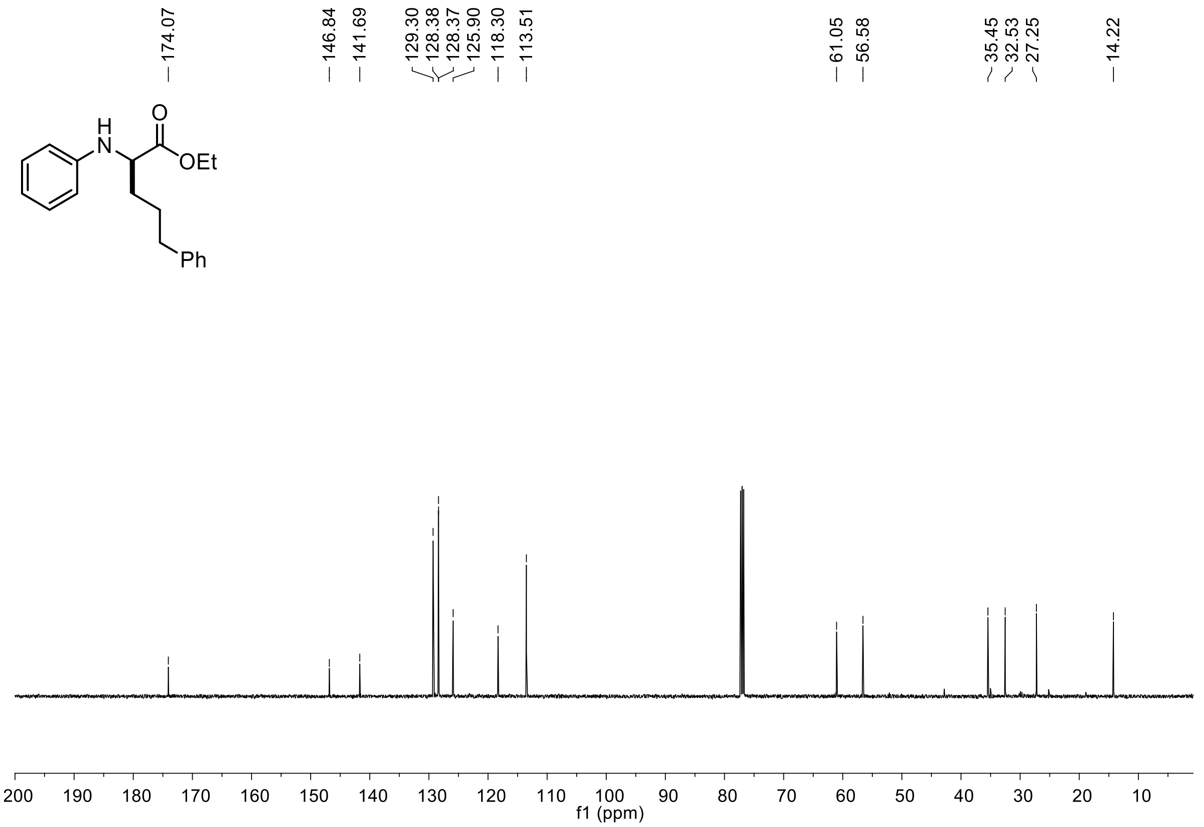

^{1}H NMR spectrum of 15 ($CDCl_3$, 500 MHz)

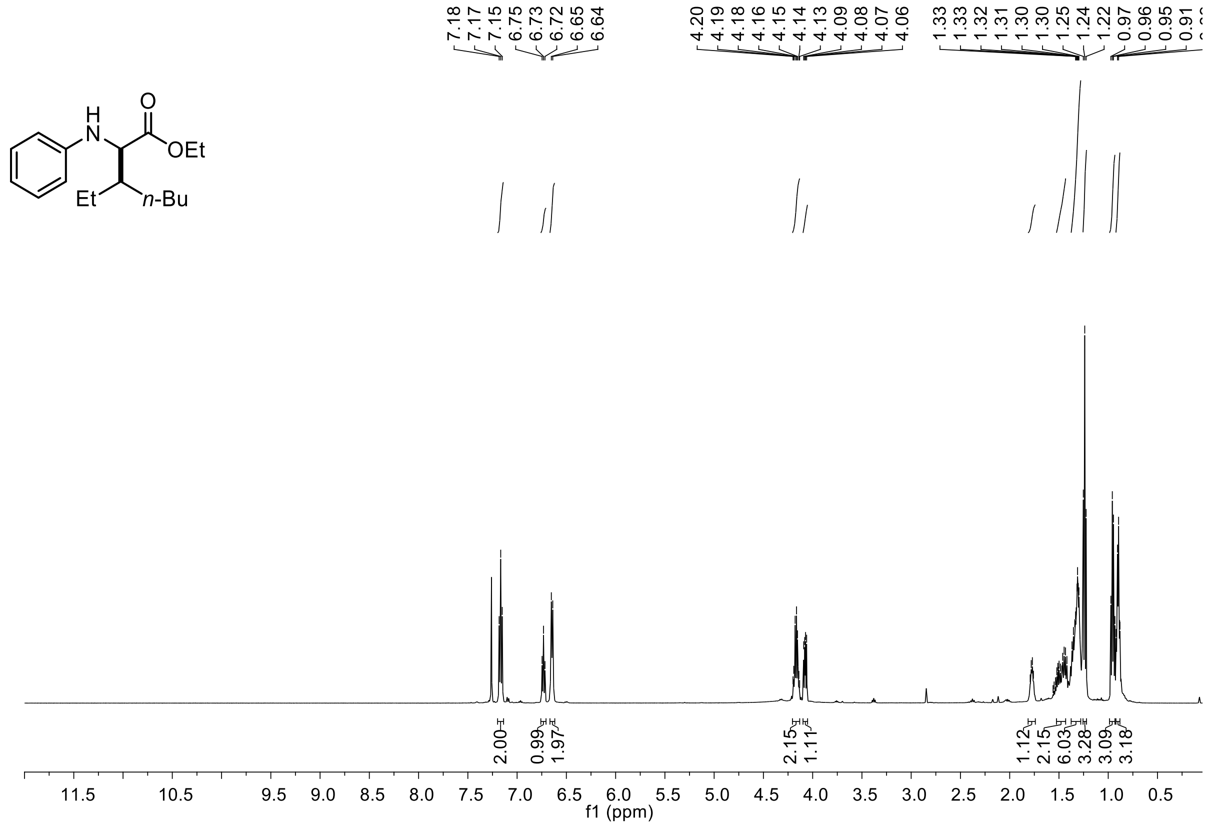

^{13}C{^{1}H} NMR spectrum of 15 ($CDCl_3$, 126 MHz)

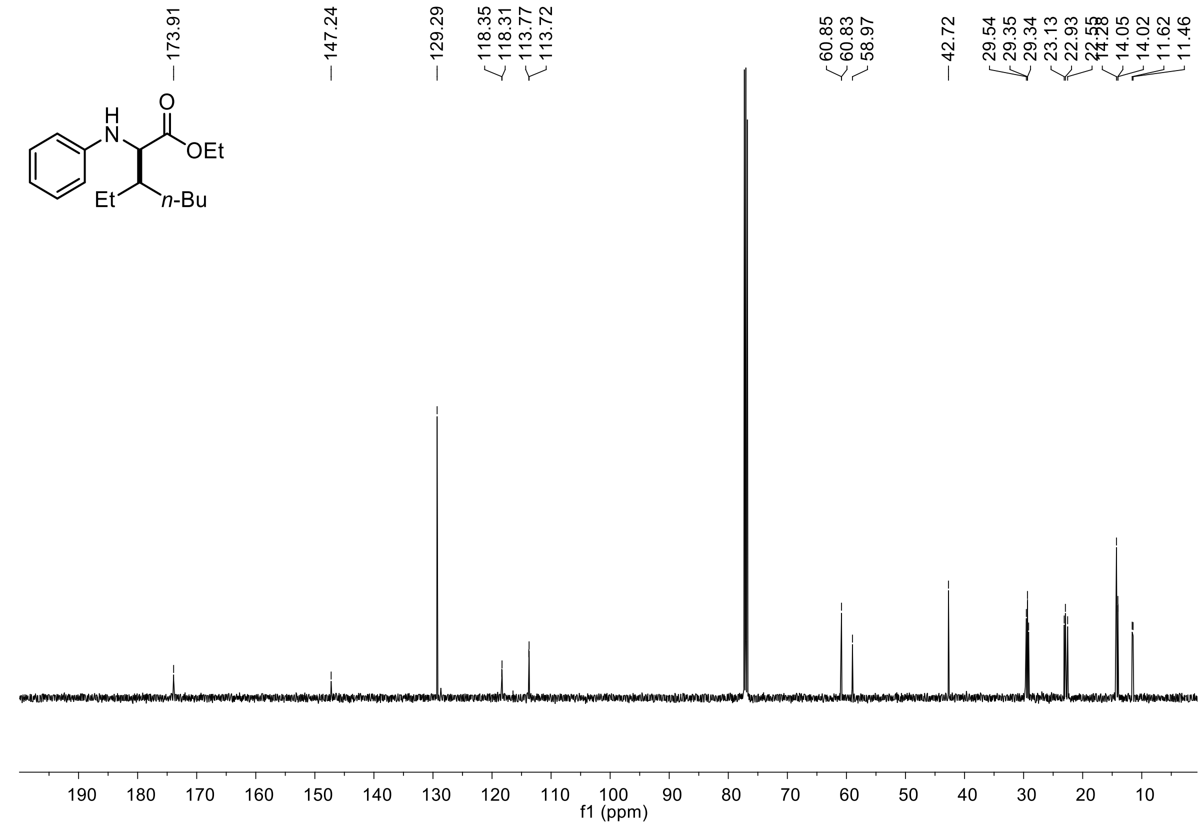

^{1}H NMR spectrum of 16 ($CDCl_3$, 500 MHz)

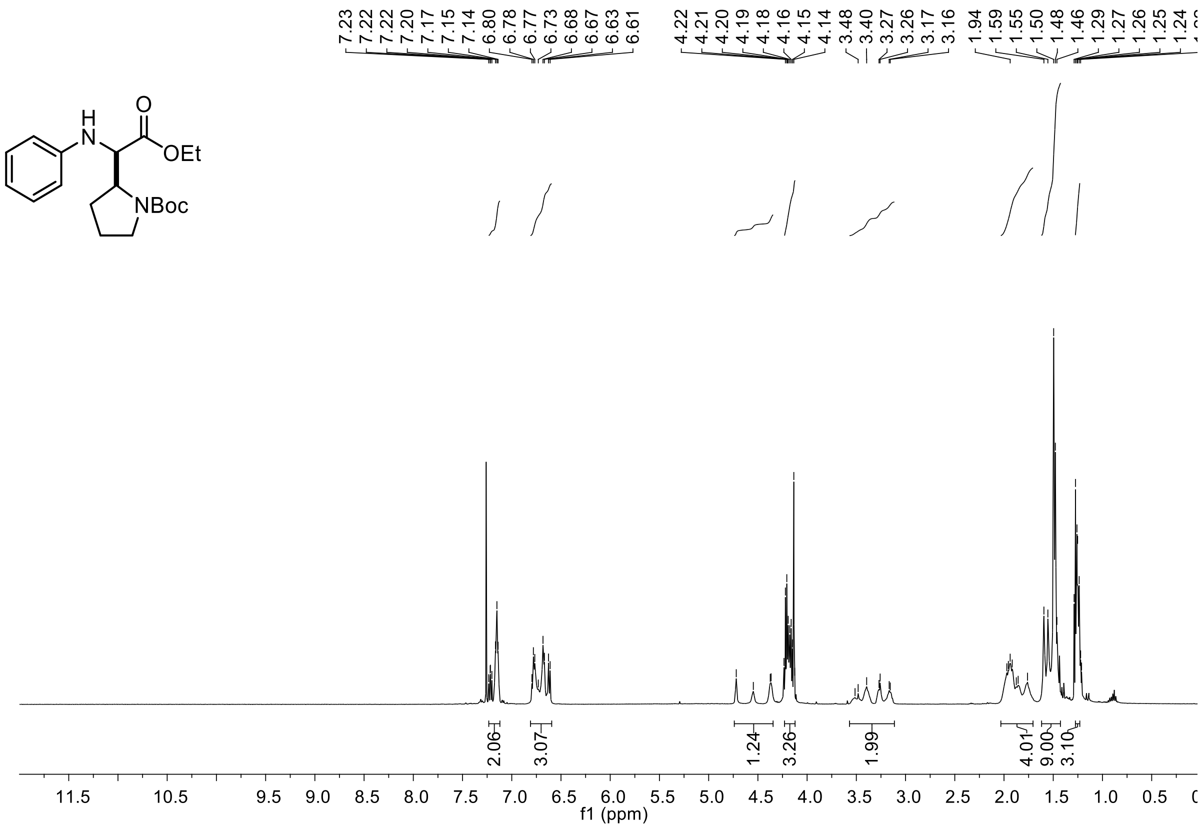

^{13}C{^{1}H} NMR spectrum of 16 ($CDCl_3$, 126 MHz)

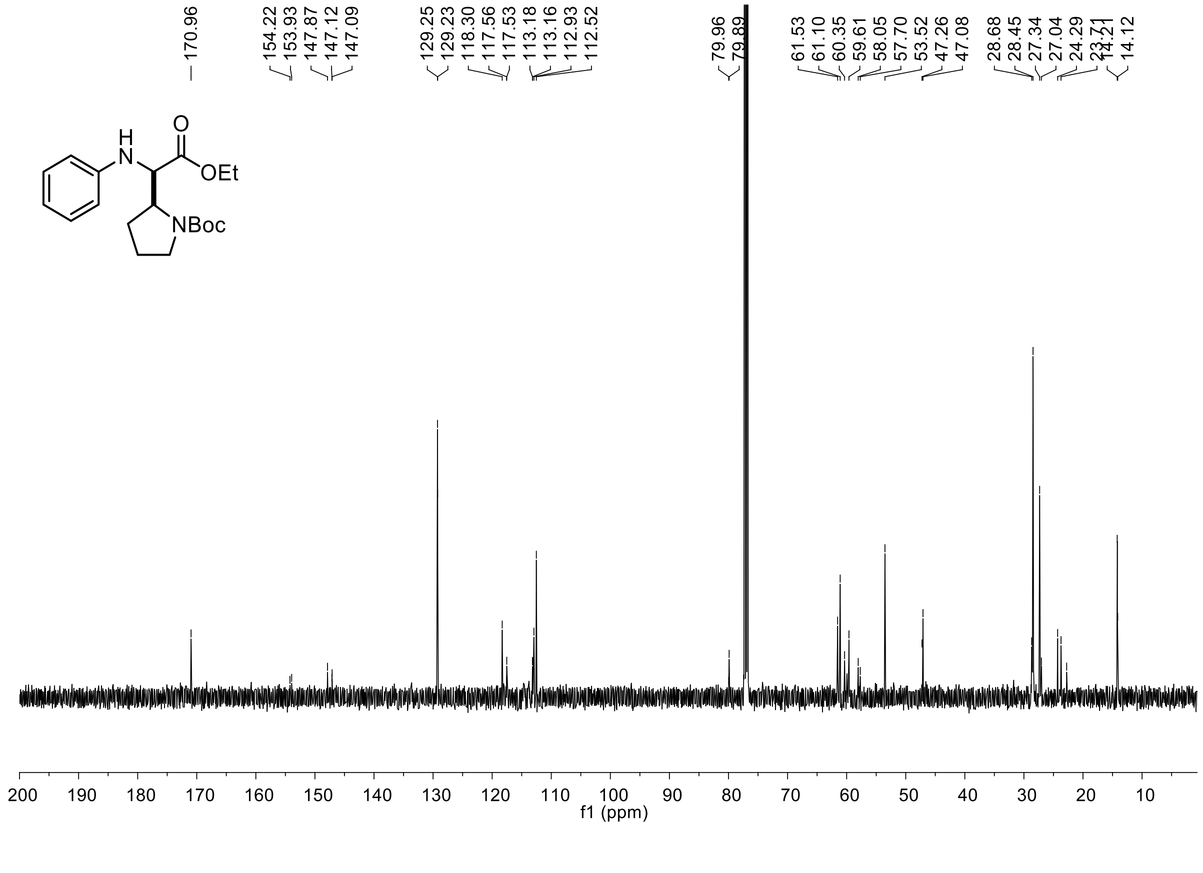

^{1}H NMR spectrum of 17 ($CDCl_3$, 500 MHz)

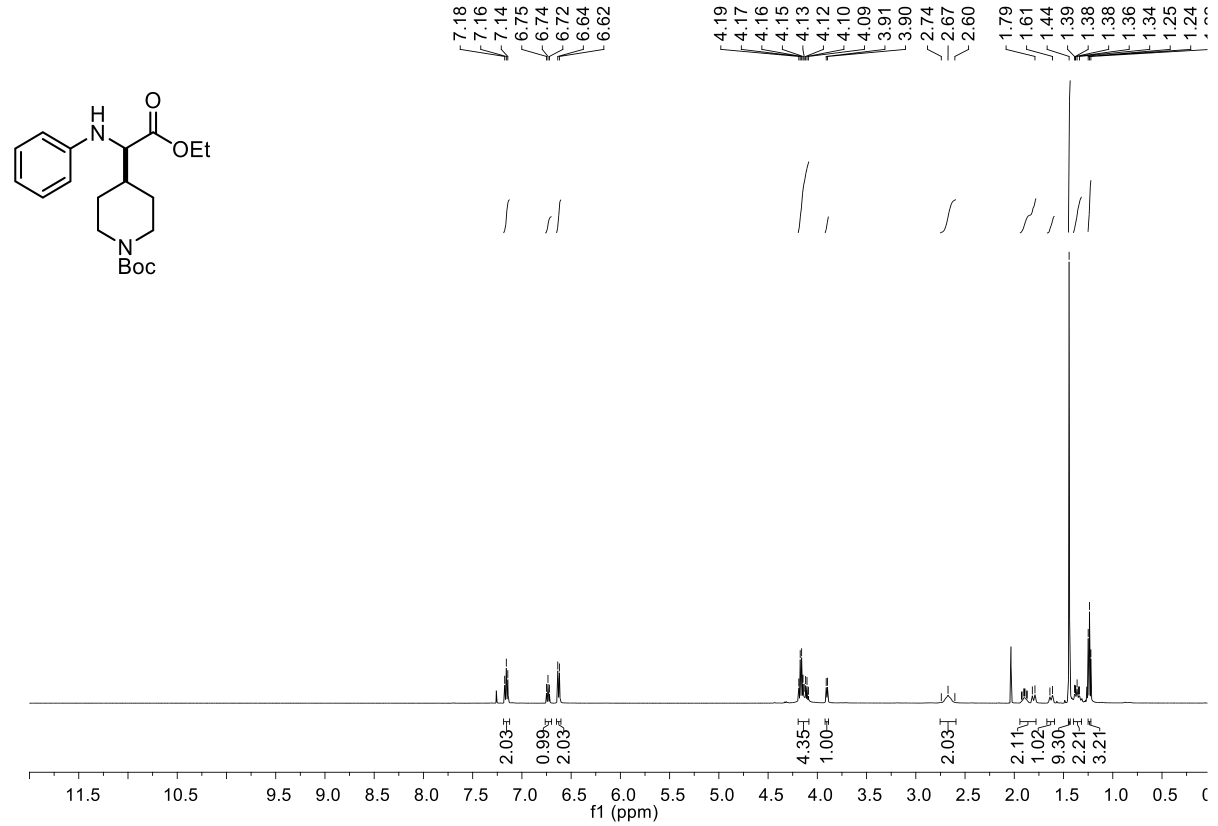

^{13}C{^{1}H} NMR spectrum of 17 ($CDCl_3$, 126 MHz)

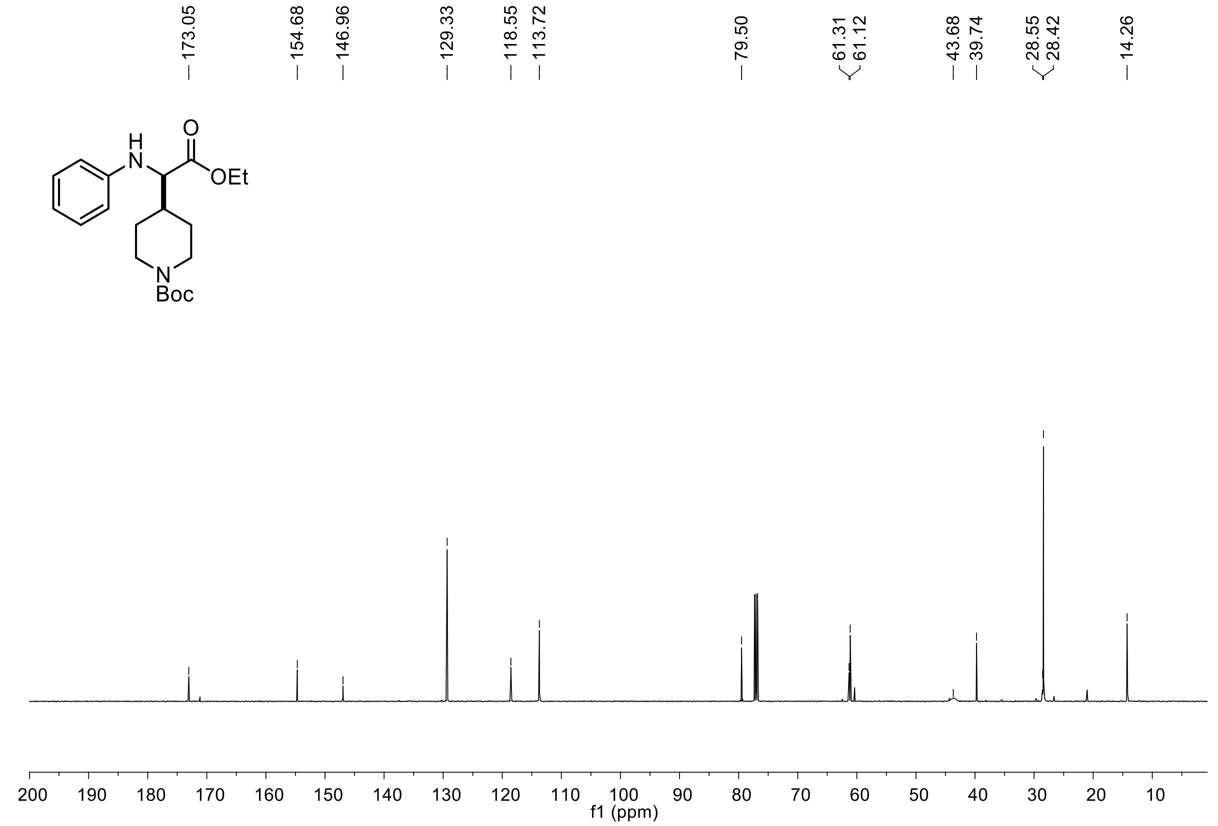

^{1}H NMR spectrum of 18 ($CDCl_3$, 500 MHz)

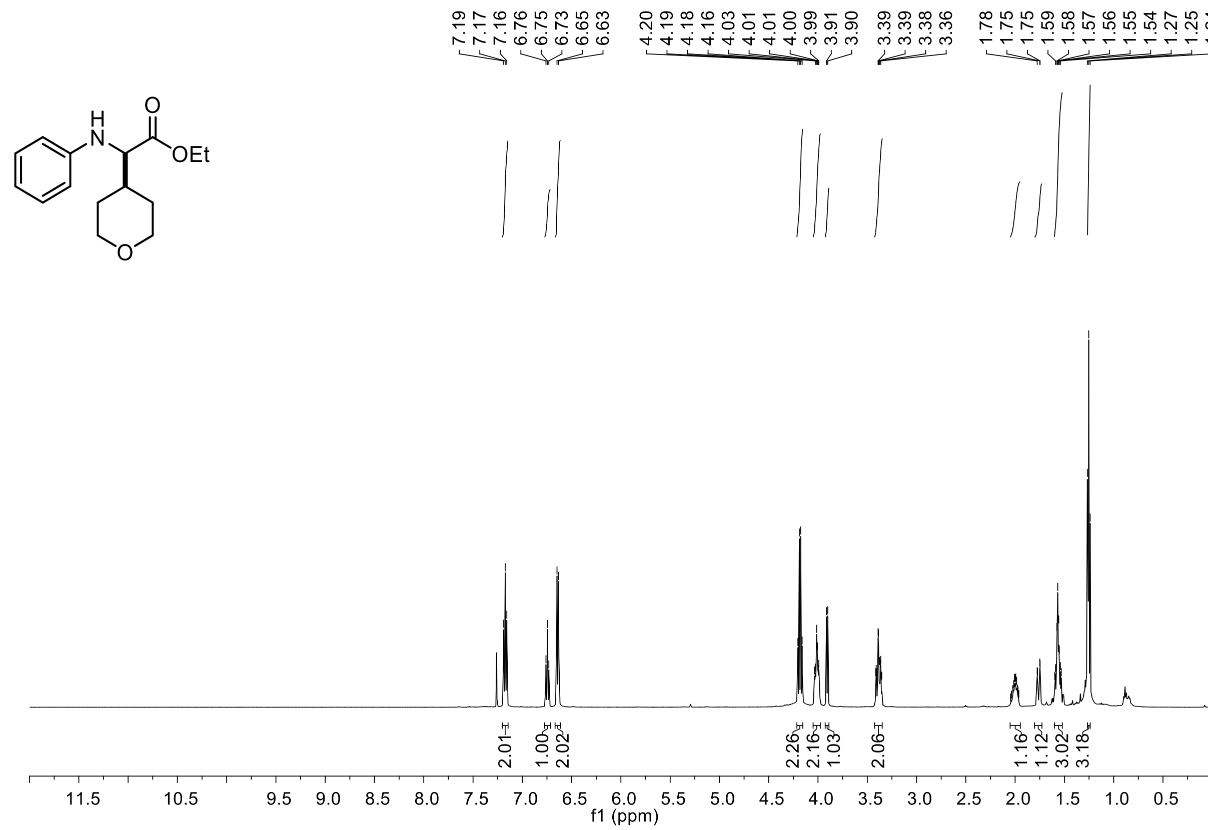

^{13}C{^{1}H} NMR spectrum of 18 ($CDCl_3$, 126 MHz)

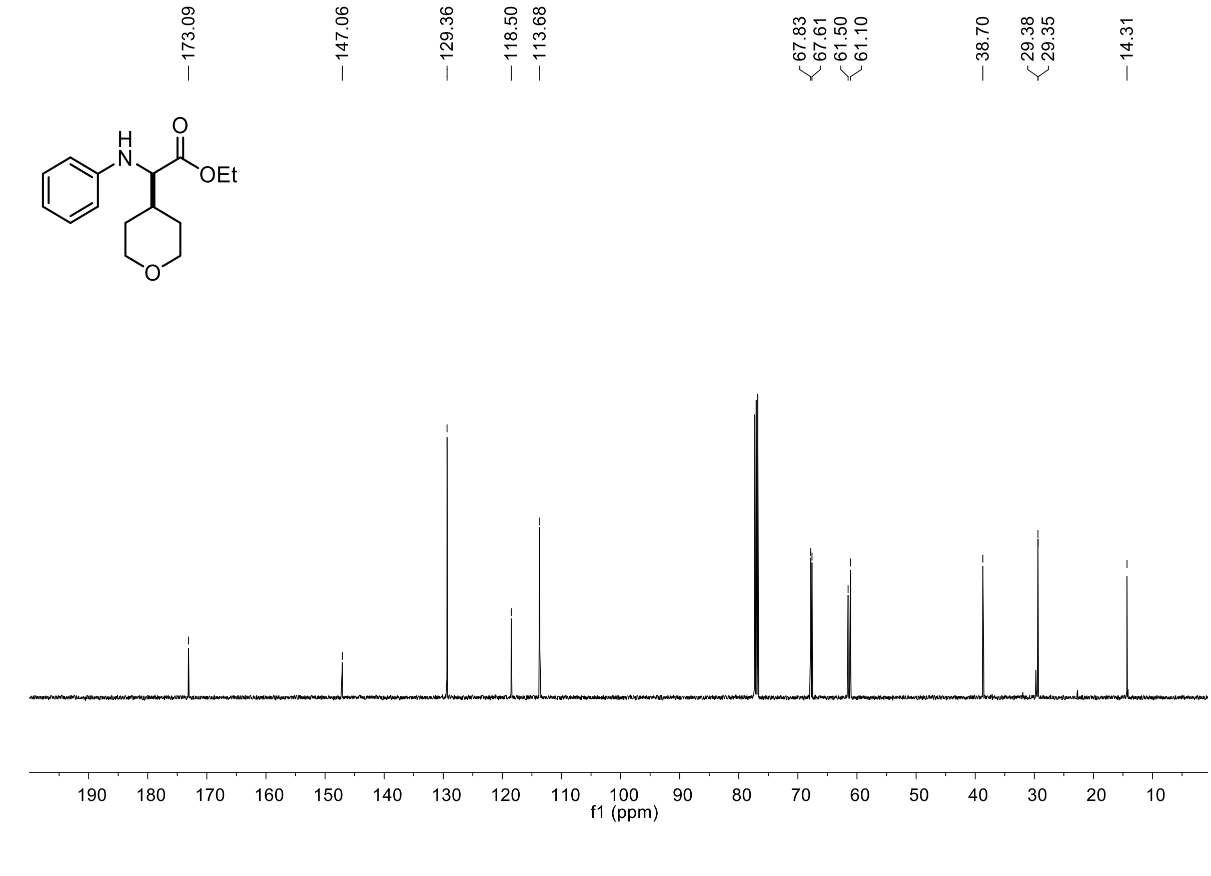

^{1}H NMR spectrum of 19 ($CDCl_3$, 400 MHz)

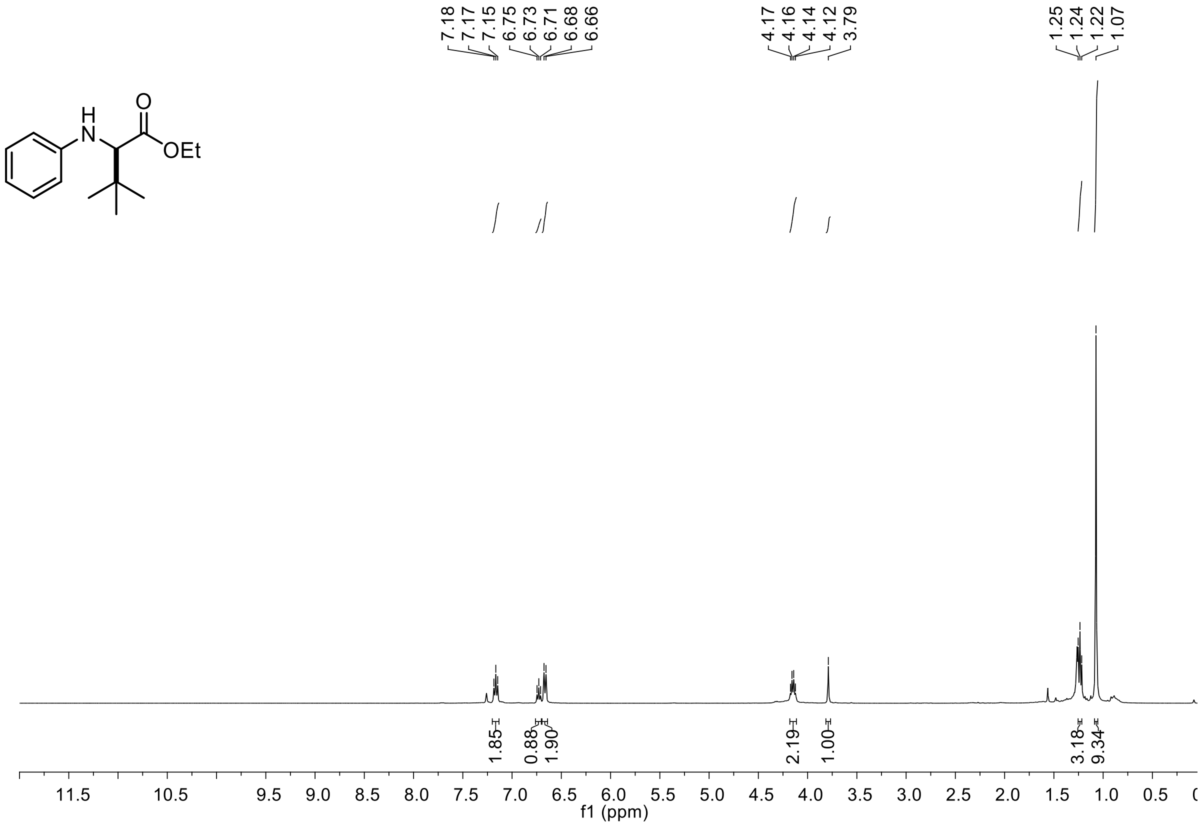

^{13}C{^{1}H} NMR spectrum of 19 ($CDCl_3$, 101 MHz)

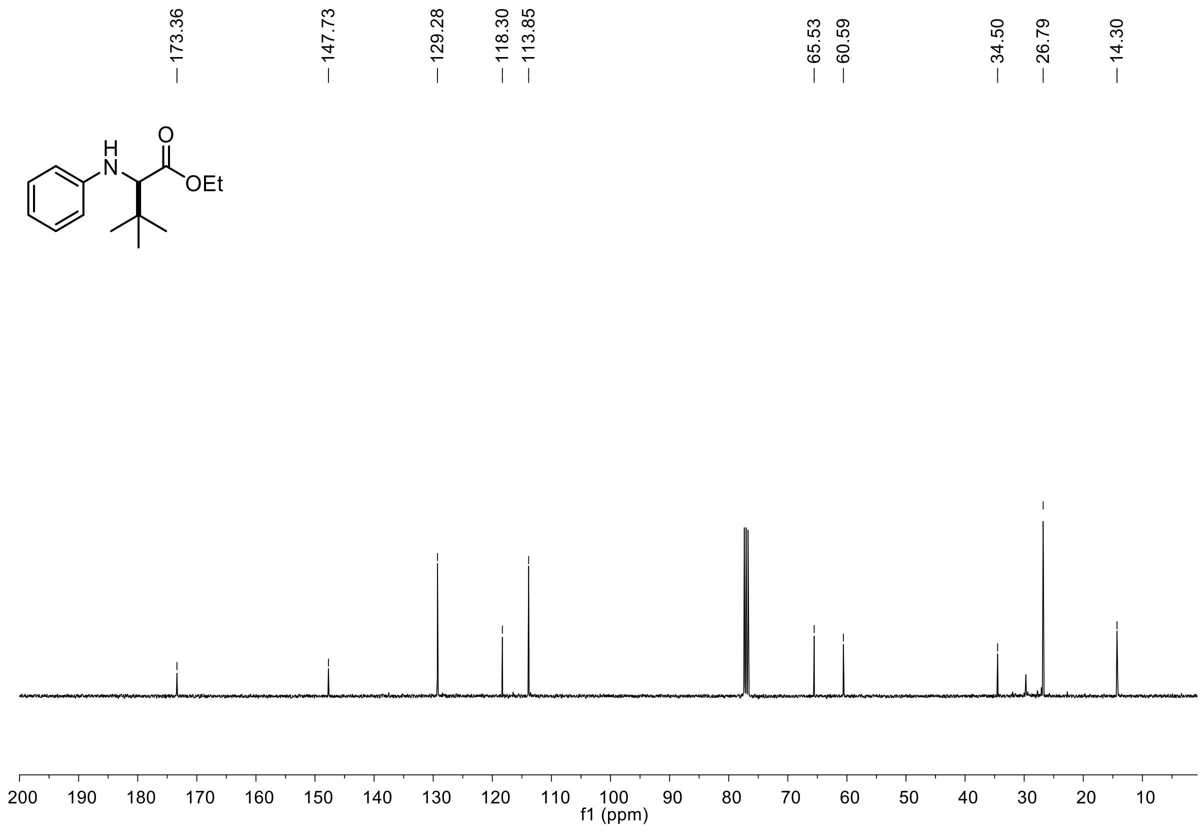

^{1}H NMR spectrum of 20 ($CDCl_3$, 500 MHz)

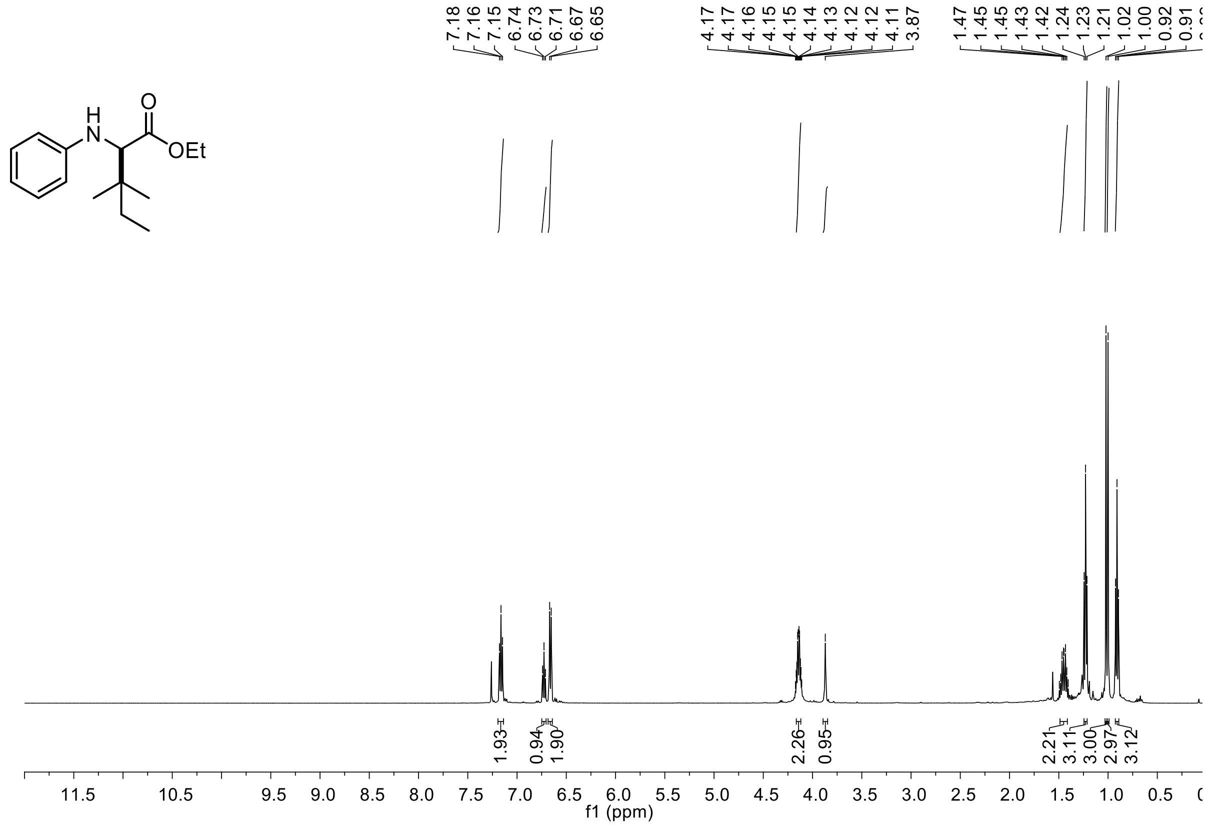

^{13}C{^{1}H} NMR spectrum of 20 ($CDCl_3$, 126 MHz)

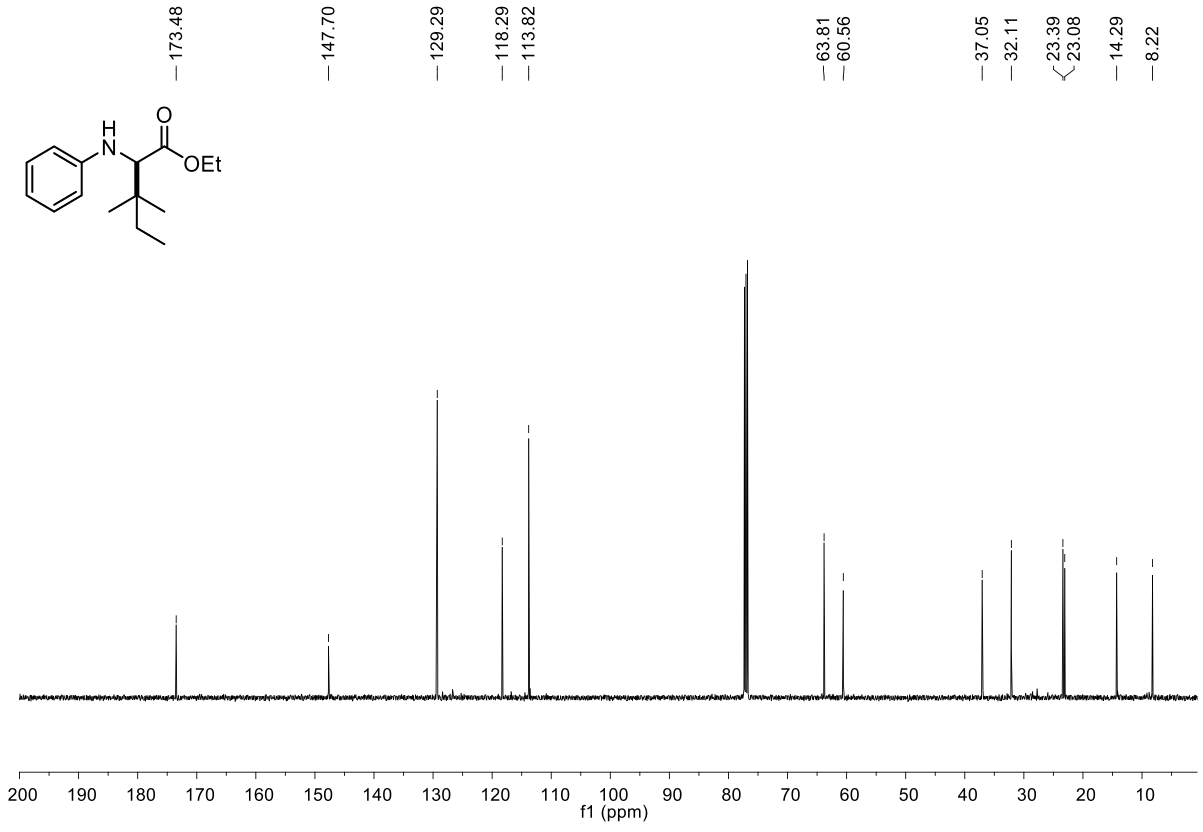

^{1}H NMR spectrum of 21 (CDCl$_3$, 400 MHz)

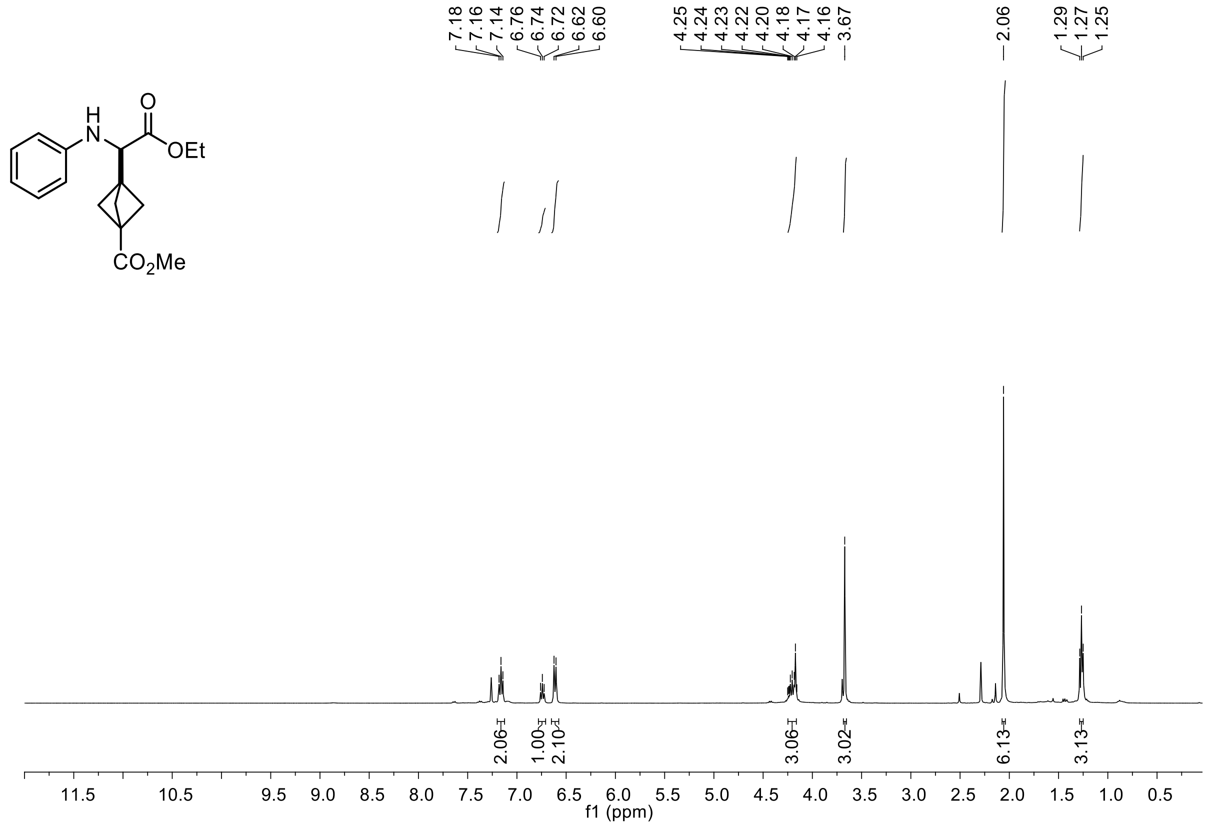

^{13}C{^{1}H} NMR spectrum of 21 (CDCl$_3$, 101 MHz)

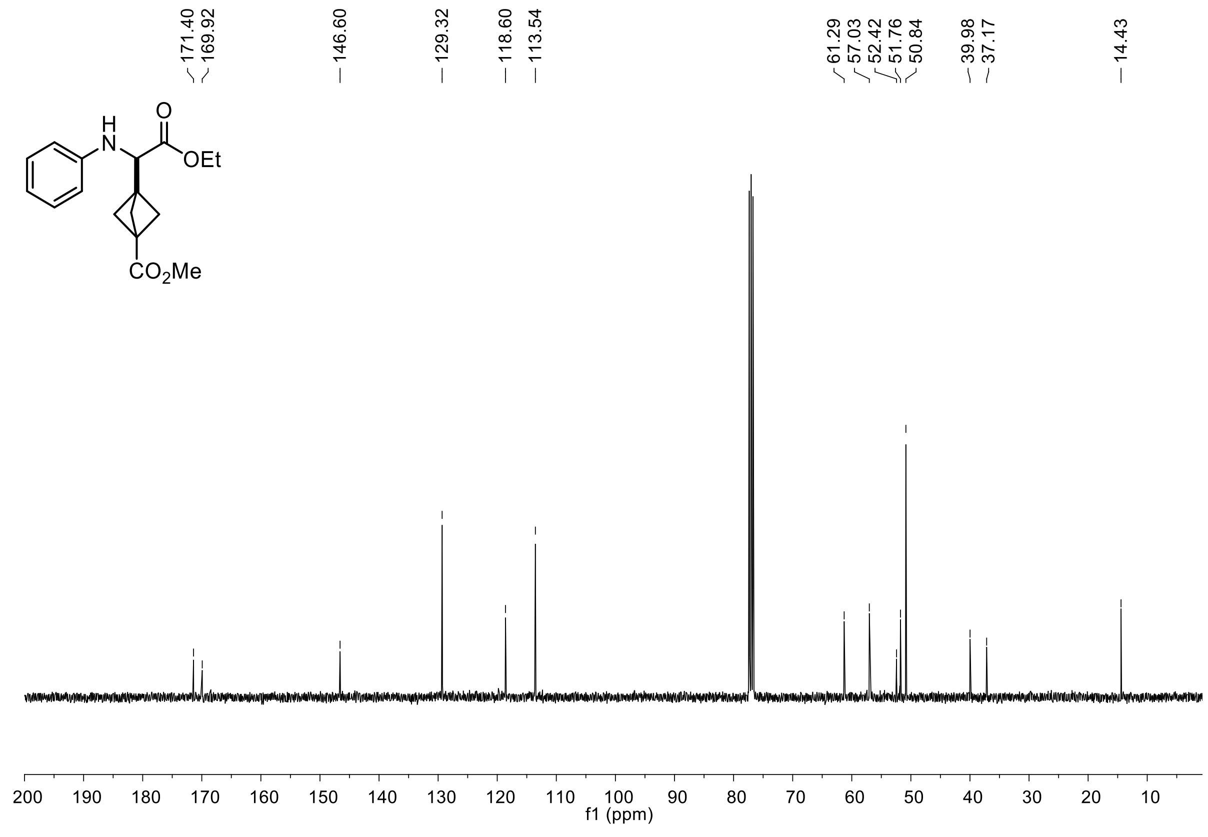

^{1}H NMR spectrum of 22 ($CDCl_3$, 500 MHz)

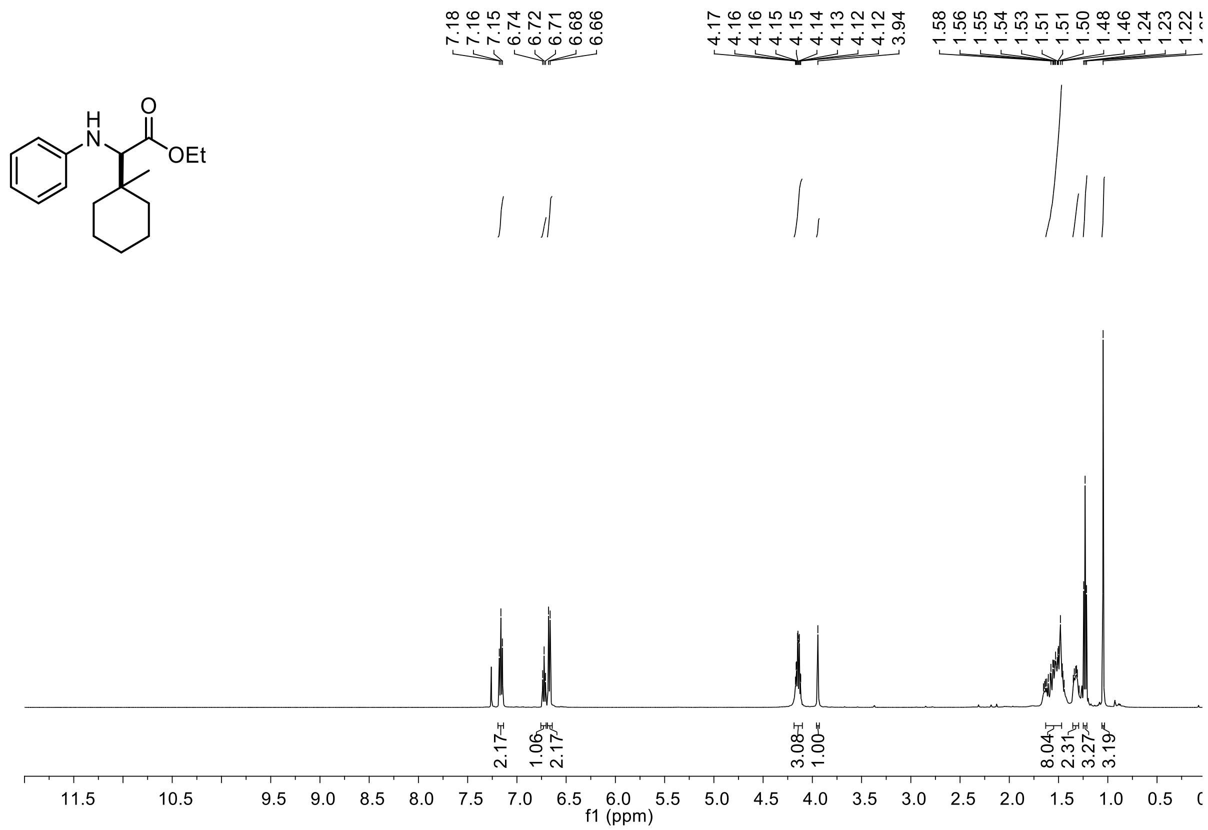

^{13}C{^{1}H} NMR spectrum of 22 ($CDCl_3$, 126 MHz)

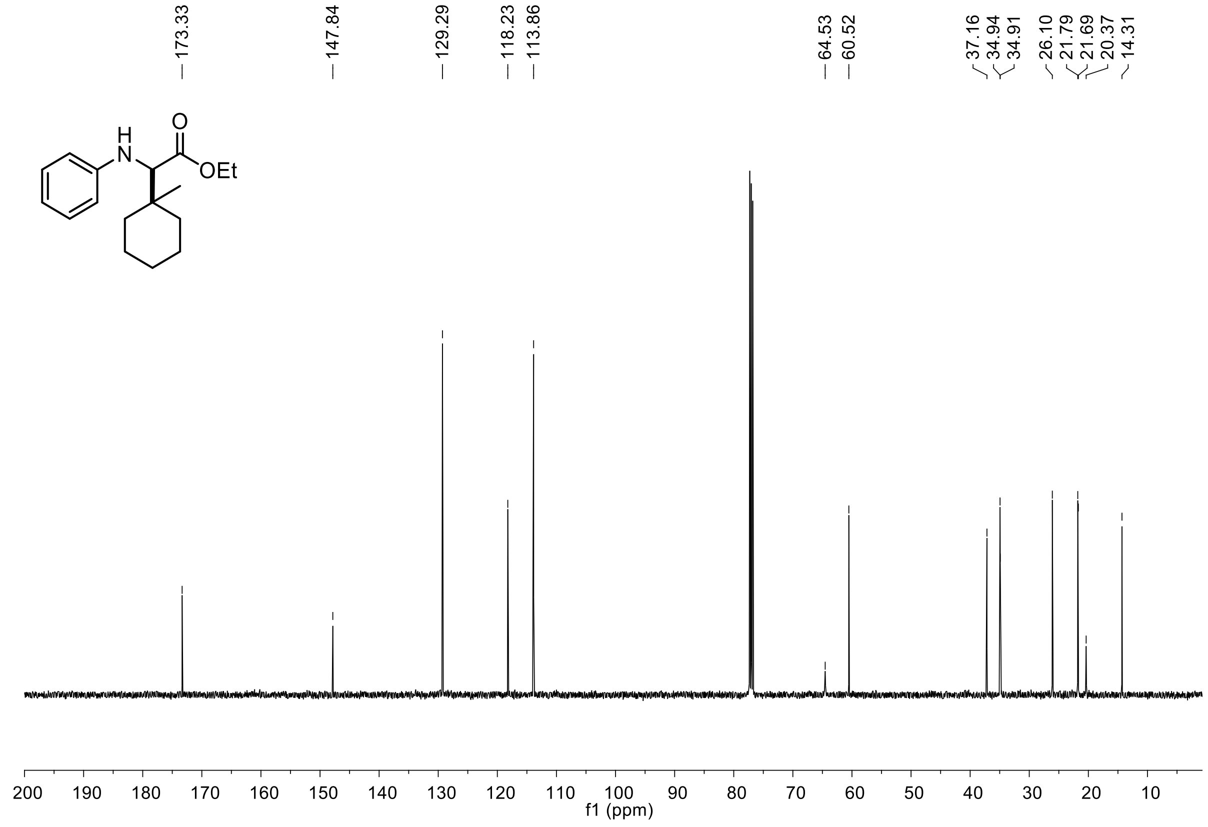

^{1}H NMR spectrum of 23 ($CDCl_3$, 500 MHz)

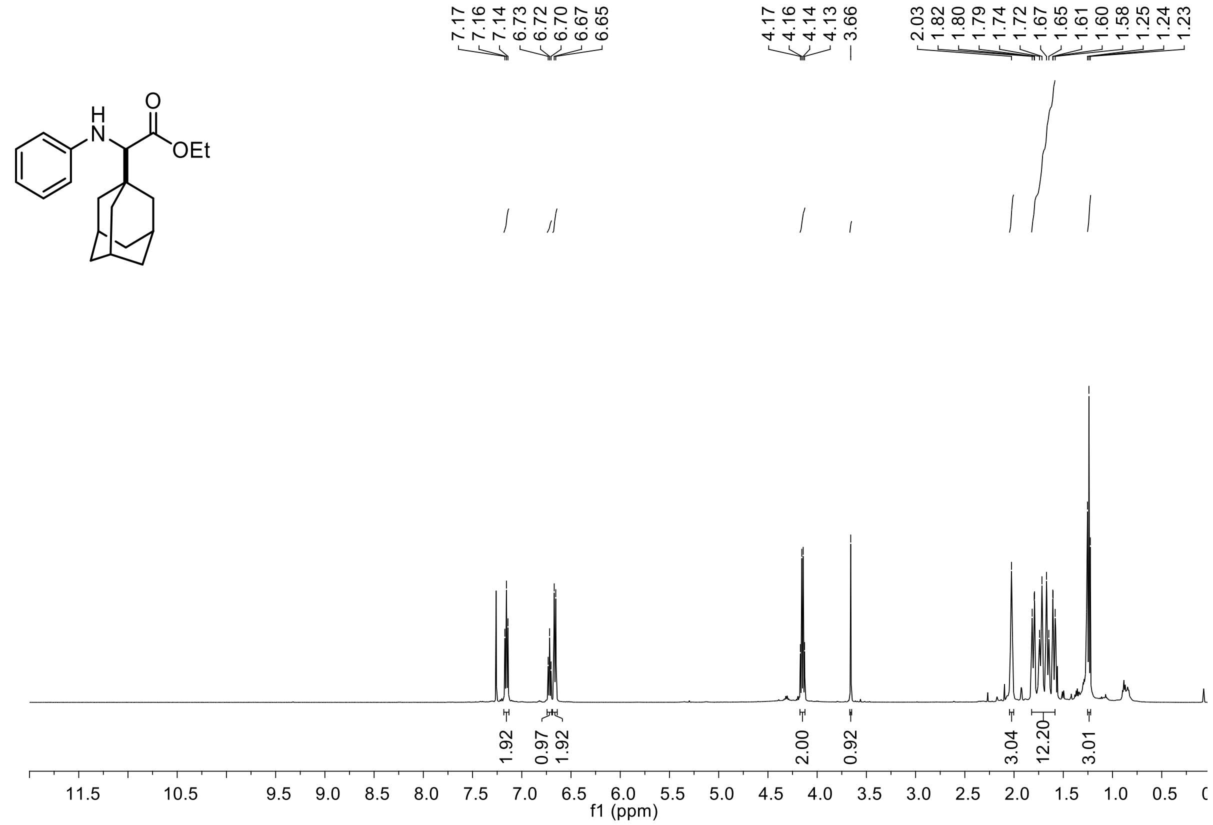

^{13}C{^{1}H} NMR spectrum of 23 ($CDCl_3$, 126 MHz)

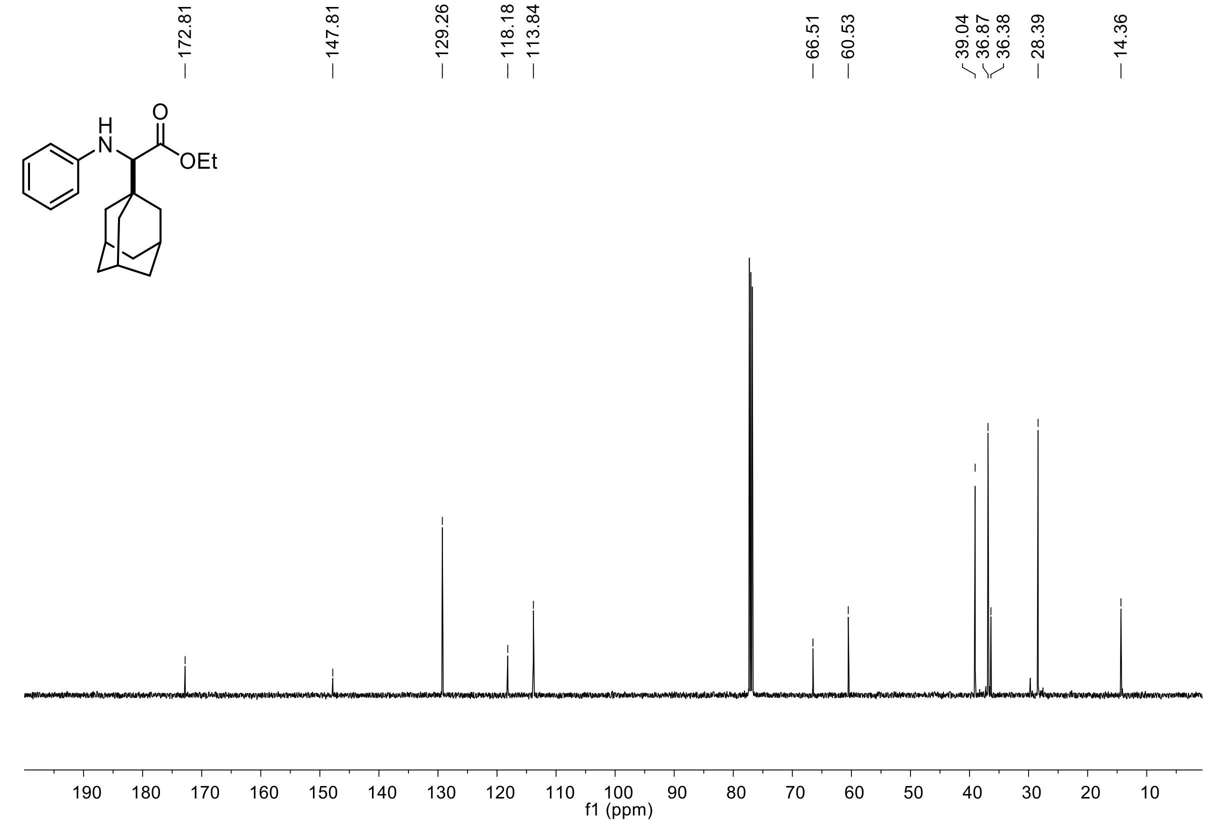

^{1}H NMR spectrum of 24 ($CDCl_3$, 500 MHz)

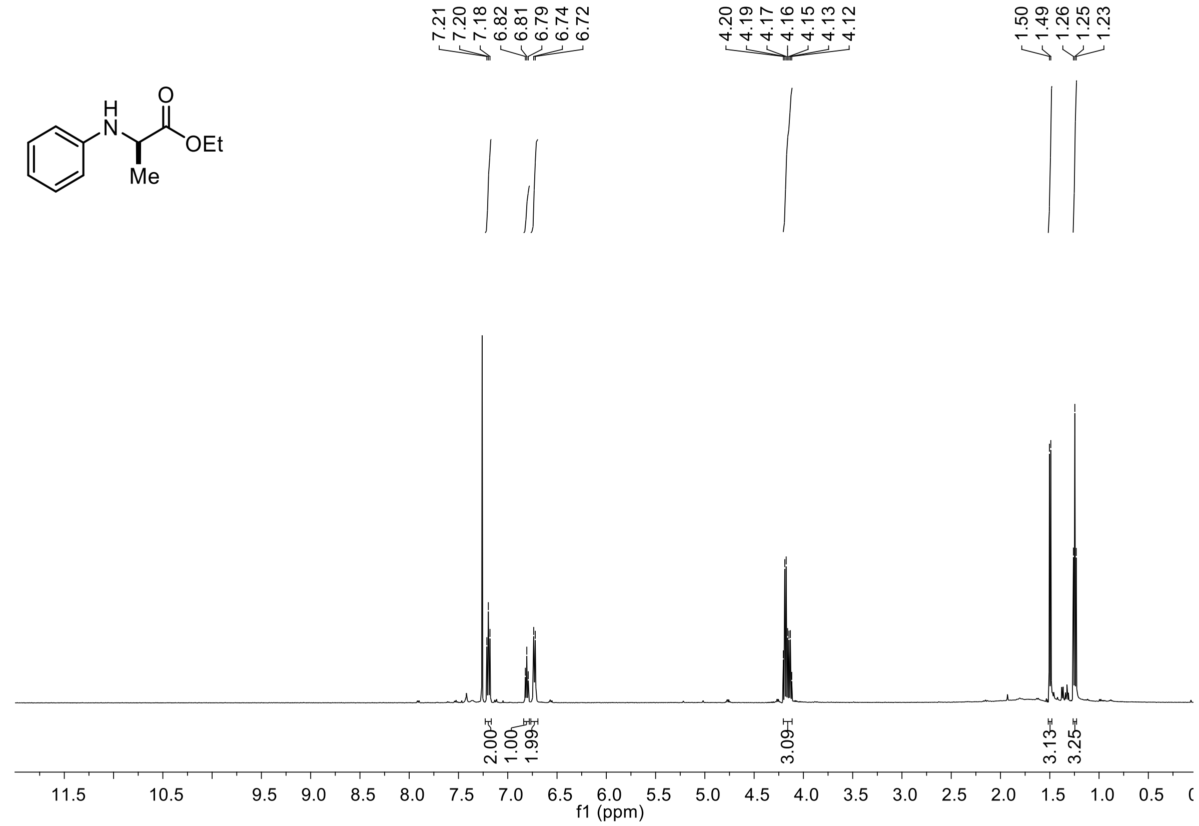

^{13}C{^{1}H} NMR spectrum of 24 ($CDCl_3$, 126 MHz)

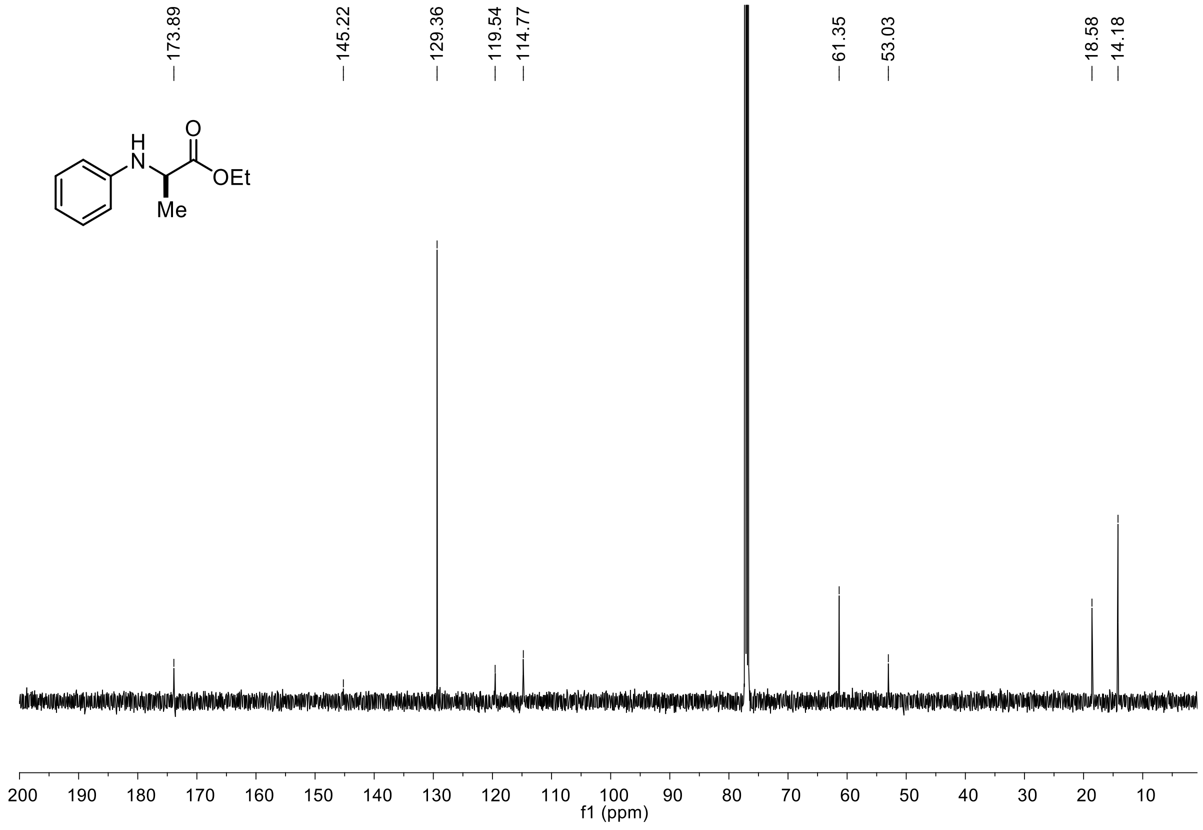

^{1}H NMR spectrum of 25 ($CDCl_3$, 500 MHz)

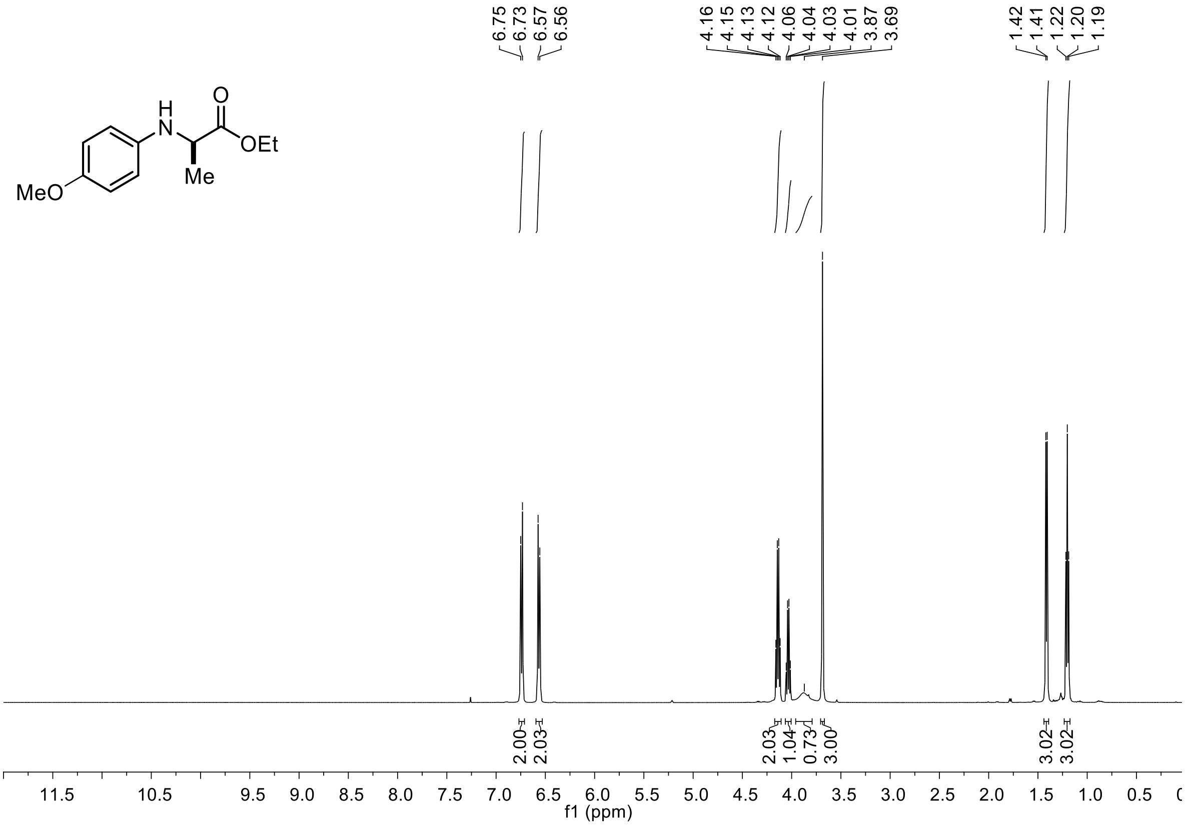

$^{13}C\{^{1}H\}$ NMR spectrum of 25 ($CDCl_3$, 126 MHz)

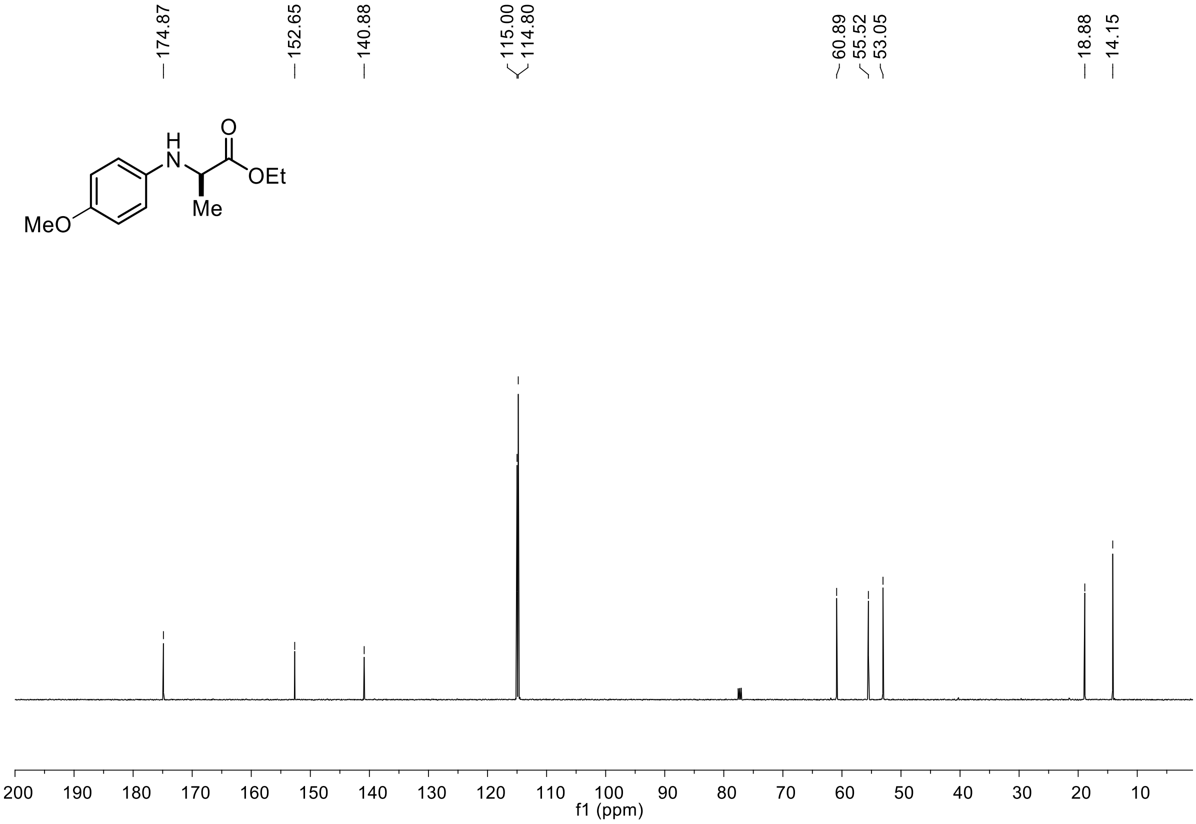

^{1}H NMR spectrum of 26 ($CDCl_3$, 500 MHz)

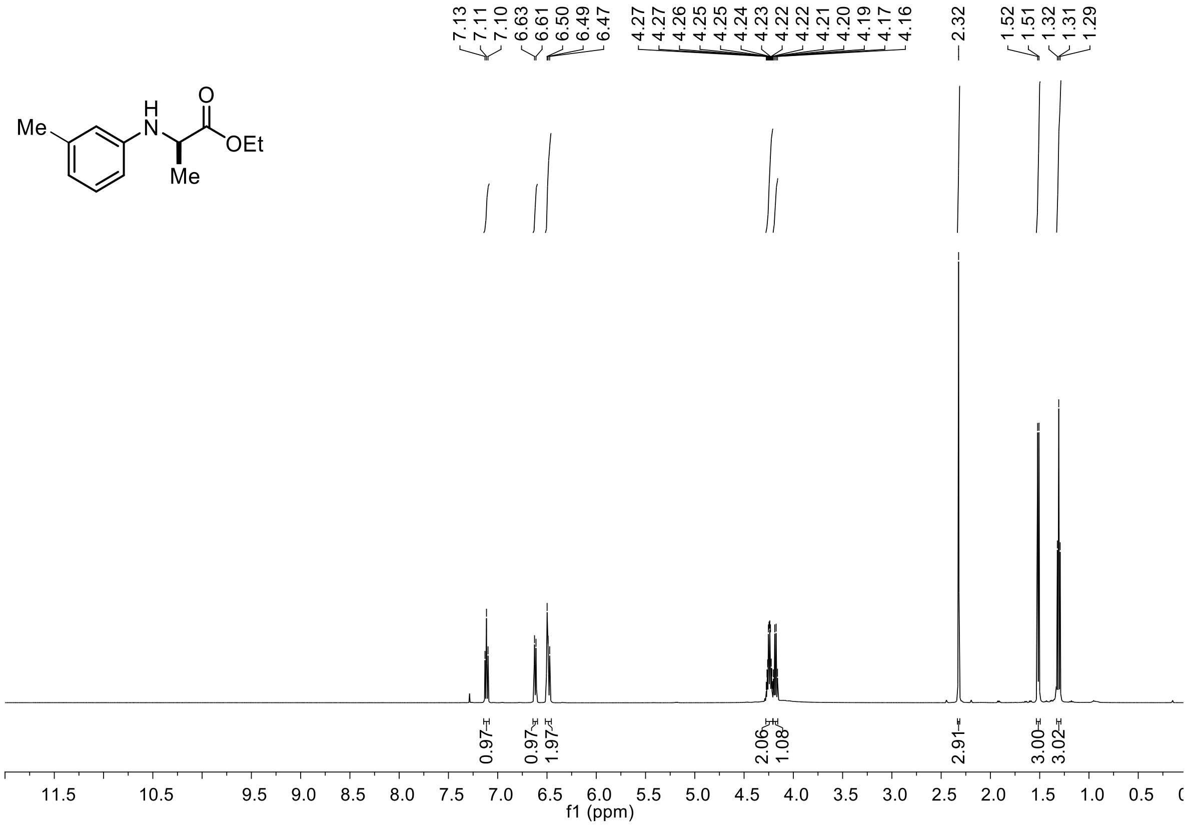

^{13}C{^{1}H} NMR spectrum of 26 ($CDCl_3$, 126 MHz)

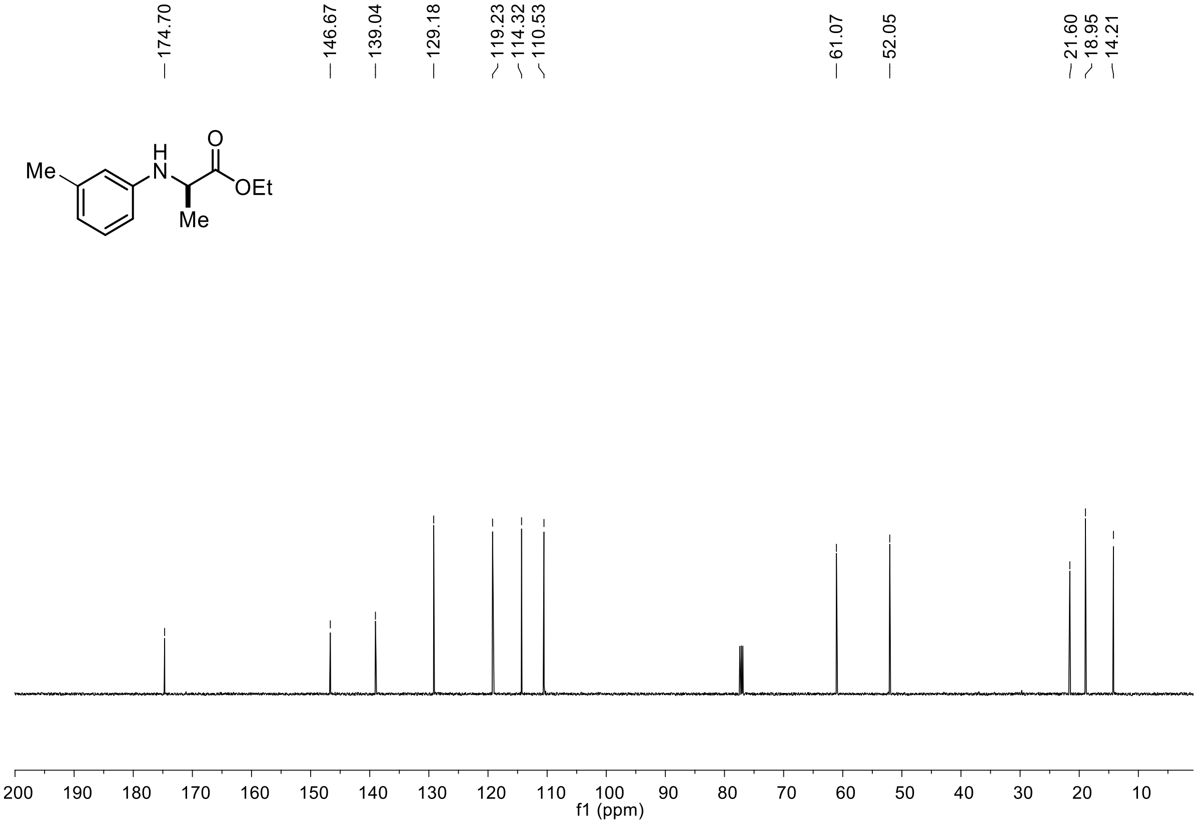

^{1}H NMR spectrum of 27 ($CDCl_3$, 500 MHz)

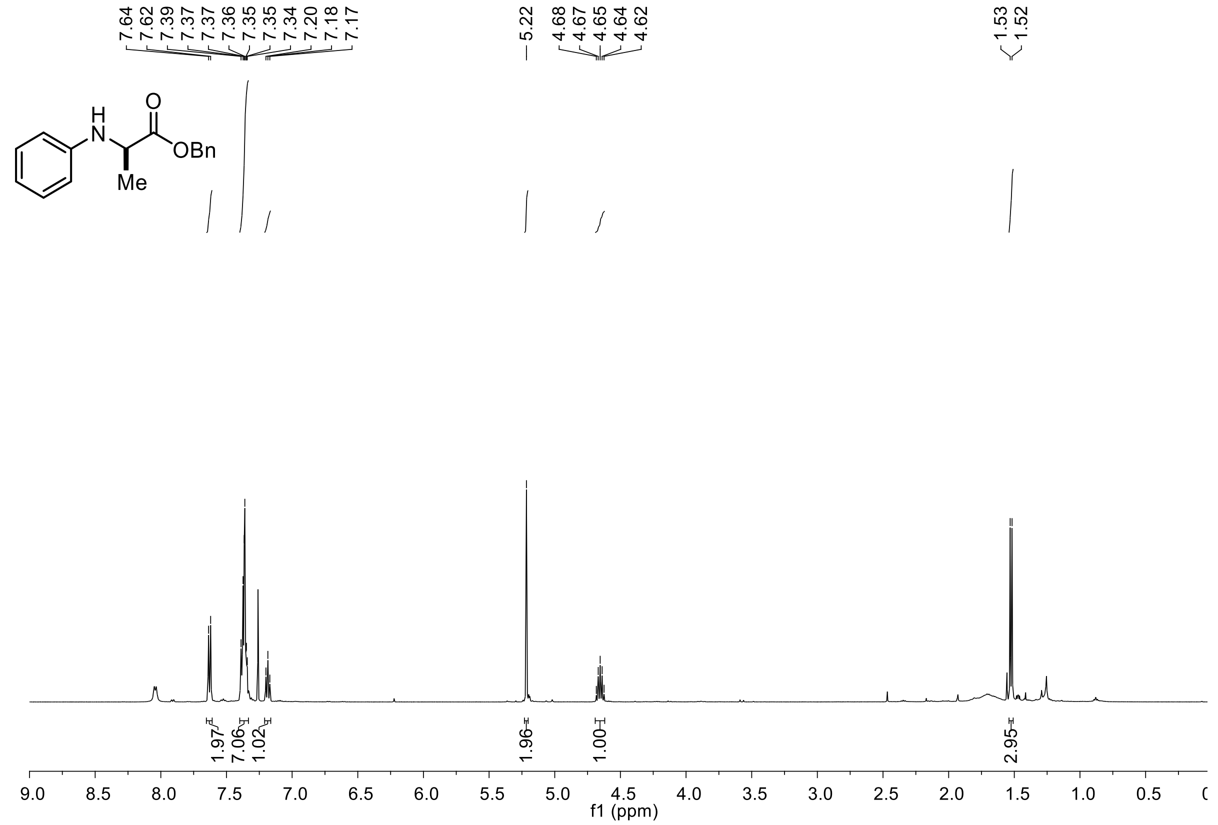

^{13}C{^{1}H} NMR spectrum of 27 ($CDCl_3$, 126 MHz)

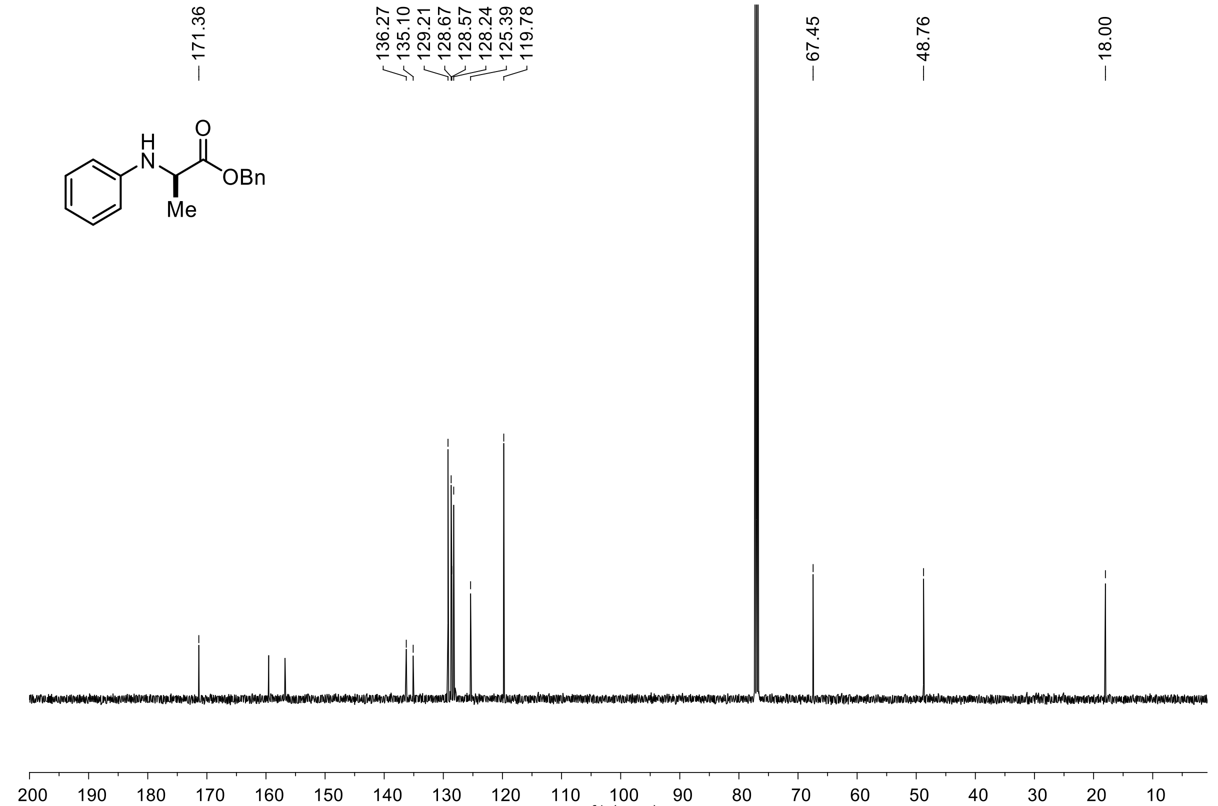

^{1}H NMR spectrum of 28 (CDCl$_3$, 500 MHz)

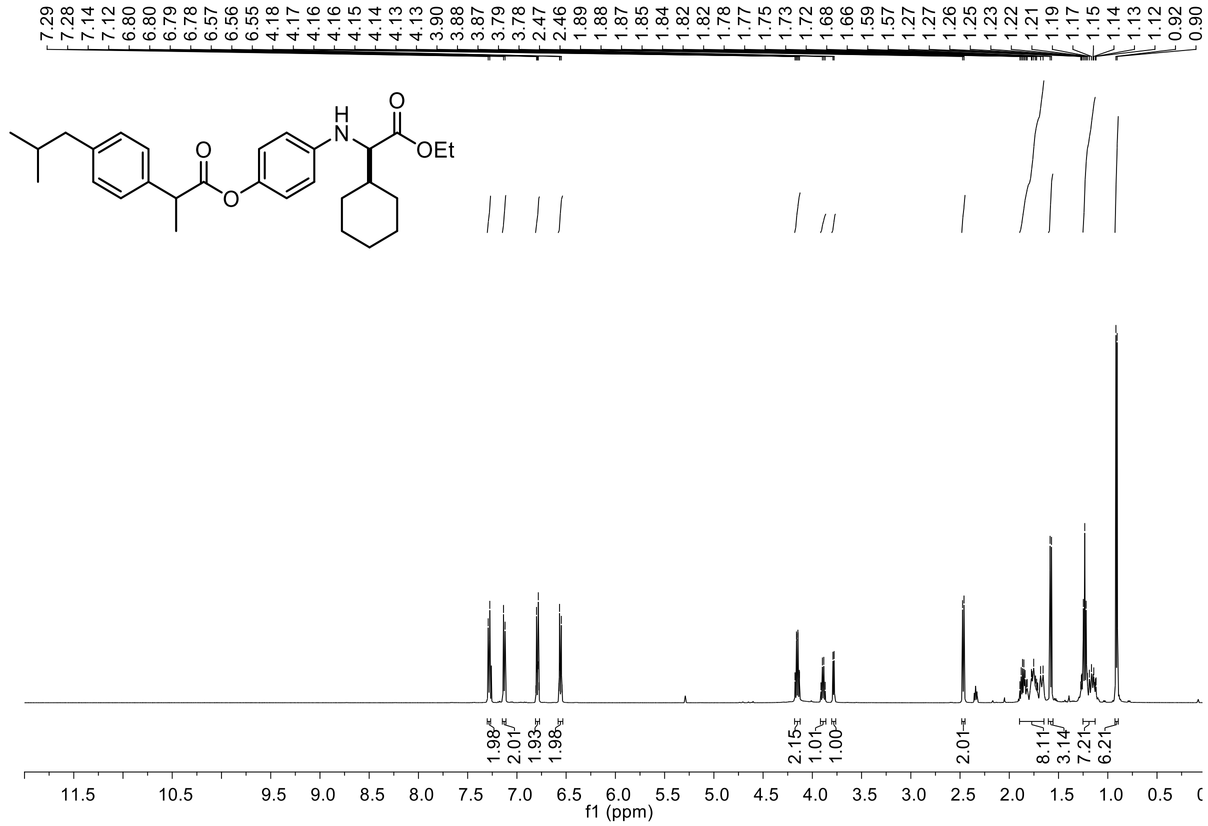

^{13}C{^{1}H} NMR spectrum of 28 (CDCl$_3$, 126 MHz)

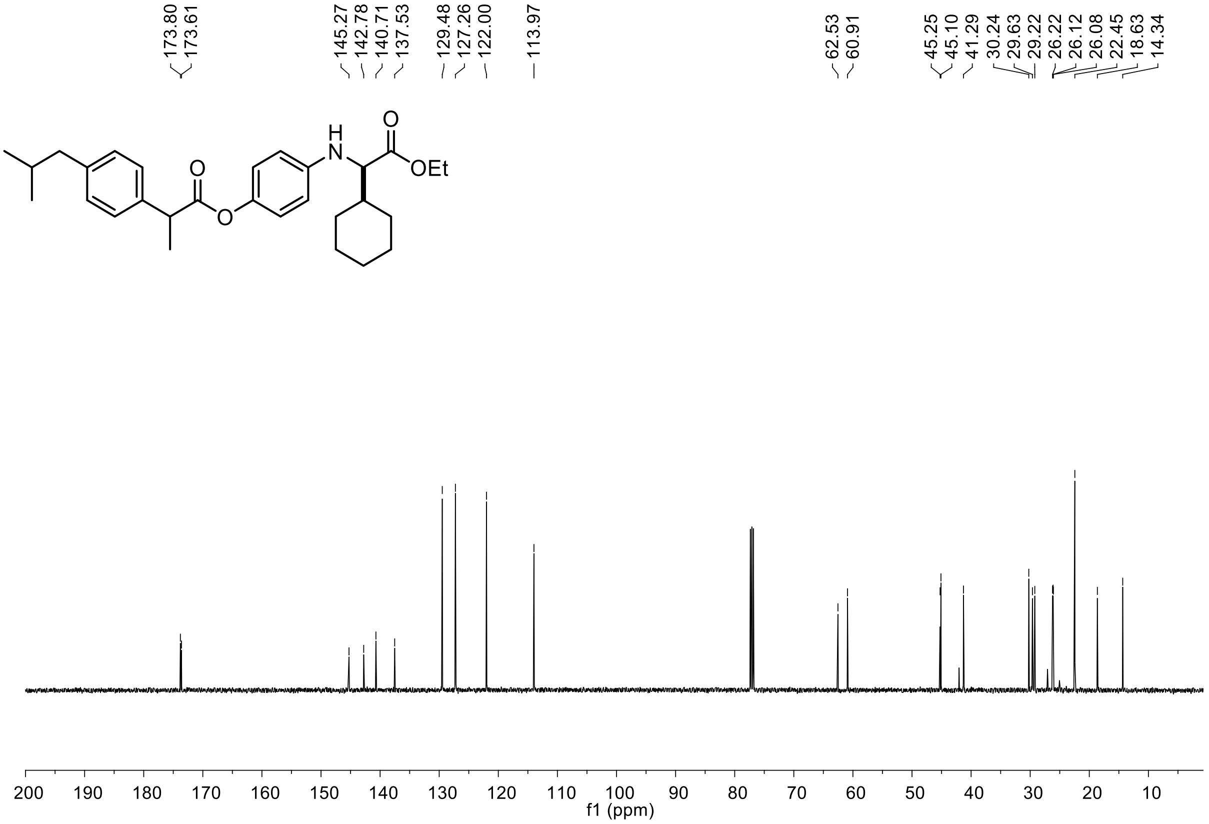

^{1}H NMR spectrum of 29 ($CDCl_3$, 500 MHz)

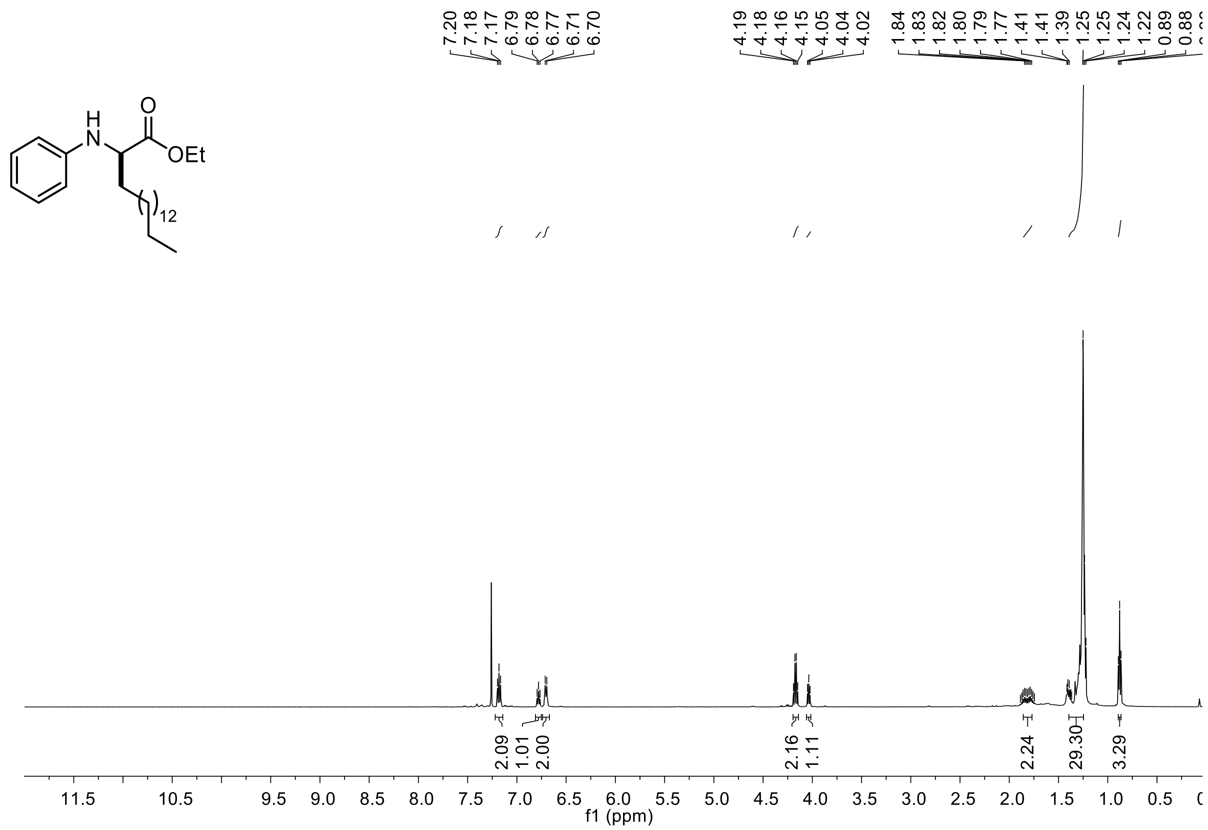

^{13}C{^{1}H} NMR spectrum of 29 ($CDCl_3$, 126 MHz)

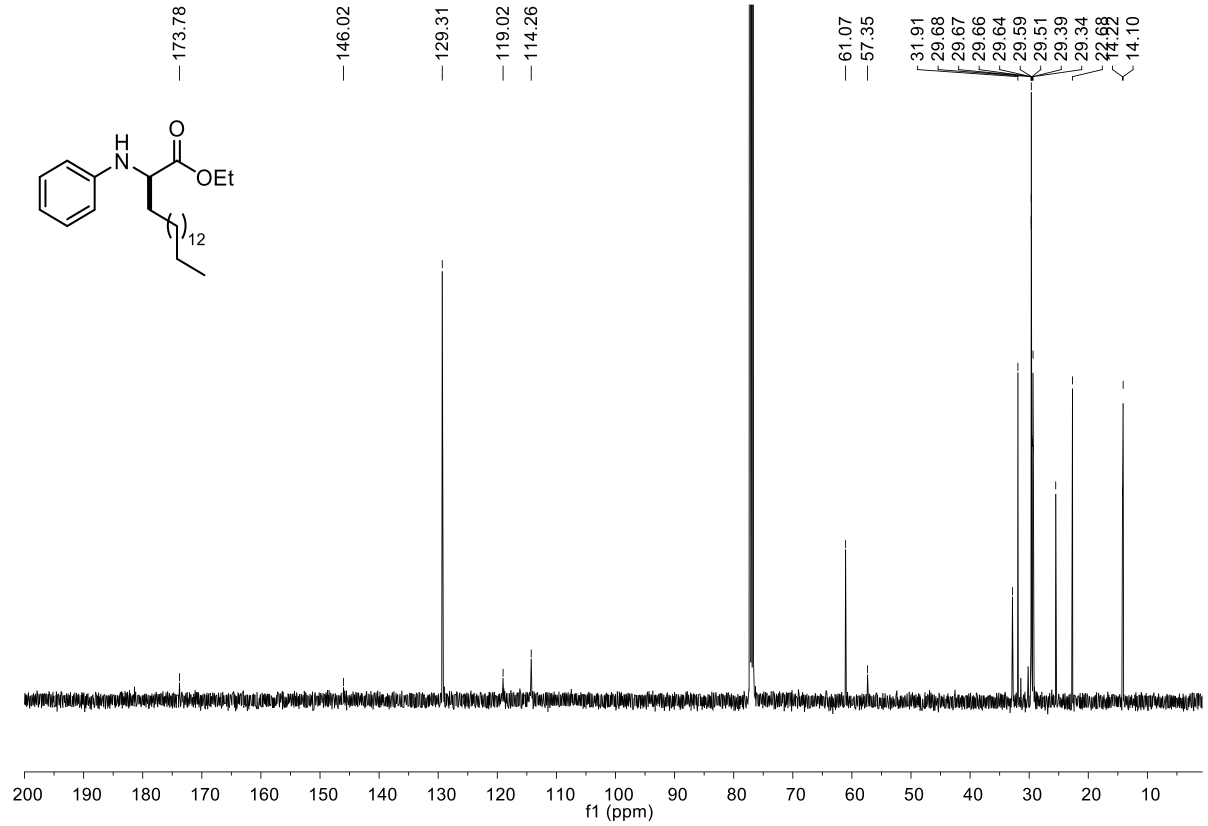

^{1}H NMR spectrum of 30 ($CDCl_3$, 500 MHz)

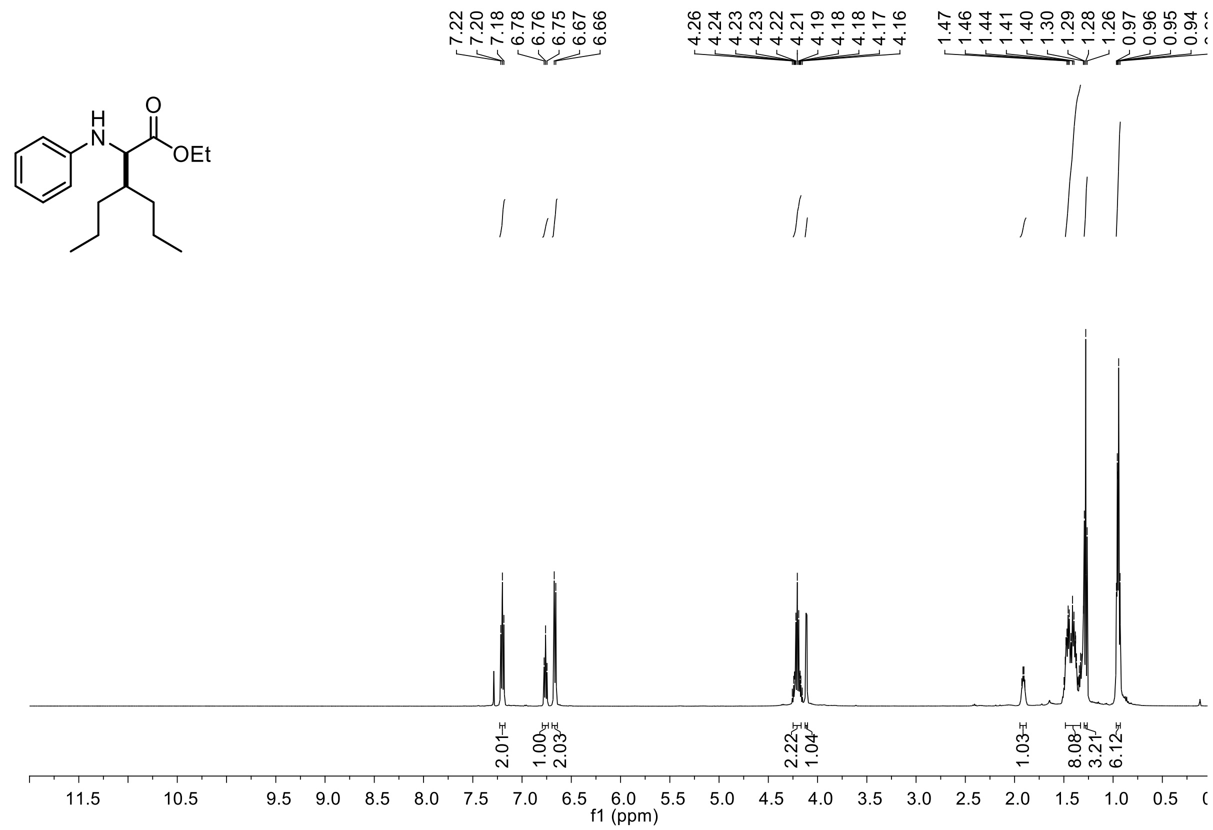

$^{13}C\{^{1}H\}$ NMR spectrum of 30 ($CDCl_3$, 126 MHz)

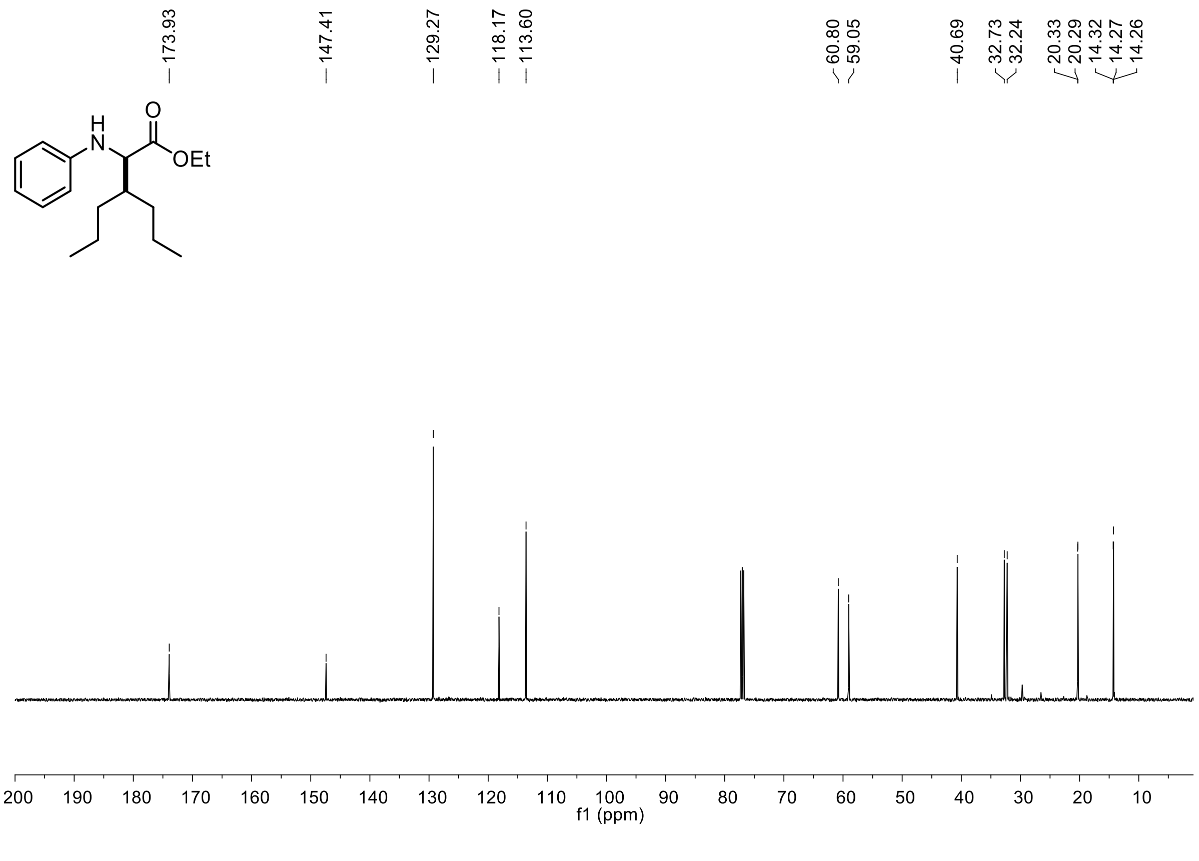

^{1}H NMR spectrum of 31 ($CDCl_3$, 500 MHz)

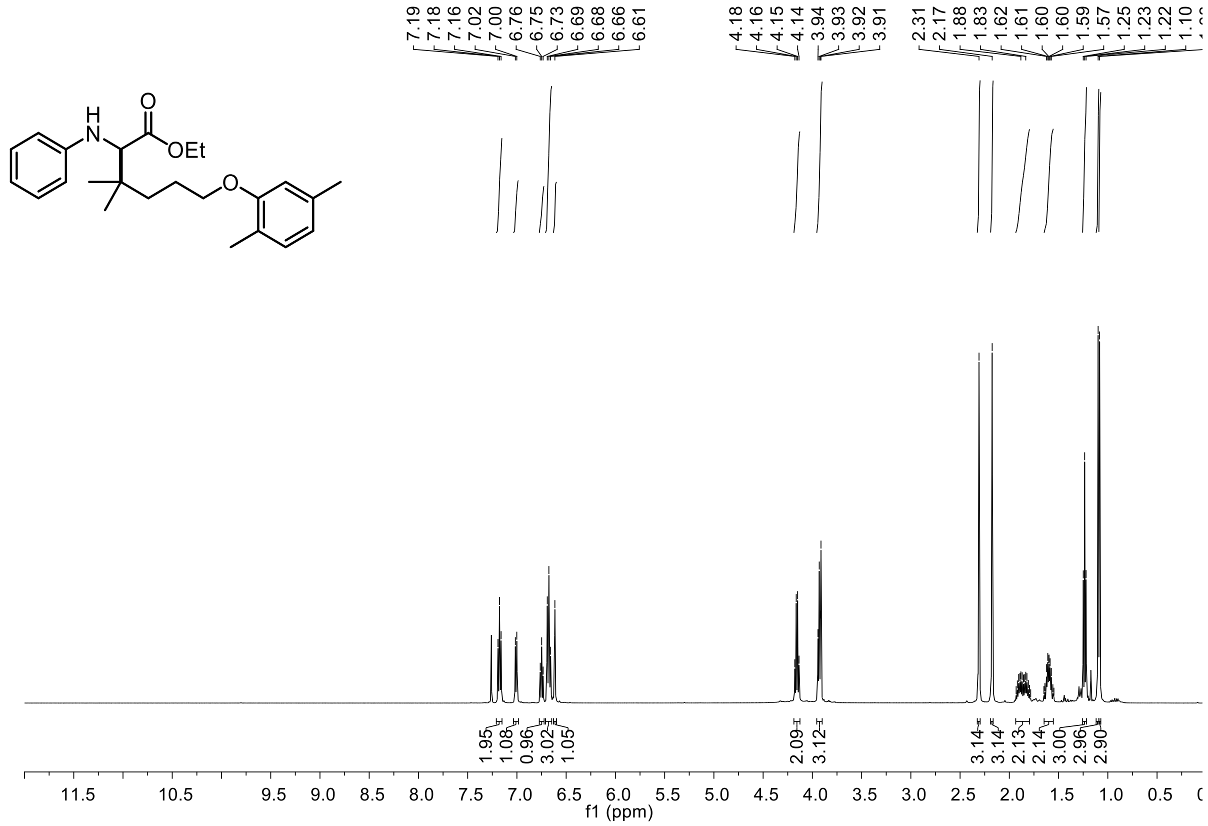

^{13}C{^{1}H} NMR spectrum of 31 ($CDCl_3$, 126 MHz)

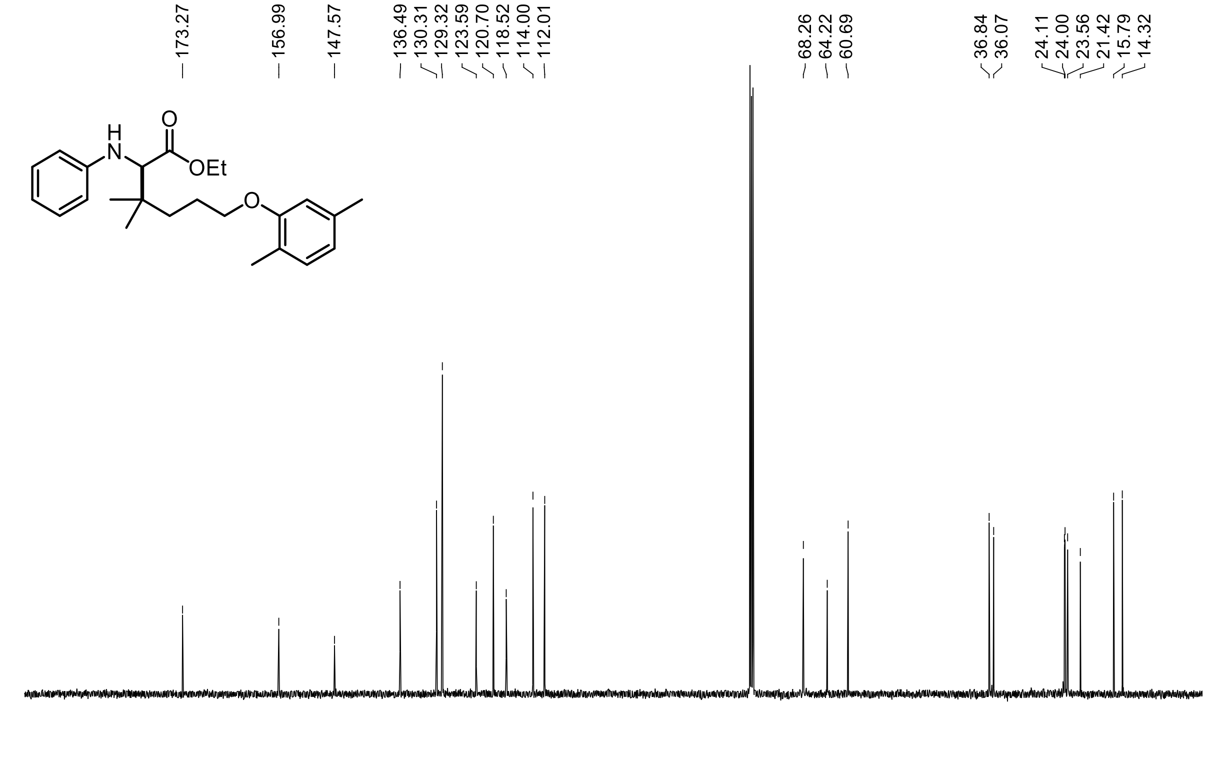

^{1}H NMR spectrum of 32 ($CDCl_3$, 500 MHz)

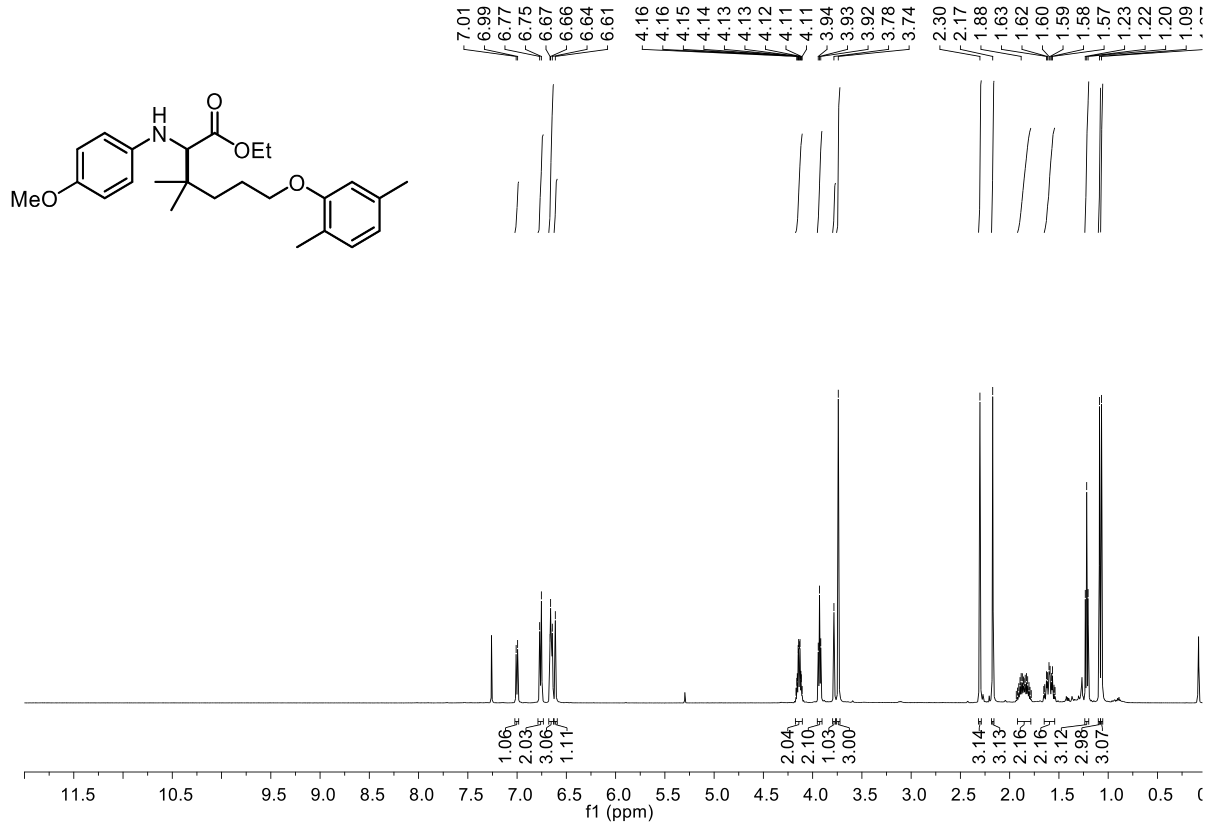

^{13}C{^{1}H} NMR spectrum of 32 ($CDCl_3$, 126 MHz)

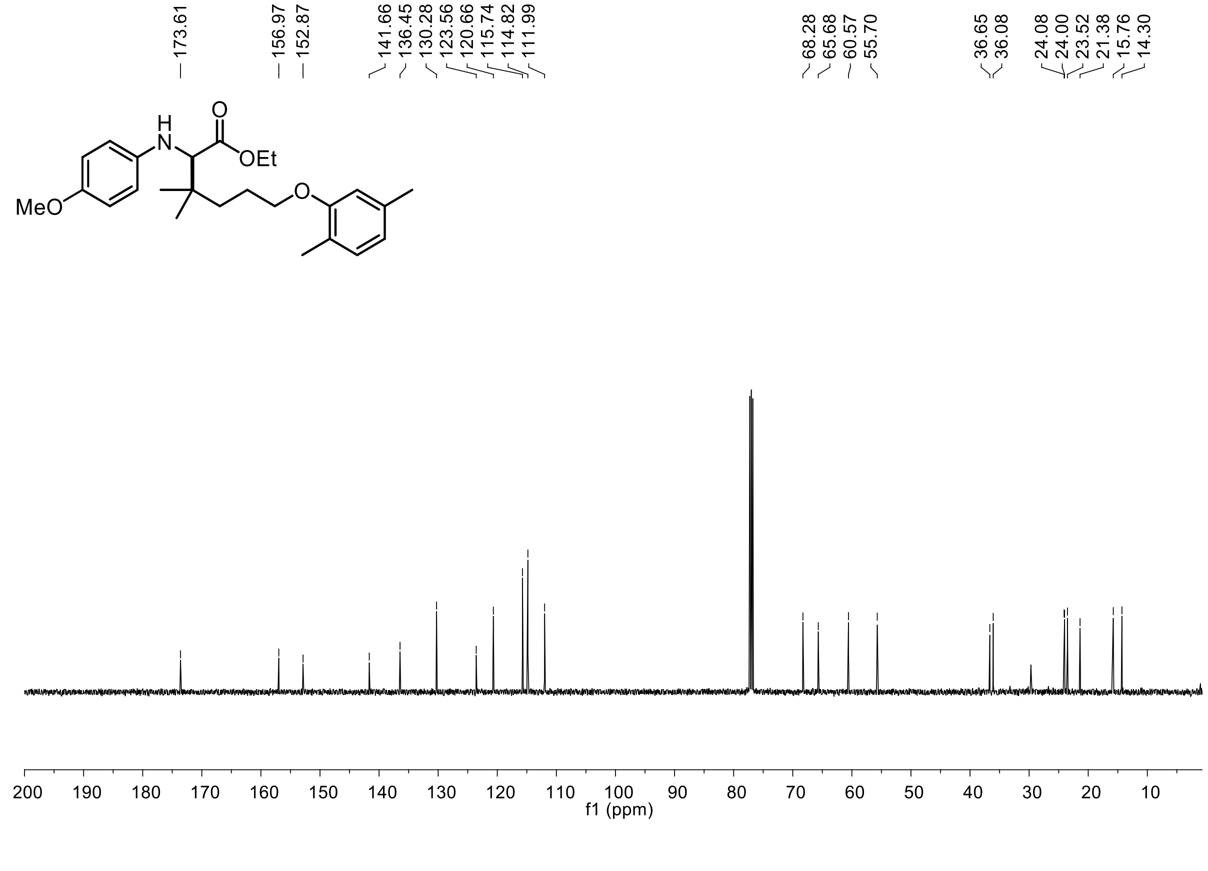

^{1}H NMR spectrum of 33 ($CDCl_3$, 500 MHz)

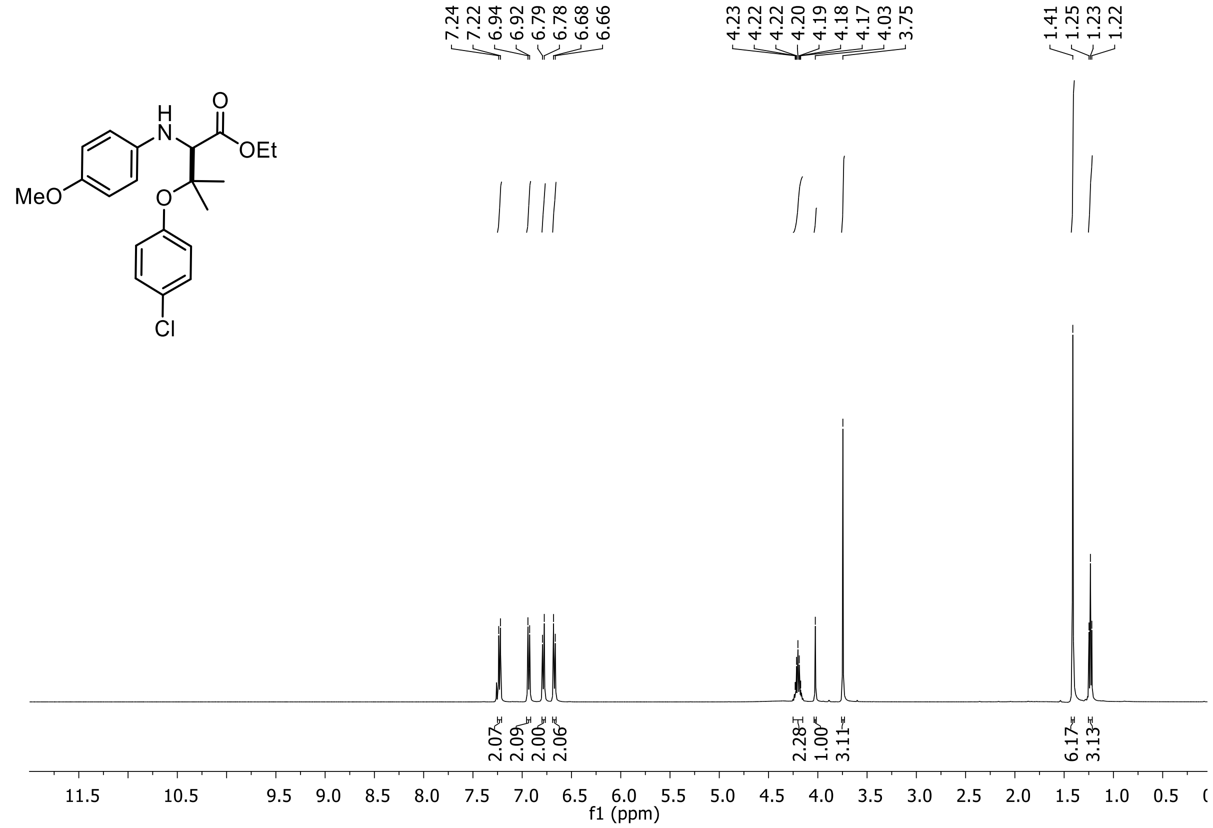

$^{13}C\{^{1}H\}$ NMR spectrum of 33 ($CDCl_3$, 126 MHz)

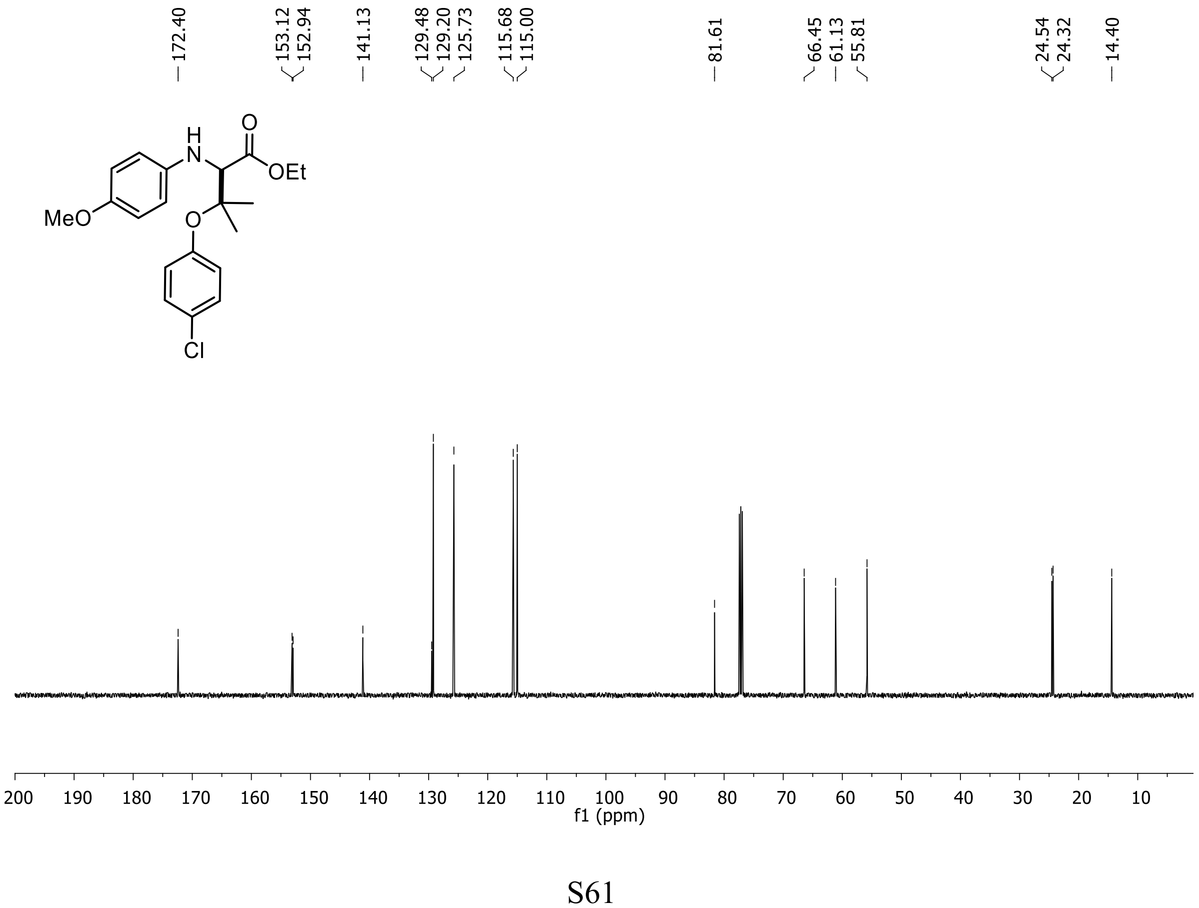

^{1}H NMR spectrum of 34 ($CDCl_3$, 500 MHz)

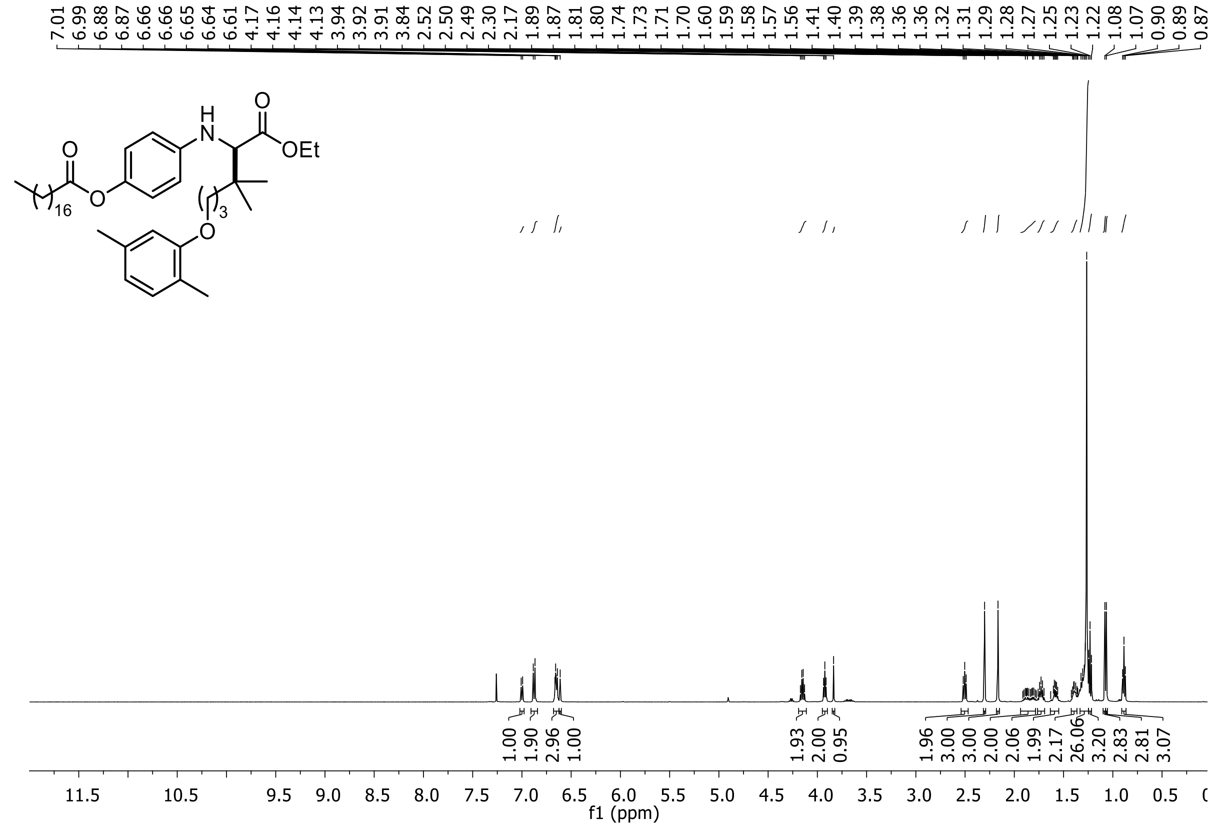

^{13}C{^{1}H} NMR spectrum of 34 ($CDCl_3$, 126 MHz)

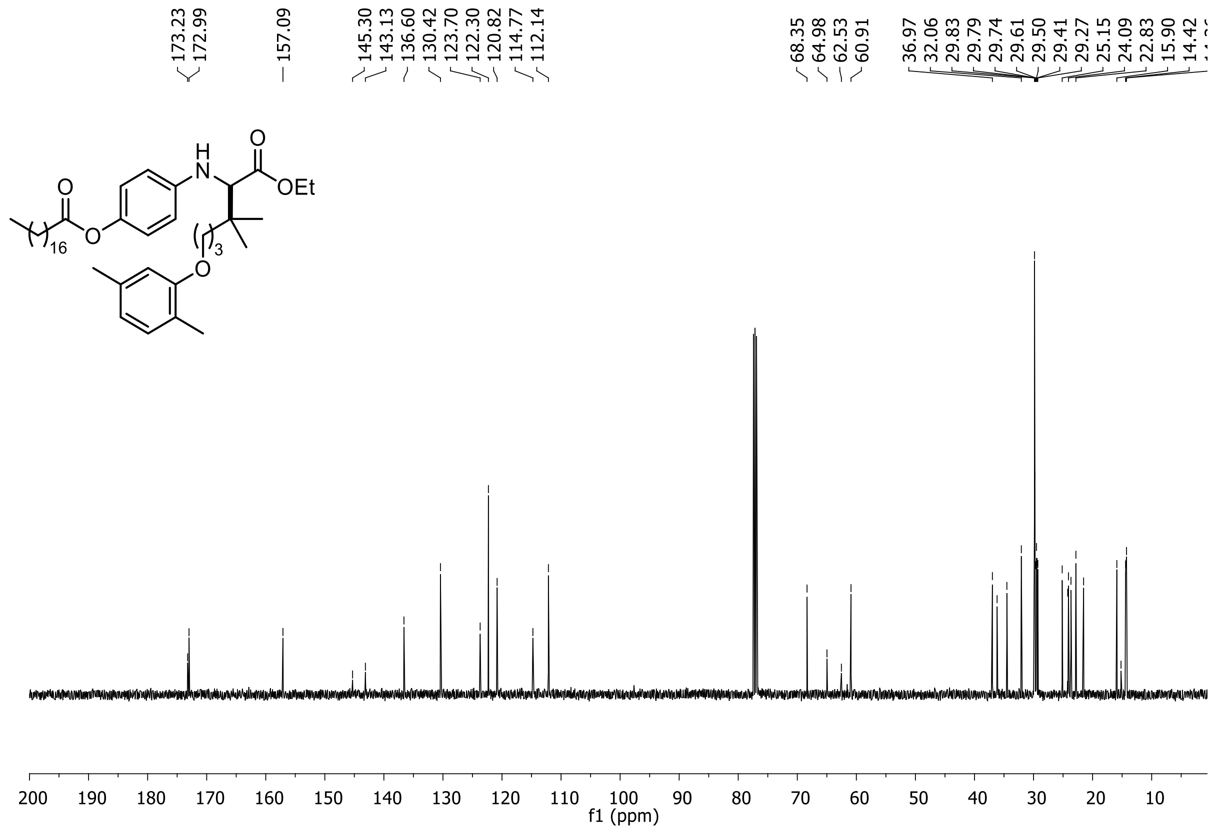

^{1}H NMR spectrum of 35 ($CDCl_3$, 500 MHz)

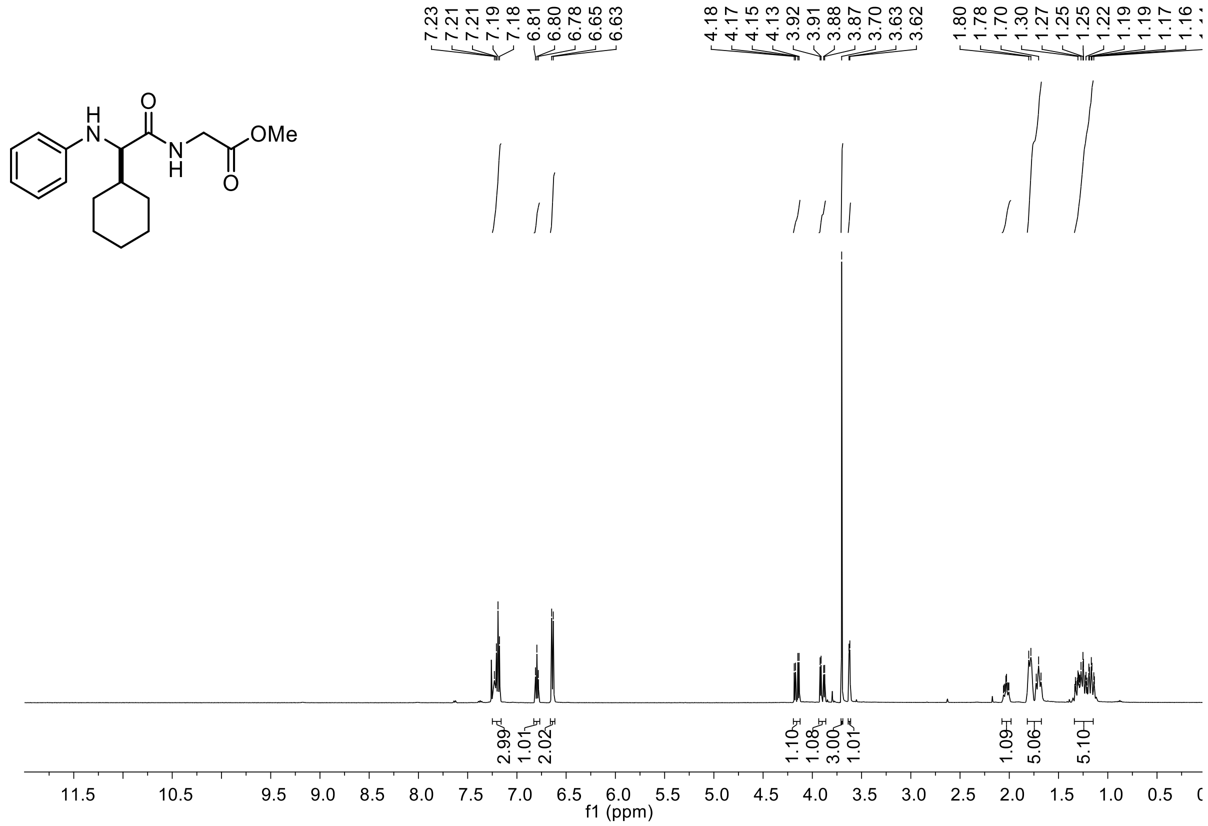

^{13}C{^{1}H} NMR spectrum of 35 ($CDCl_3$, 126 MHz)

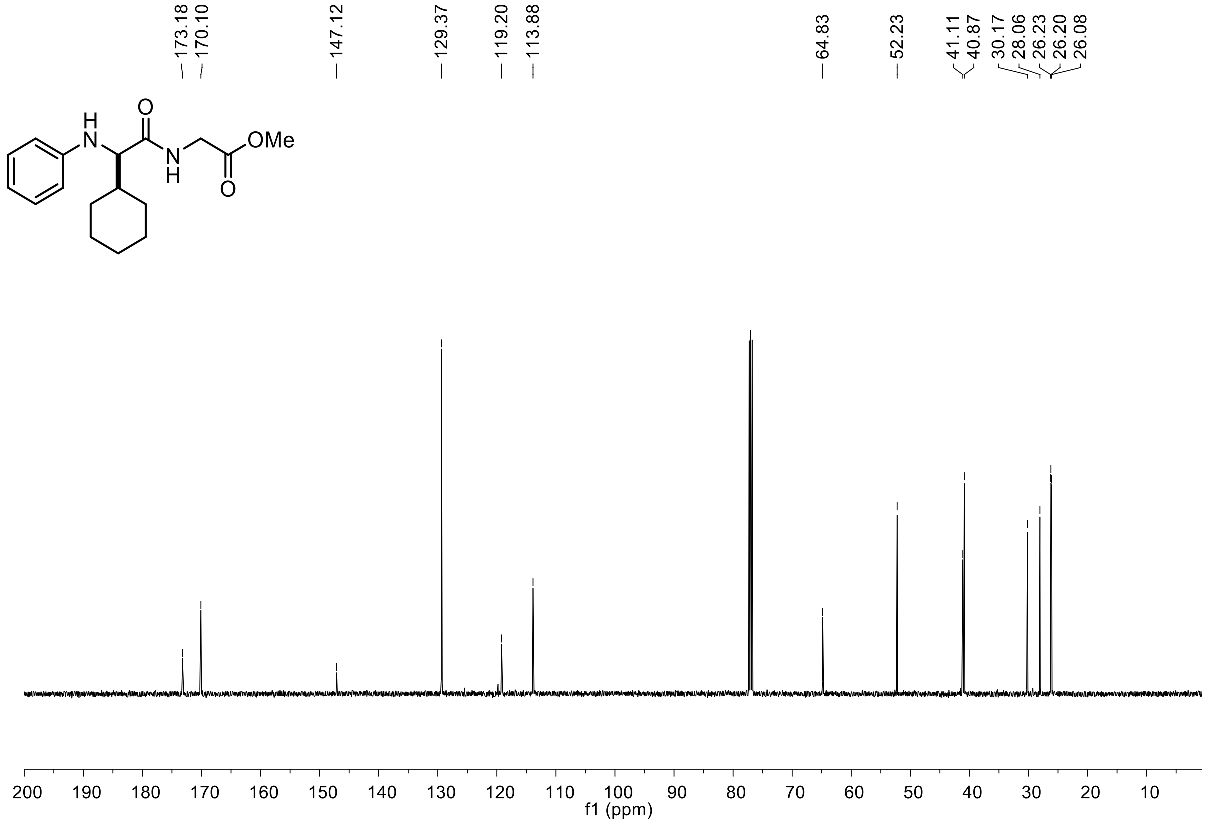

^{1}H NMR spectrum of 36 ($CDCl_3$, 500 MHz)

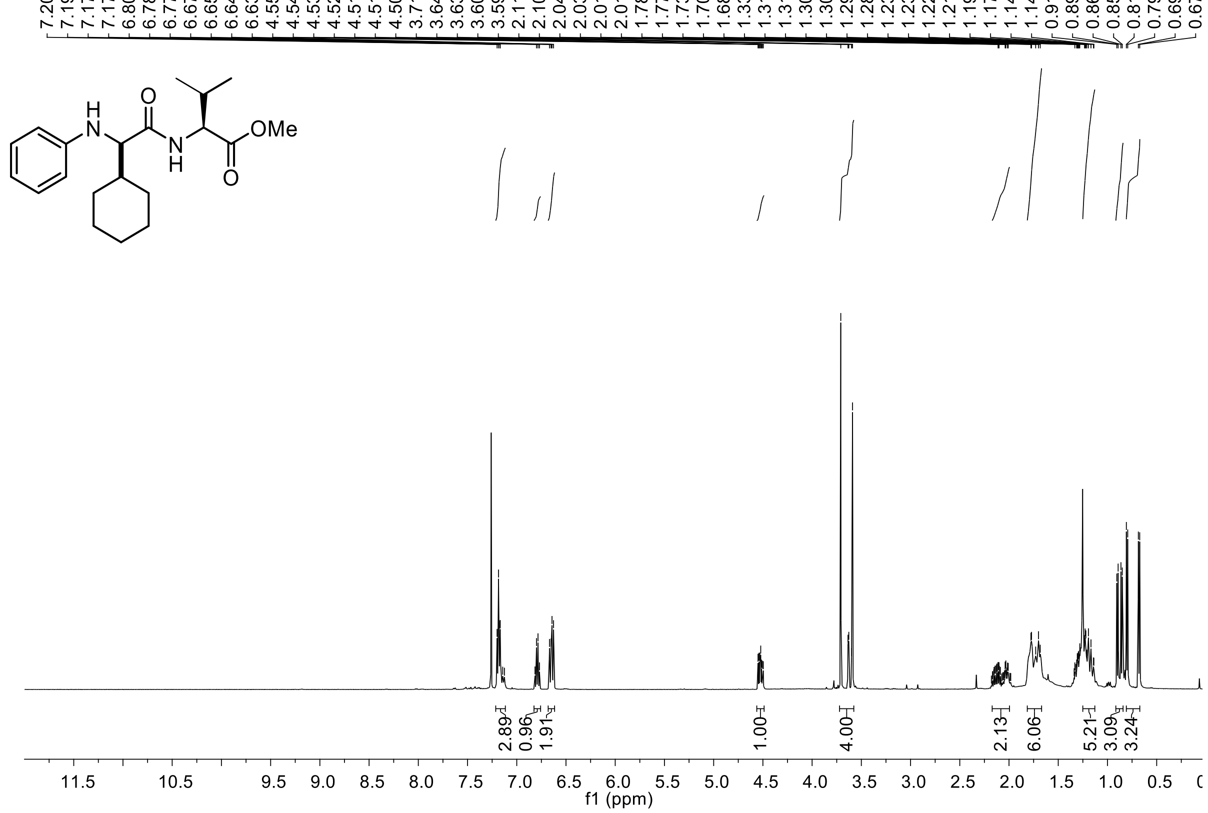

^{13}C{^{1}H} NMR spectrum of 36 ($CDCl_3$, 126 MHz)

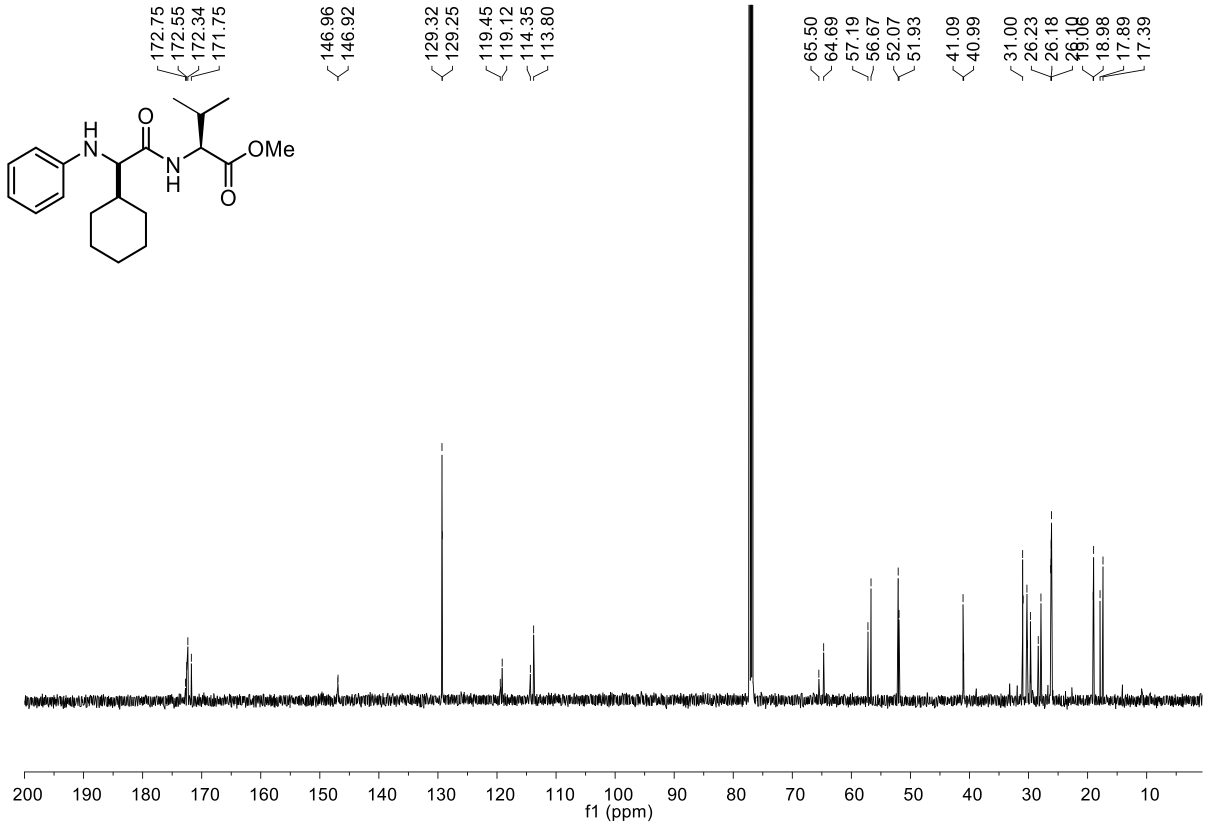

^{1}H NMR spectrum of 37 ($CDCl_3$, 500 MHz)

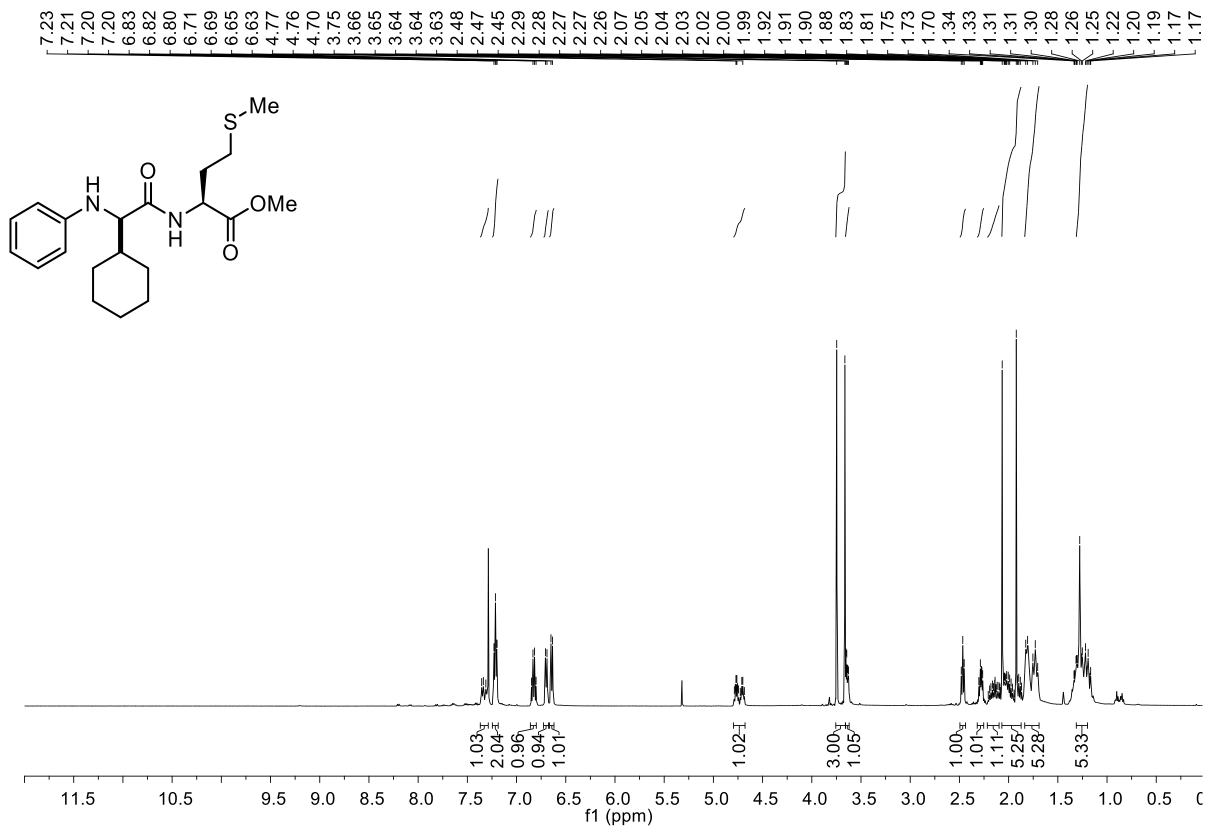

^{13}C{^{1}H} NMR spectrum of 37 ($CDCl_3$, 126 MHz)

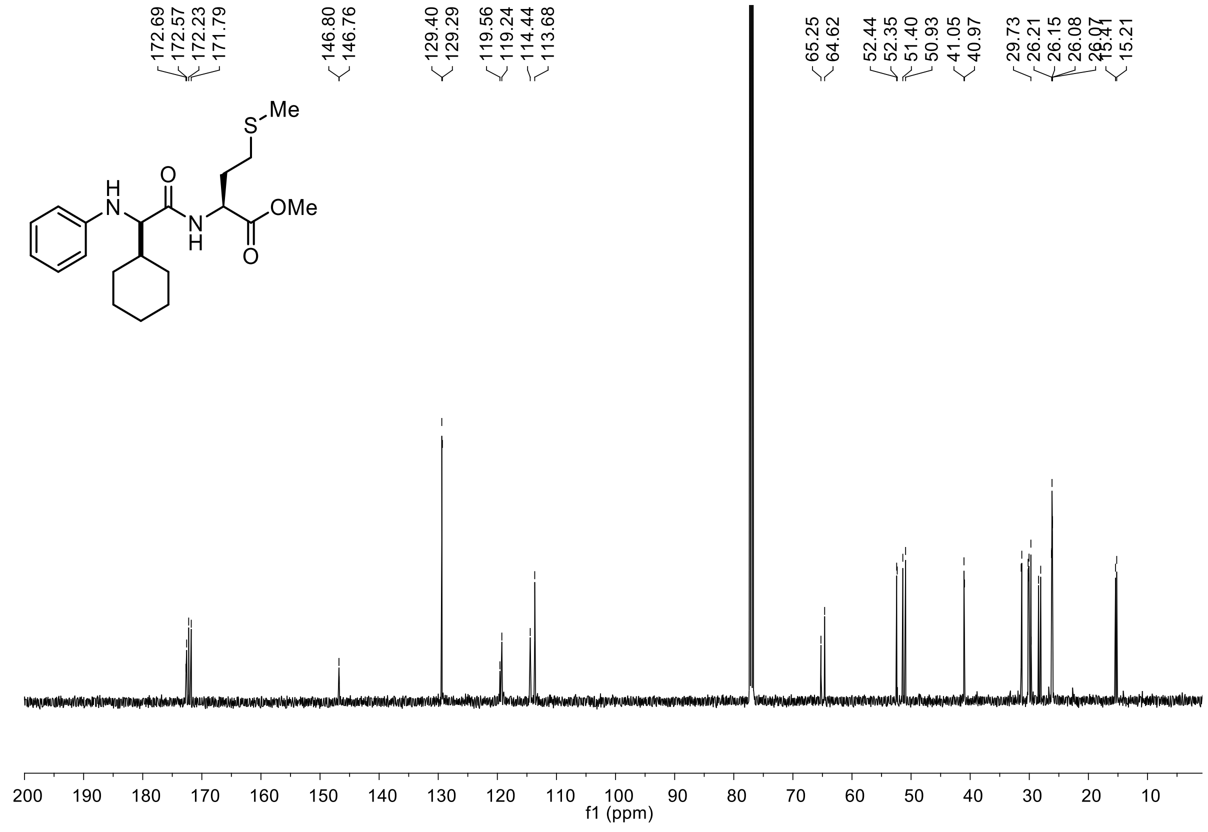

^{1}H NMR spectrum of 38 ($CDCl_3$, 500 MHz)

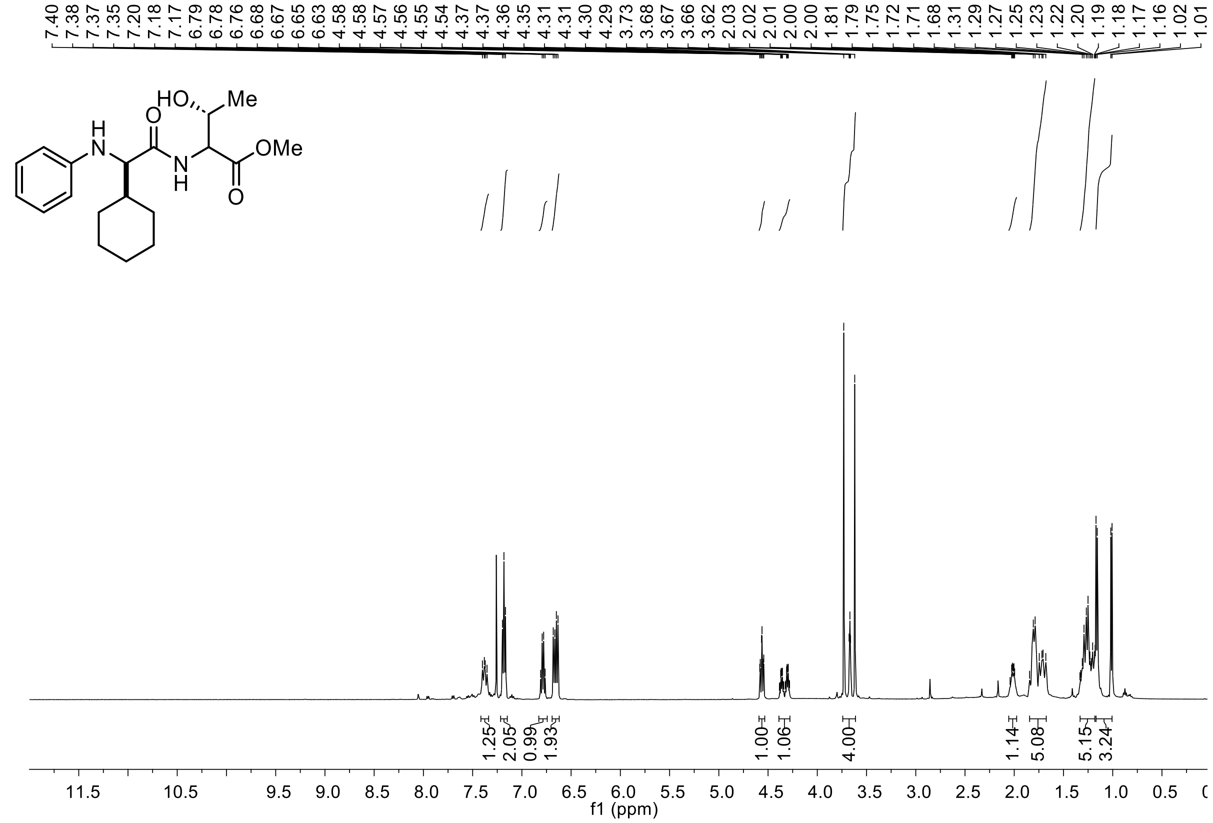

^{13}C{^{1}H} NMR spectrum of 38 ($CDCl_3$, 126 MHz)

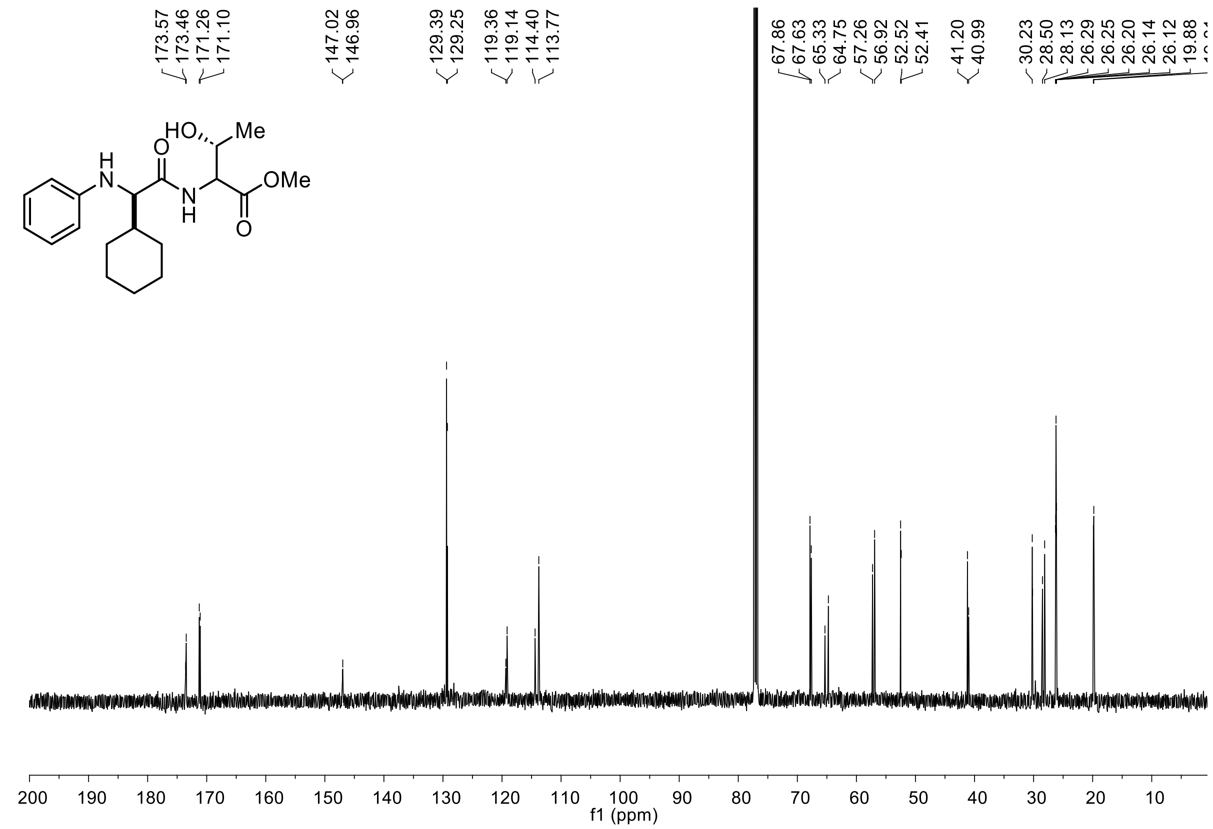

^{1}H NMR spectrum of 39 ($CDCl_3$, 500 MHz)

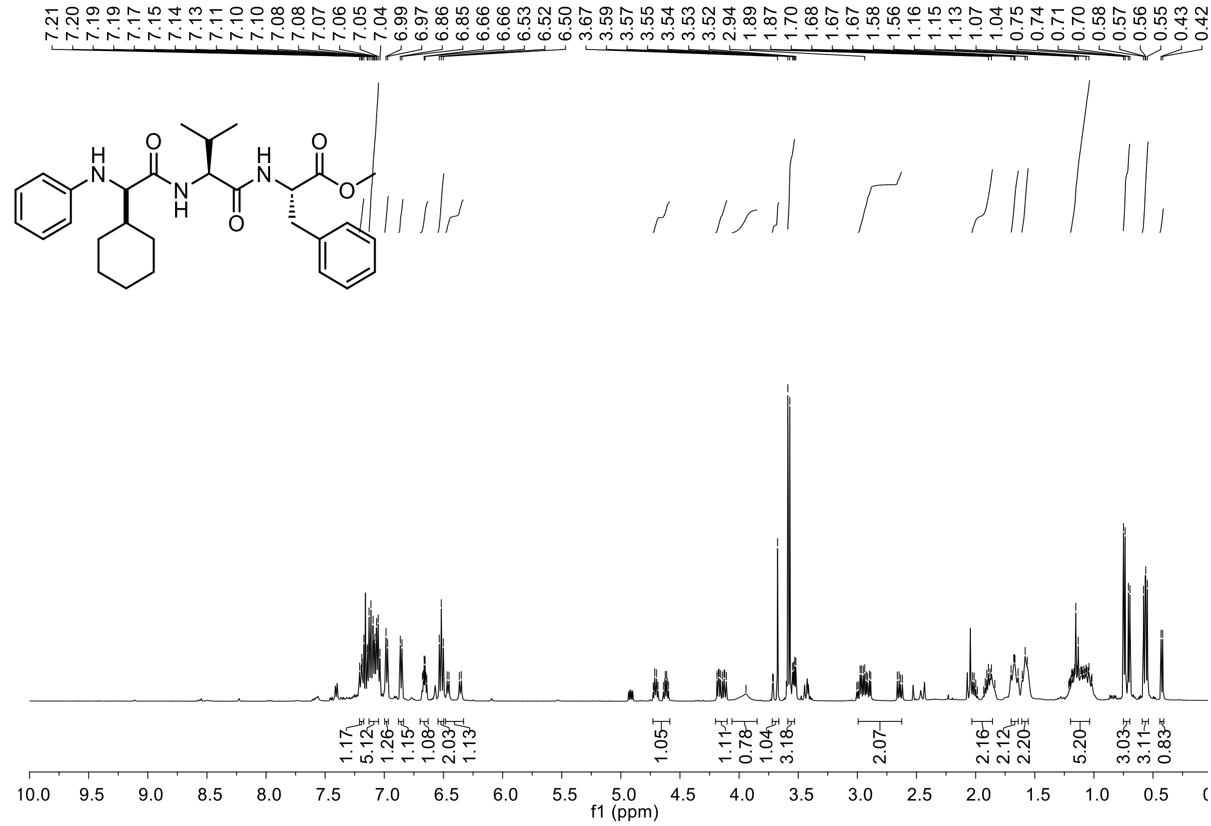

$^{13}C\{^1H\}$ NMR spectrum of 39 ($CDCl_3$, 126 MHz)

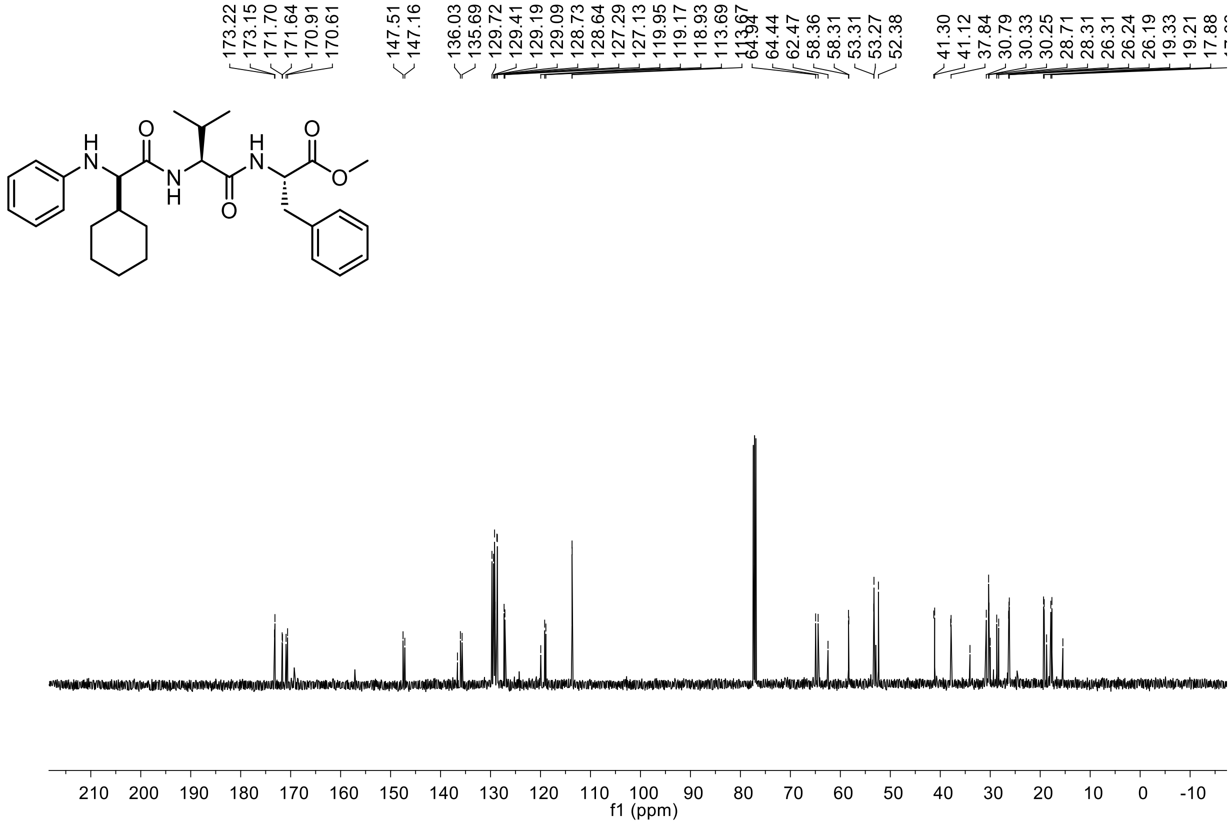

^{1}H NMR spectrum of 40 ($CDCl_3$, 500 MHz)

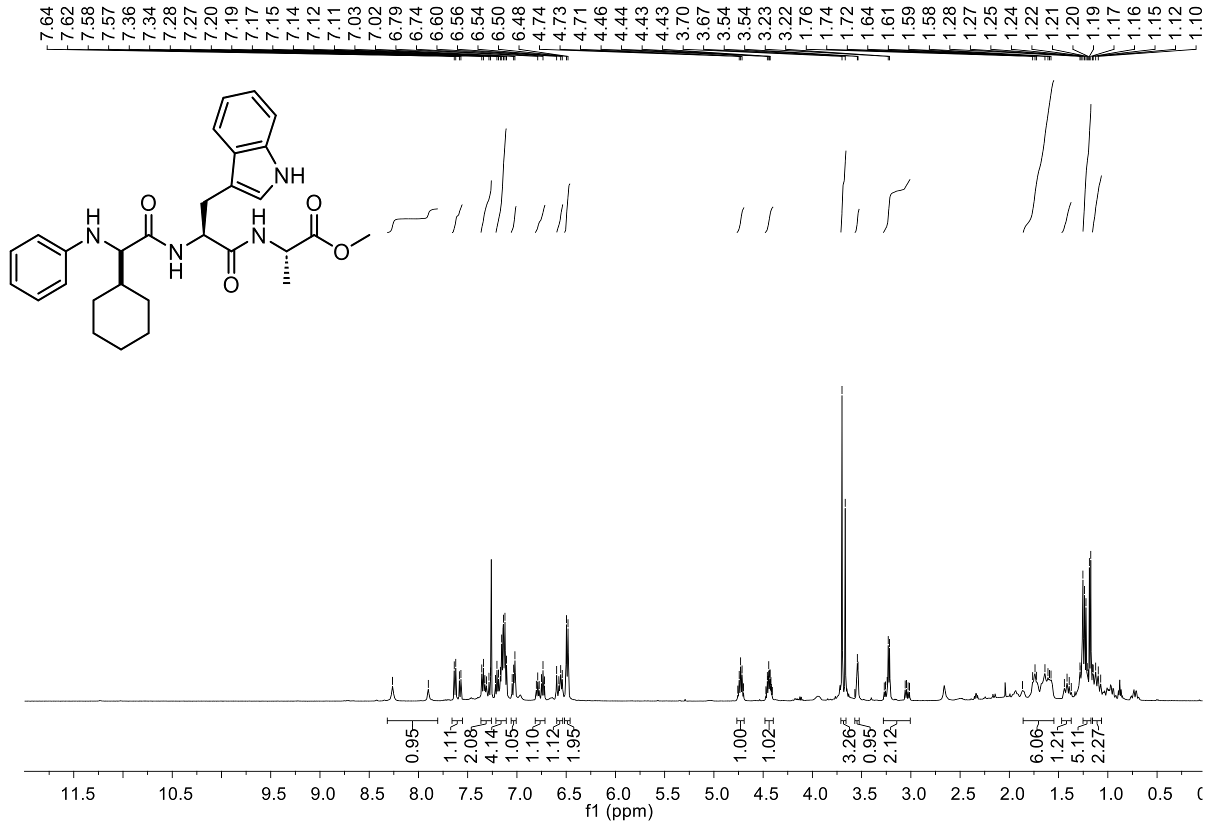

^{13}C{^{1}H} NMR spectrum of 40 ($CDCl_3$, 126 MHz)

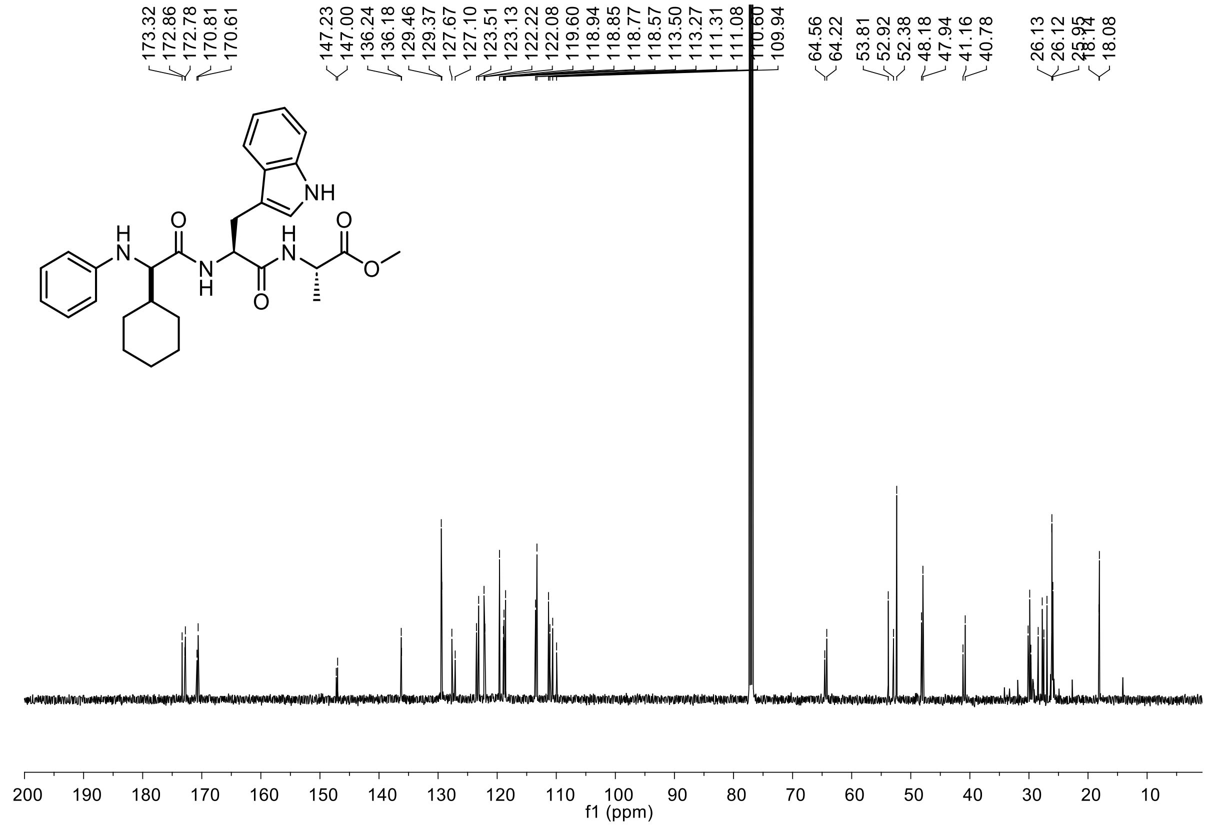

^{1}H NMR spectrum of 41 (CDCl$_3$, 500 MHz)

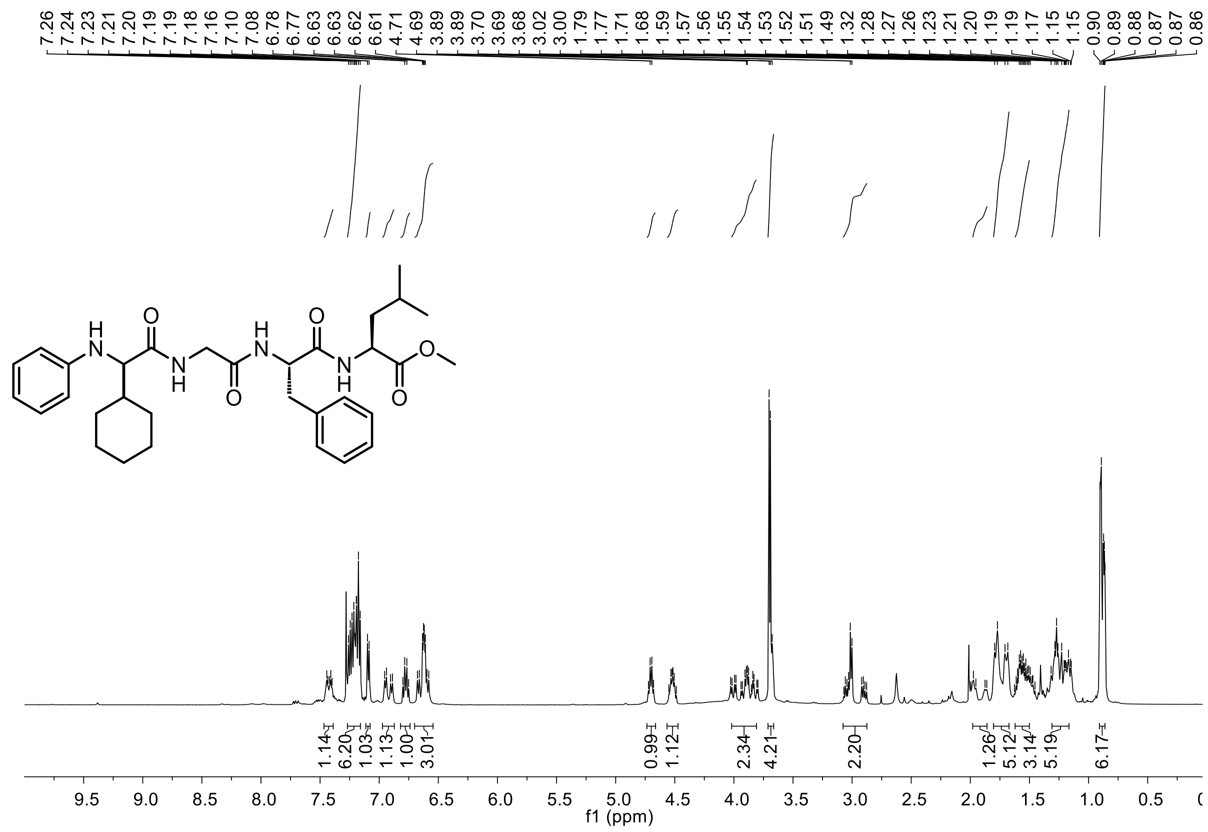

^{13}C{^{1}H} NMR spectrum of 41 (CDCl$_3$, 126 MHz)

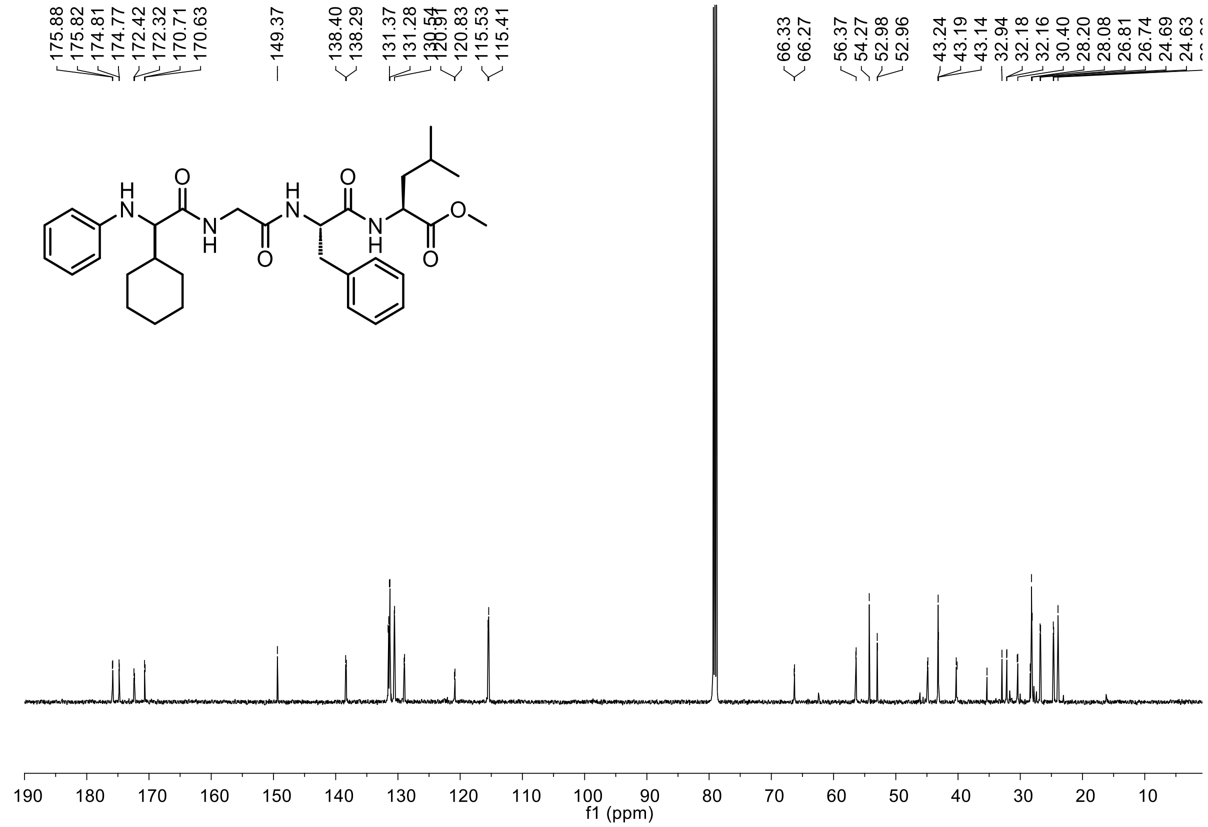

^{1}H NMR spectrum of 42 ($CDCl_3$, 500 MHz)

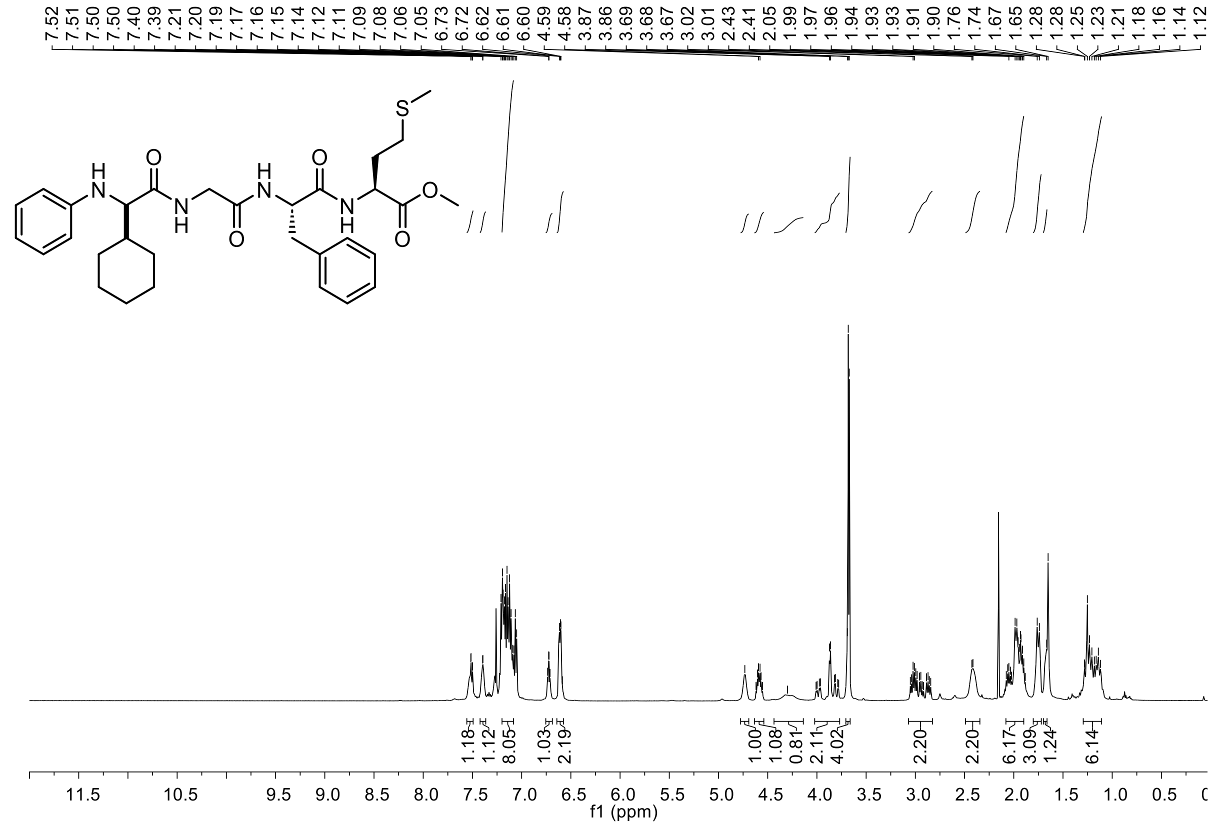

$^{13}C\{^{1}H\}$ NMR spectrum of 42 ($CDCl_3$, 126 MHz)

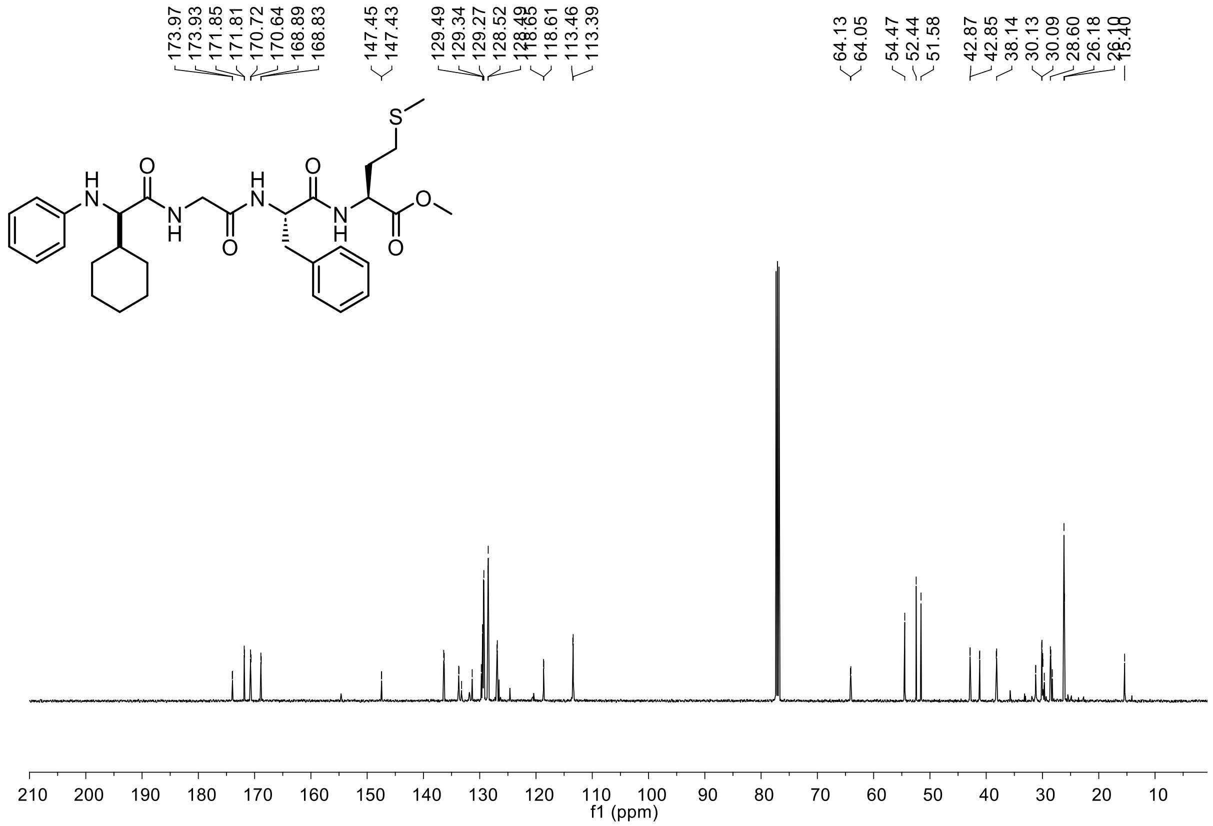

^{1}H NMR spectrum of 43 (DMSO, 500 MHz)

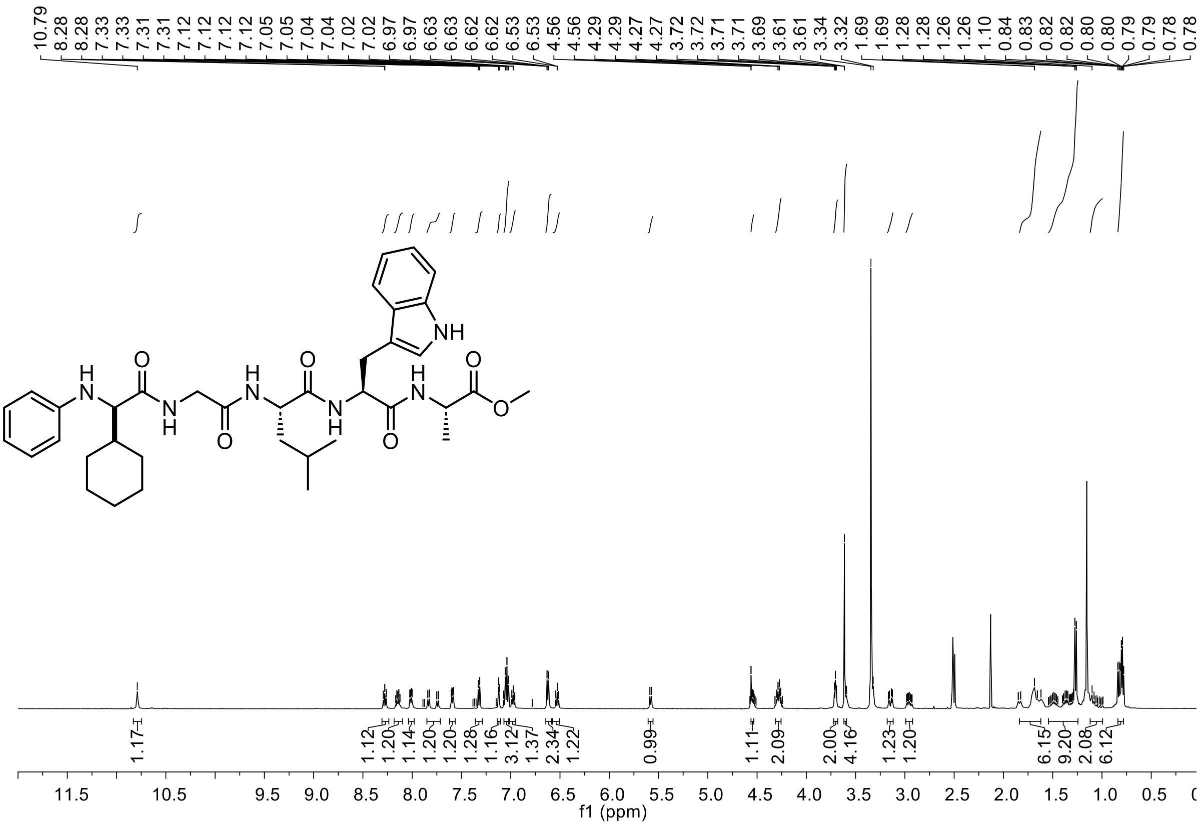

^{13}C{^{1}H} NMR spectrum of 43 (DMSO, 126 MHz)

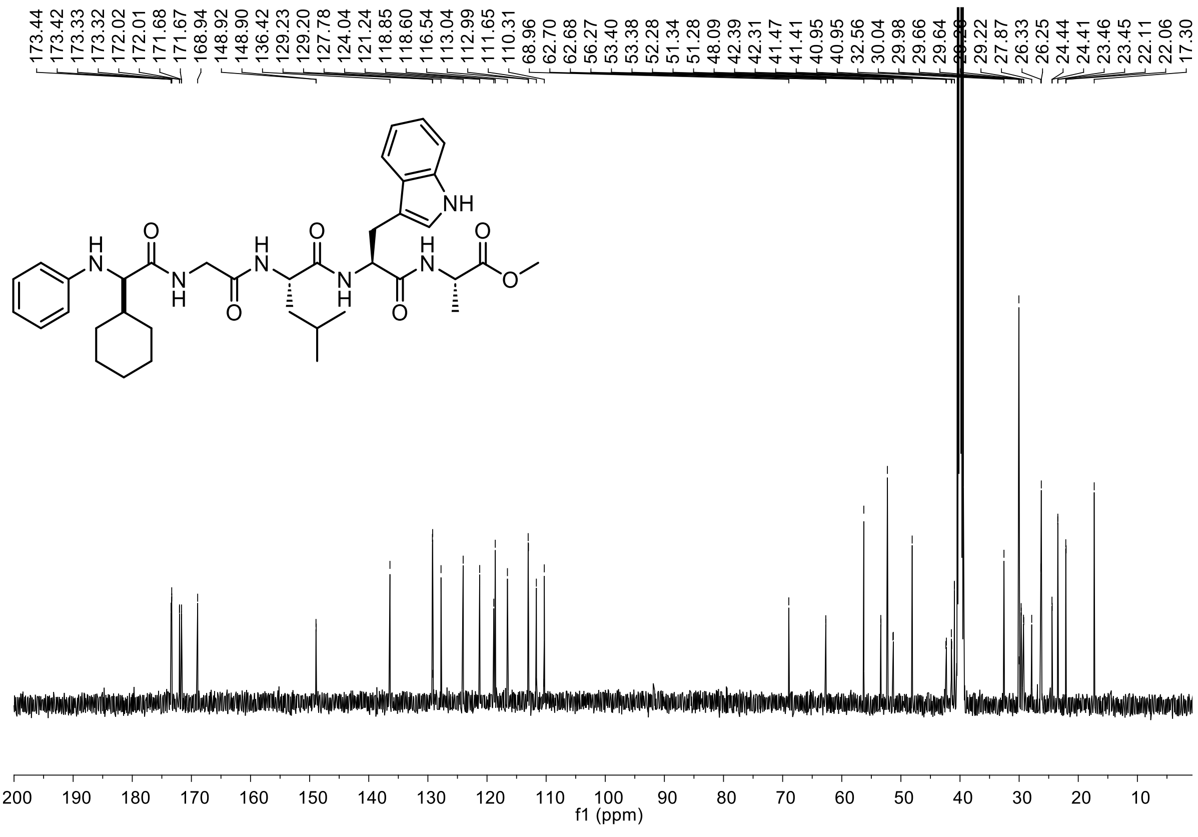

^{1}H NMR spectrum of 44 ($CDCl_3$, 500 MHz)

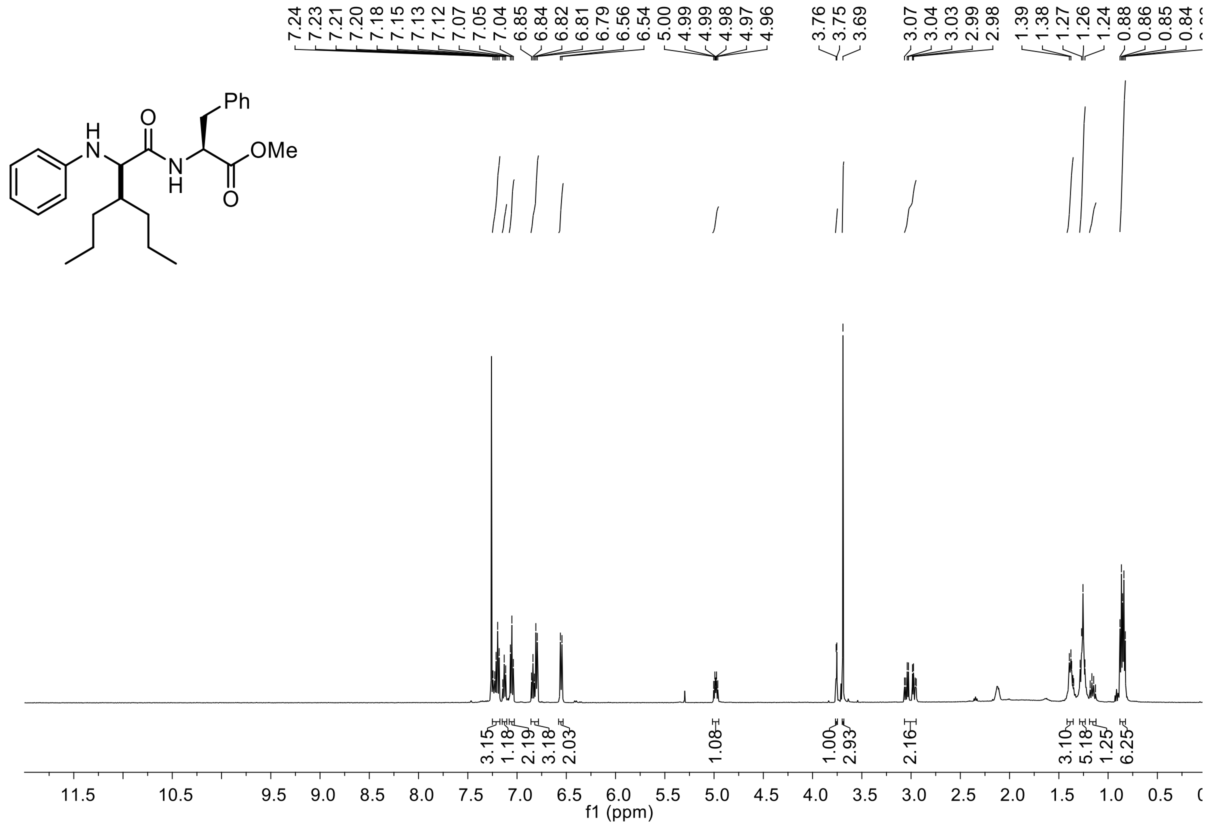

$^{13}C\{^{1}H\}$ NMR spectrum of 44 ($CDCl_3$, 126 MHz)

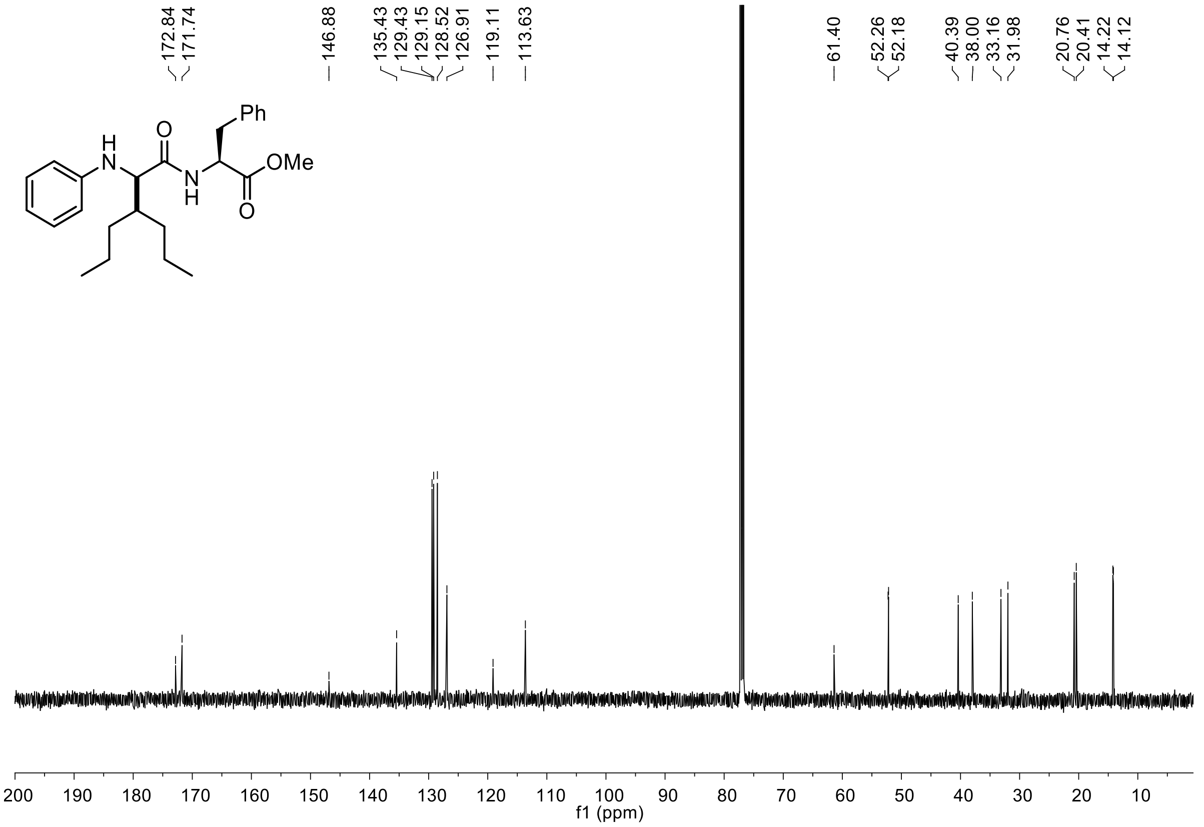

^{1}H NMR spectrum of 45 ($CDCl_3$, 500 MHz)

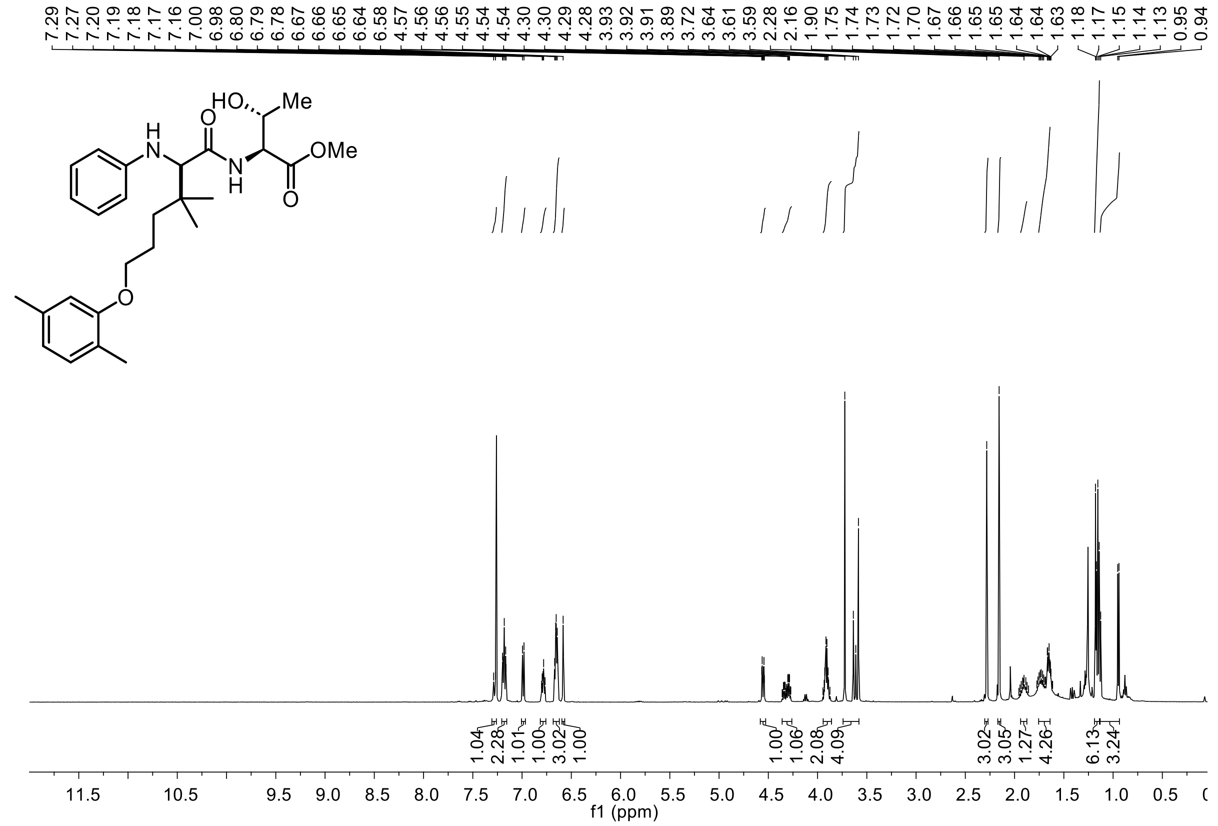

^{13}C{^{1}H} NMR spectrum of 45 ($CDCl_3$, 126 MHz)

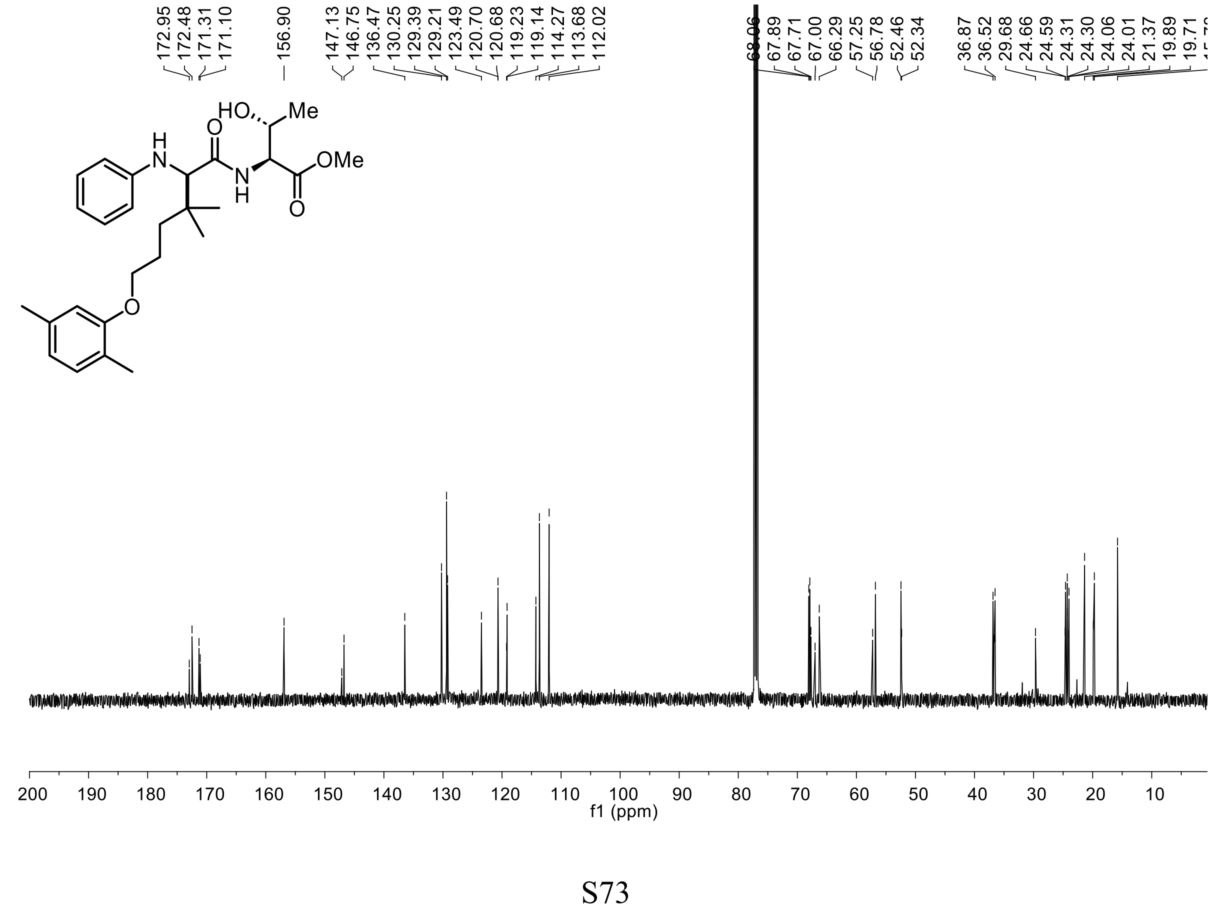

^{1}H NMR spectrum of 46 ($CDCl_3$, 500 MHz)

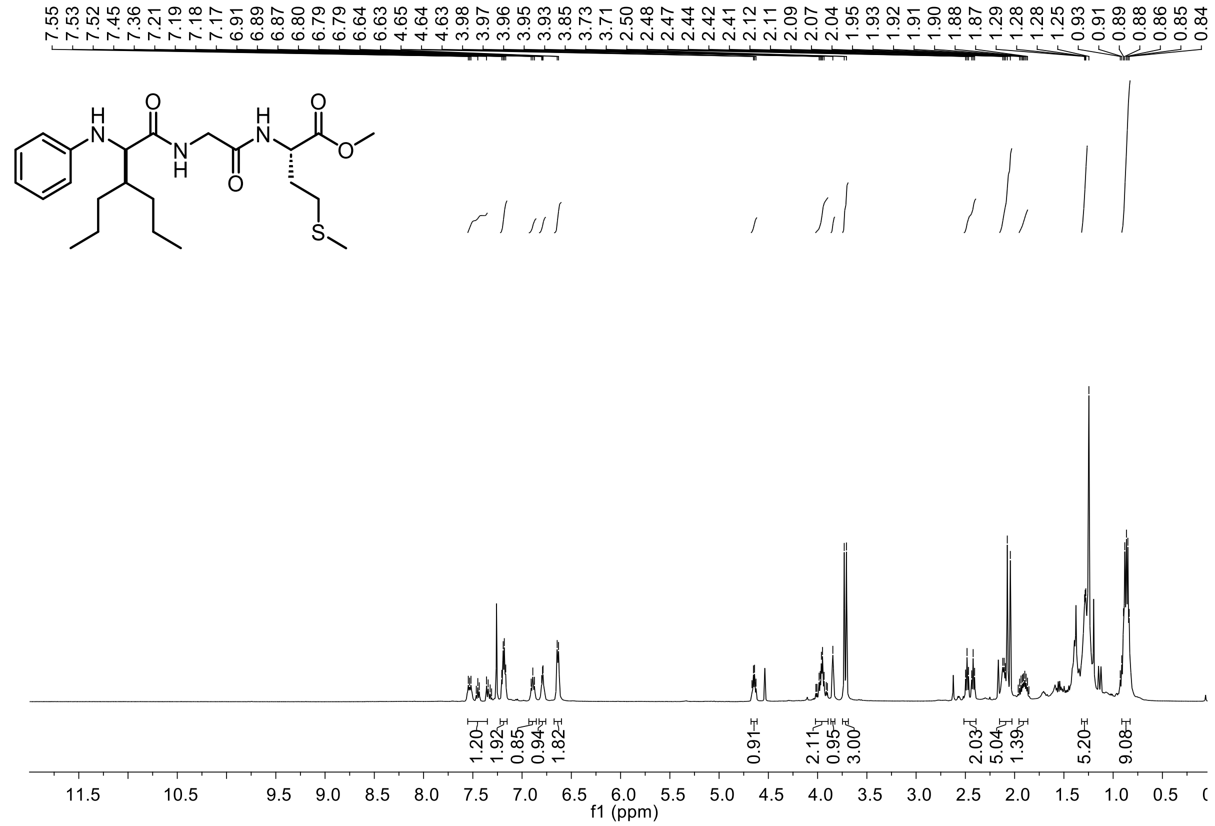

^{13}C{^{1}H} NMR spectrum of 46 ($CDCl_3$, 126 MHz)

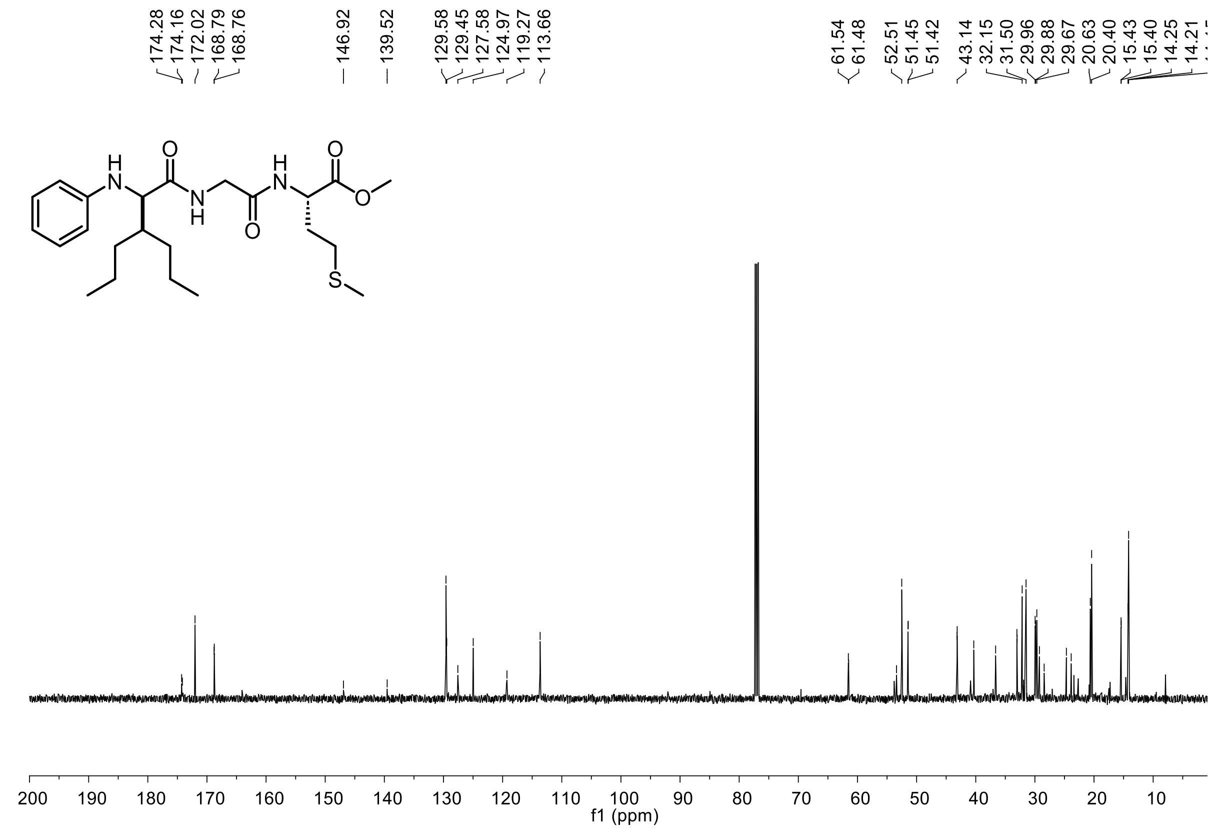

Supplement: SC-016-D5SC07730C-s001 [file SC-016-D5SC07730C-s001.pdf]
